# Cascade Reactions of Indigo with an Allenylic Reactant

**DOI:** 10.3390/molecules30142899

**Published:** 2025-07-08

**Authors:** Dyah U. C. Rahayu, Christopher Richardson, John B. Bremner, Paul A. Keller

**Affiliations:** School of Science, Molecular Horizons, University of Wollongong, Wollongong, NSW 2522, Australia; ducr647@uowmail.edu.au (D.U.C.R.); crichard@uow.edu.au (C.R.); jbremner@uow.edu.au (J.B.B.)

**Keywords:** indigo, cascade reaction, allenylic electrophile, benzoindolonaphthyridinedione, benzoazepinopyridoindolediones

## Abstract

The base-enabled reaction of buta-2,3-dien-1-yl methanesulfonate with the readily available and cheap dye indigo resulted in the convenient one-pot synthesis of benzoindolonaphthyridinedione and benzoazepinopyridoindolediones, with the latter representing two novel heterocyclic scaffolds. Despite the low yields, the allenylic alkylation of indigo significantly contributes to the new chemistry of this compound, providing new mechanistic insights and reactivity boundaries.

## 1. Introduction

Indigo **1** is an ancient dark blue pigment commonly used in the dyeing of blue jeans [1,2]. The inexpensive and abundant indigo **1** also serves as a building block in organic chemistry to generate new, diverse, and complex heterocyclic scaffolds after base-induced activation and subsequent exposure to electrophiles [3,4]. With its core of nucleophilic amino and electrophilic carbonyl group reaction sites conjugated through a carbon-carbon double bond, the important natural product indigo **1** is well set up as a testbed to investigate one-pot cascade reactions. In addition, the degree of reactivity in this central core motif offers a rich and complex array of reactivity that can shift during the reaction pathway, thus leading to new complex chemistries of indigo **1** [3,5].

**Scheme 1:**
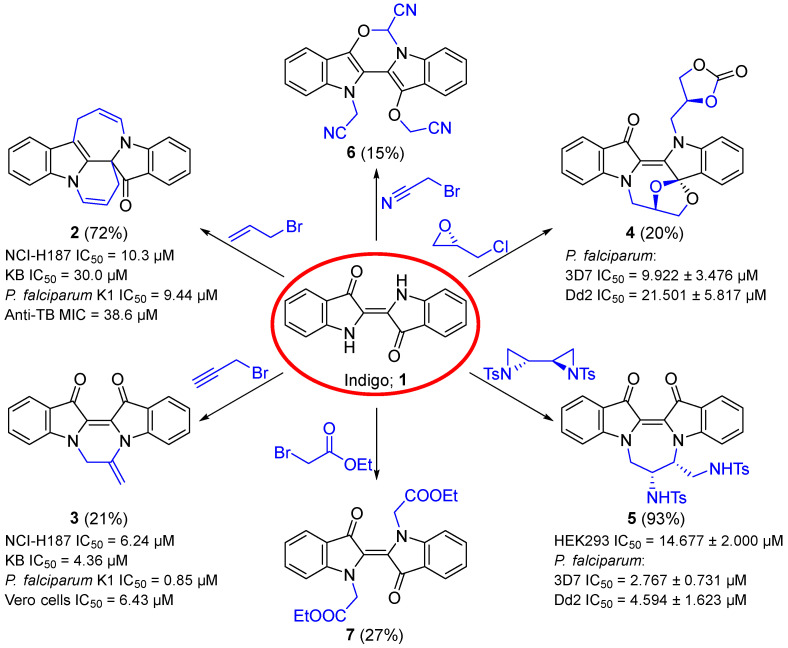
Examples of heterocyclic systems arising from indigo **1** cascade reactions with some reported biological activities. NCI-H187 = NCI small cell lung carcinoma, KB = KB oral cavity cancer, HEK293 = human embryonic kidney 293 cells, Vero cells: African green monkey kidney tissue, malaria = *Plasmodium falciparum* drug-resistant K1 or Dd2 strain and drug-susceptible 3D7 strain, anti-TB = *Mycobacterium tuberculosis*.

Several studies have reported outcomes of such one-pot multi-step reactions that offer rapid routes to new heterocyclic systems relevant to medicinal and materials chemistry [5,6,7,8,9,10,11,12]. Reactants used in such reactions include those with alkyl [6,7], allyl [8,9], or propargyl [5,10] entities, ring-strained electrophiles [11], and terminal electrophilic sites [10,12,13] with an appropriate leaving group to initiate the reaction pathways from base-promoted indigo **1** *N*-alkylation (Scheme 1). For instance, the allylation and propargylation of indigo **1** provided a robust methodology to generate pyridoindolo-azepinoindolone **2** and pyrazinodiindolinone **3**, respectively [5,8,9,10]. Also investigated were the reactions of indigo **1** with ring-strained electrophiles, such as oxirane and biaziridine, with ring opening forming oxazocinodiindolone **4** and diazepinodiindoledione **5**, respectively [11]. Further, it was also revealed that reactions of indigo **1** with electrophiles that contained an electron-withdrawing substituent, such as bromoacetonitrile and ethyl bromoacetate, afforded oxazinodiindole **6** and *N*,*N*′-disubstituted indigo **7**, respectively, where the latter served as an intermediate to access spirocyclic and biindigoid scaffolds [10,12]. The resulting polyheterocyclic compounds have been shown to exhibit intriguing biological activities, such as antiplasmodial, antitubercular, and anticancer activities in vitro, as well as promising photophysical properties with high fluorescence quantum yields, readily accessible triplet states, and the ability to tune thermal stability as photoswitches [5,6,7,9,10,11].

**Scheme 2:**
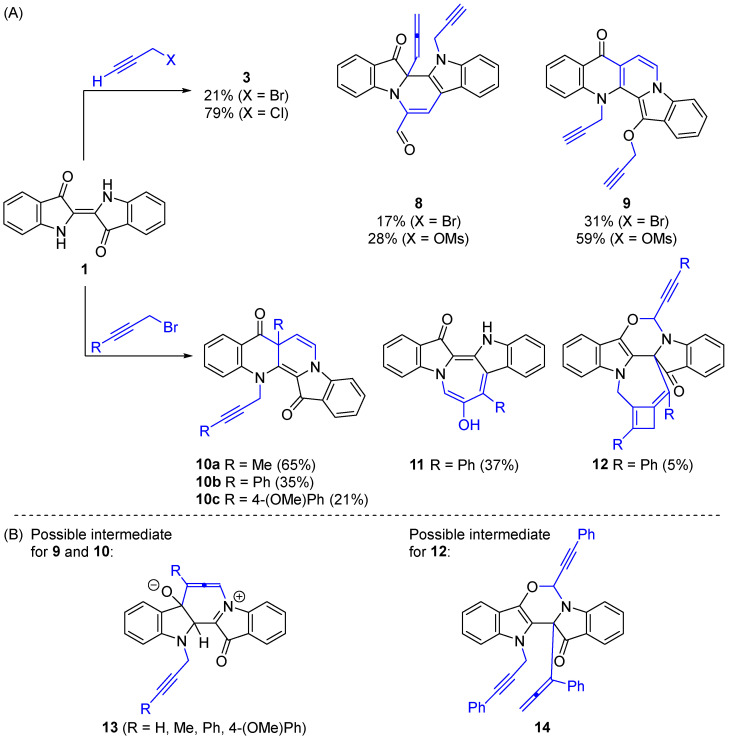
Examples of the structurally diverse heterocycles accessible from modified substitution patterns and leaving groups on the electrophiles in indigo **1** propargylations (**A**) and the possibility of allenic moieties generated in situ (**B**).

The structural outcomes of these reactions can be modified by using different leaving groups and changing the substitution pattern on the electrophiles [8,9,10,11]. For example, the use of propargyl bromide produced three heterocycle scaffolds: pyrazinodiindole **3**, pyridodiindole **8**, and the fused naphthyridine **9**. The use of propargyl chloride or mesylate yielded one (**3**) or two (**8** and **9**) heterocyclic systems, respectively. In addition, utilizing more substituted propargyl bromide derivatives, such as methyl-, phenyl-, and 4-methoxyphenylpropargyl bromide, led to the synthesis of naphthyridinone derivatives **10**, and with the use of phenylpropargyl bromide, azepinodiindole **11** and oxazinoazepine **12** motifs were accessible (Scheme 2A) [5,10]. Interestingly, the proposed mechanisms for the formation of **9**, **10**, and **12** involve the possible allenic intermediates **13** and **14** generated in situ (Scheme 2B). These allenic intermediates have demonstrated the ability to access structurally diverse heterocycles and drive further cascade cyclization reactions, producing unprecedented polyheterocyclic generations.

Therefore, it is of interest to use an allenylic reactant for further exploration of the *N*-alkylation-initiated chemistry of indigo **1**. The cumulative double bond structure of the allenic moiety incorporates a central *sp*-hybridized carbon that possesses higher reactivity compared with alkenes or alkynes, offering the prospect of new types of multi-step pathways to new heterocyclic systems [14,15]. The allenic central carbon site is more electron-rich with a terminal alkyl or an electron-donating group, but is electron-deficient with an electron-withdrawing group attached; and with a terminal metal attached, the end carbon sites are also electron-rich. Thus, these allene groups could give rise to a versatile and diverse chemistry [14,16,17,18].

While an interesting phosphine-catalyzed (3 + 2) annulation of isoindigos with allenes containing a directly attached electron-withdrawing group has been published [19], as far as we can ascertain, no reactions of any allenic derivatives with indigo **1** have been reported. As we required both an allenic group and an appropriate leaving group on a *sp^3^* carbon for these studies, we investigated the base-mediated reaction of indigo **1** with the reactive allene derivative buta-2,3-dien-1-yl methanesulfonate [20]. These outcomes, plus some proposed mechanistic pathways, are reported in this paper.

## 2. Results and Discussion

### 2.1. The Buta-2,3-dien-1-ylation of Indigo

**Scheme 3:**
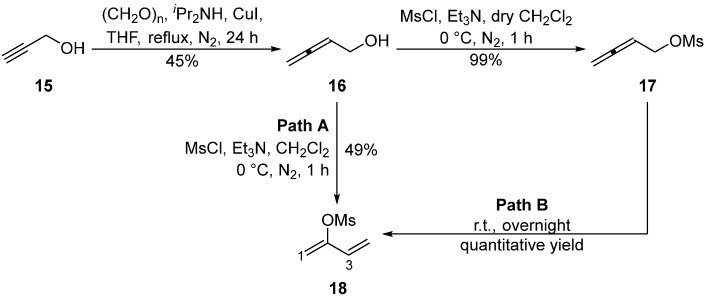
Synthesis and rearrangement of buta-2,3-dien-1-yl methanesulfonate **17**.

The allenylic electrophile was synthesized in two steps by converting alkyne **15** to allenol **16**, followed by mesylation to generate buta-2,3-dien-1-yl methanesulfonate **17** in 45% yield over two steps (Scheme 3) [20]. Interestingly, the new 2-functionalized 1,3-diene **18** was isolated as a by-product if non-anhydrous dichloromethane was used as the solvent during the mesylation step with buta-2,3-dien-1-ol **16** (**Path A**, Scheme 3) or if the buta-2,3-dien-1-yl mesylate **17** was stored overnight at room temperature (**Path B**, Scheme 3).

**Scheme 4:**
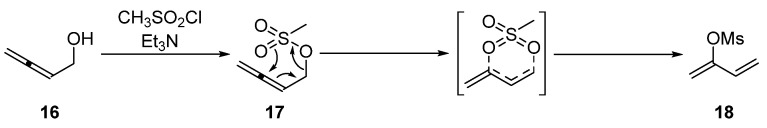
Plausible concerted mechanism for the rearrangement of buta-2,3-dien-1-yl methanesulfonate **17** to **18**.

Buta-1,3-dien-2-yl methanesulfonate **18** was isolated as a colorless oil. Analysis of the HSQC spectrum revealed two pairs of diastereotopic terminal alkene proton resonances at δ_H_ 5.06 and 5.27 ppm, assigned to H1, correlating to one carbon resonance at δ_C_ 106.4 (C1) ppm. Signals at δ_H_ 5.31 and 5.62 ppm were assigned to H4 and correlated to δ_C_ 117.7 (C4) ppm. A plausible mechanism for the rearrangement of **17** to **18** arises from the instability of 2,3-dienes and can be explained through a concerted methanesulfonyl group migration involving a six-membered cyclic transition (Scheme 4). Such rearrangements have been reported previously in more substituted allenes [21,22,23,24]. If moisture is present in the CH_2_Cl_2_, some methanesulfonic acid may be formed, which could facilitate the displacement of water from **16** with attack on the central allenic carbon by the weakly nucleophilic methanesulfonate anion to give **18**. Ready access to the 2-functionalized 1,3-diene **18** could be synthetically useful in the context of other reactions [22,25,26].

With the availability of buta-2,3-dien-1-yl methanesulfonate **17**, a suspension of indigo **1** (1 eq.) in anhydrous DMF was sonicated for 60 min at room temperature to facilitate solubilization and then cannulated to a septum-equipped flask containing pre-dried Cs_2_CO_3_ (2 eq.) under a N_2_ atmosphere. The flask was then heated to 85–87 °C and stirred for 60 min to aid N-H deprotonation. TLC analysis showed that after 60 min of sonication and after 60 min of heating at 85–87 °C, no other products were observed. Buta-2,3-dien-1-yl mesylate **17** (5 eq.) was added and the reaction mixture stirred for 7 min, followed by an extensive work-up with multiple rounds of column and/or preparative thin layer chromatography to afford *N*-buta-2,3-dien-1-ylindigo (**19**, 16%), benzoindolonaphthyridinedione (**20**, 3%), and benzoazepinopyridoindolediones (**21**, 6%) and (**22**, <1%)—see Table 1, entry 1. Compounds **21** and **22** represent new heterocyclic systems.

*N*-Buta-2,3-dien-1-ylindigo **19** was isolated as a papery blue solid. Analysis of the HRMS-ESI spectrum revealed a peak at *m*/*z* 337.0942, assigned to the molecular formula C_20_H_14_N_2_O_2_Na, which corresponds to [M + Na]^+^, and confirmed the addition of one buta-2,3-dien-1-yl unit. Analysis of the NOESY spectrum showed a correlation of the resonance δ_H_ 5.14 (H1″) with 7.15 (H7) ppm, with no correlation observed between the *N*-methylene group and the free NH, supporting the presence of the transoid structure across the 2,2′-double bond. Furthermore, the persistence of the blue color, which would result from the presence of at least one H-bond between the NH and carbonyl group [9], is also consistent with the (*E*) stereochemistry in the *N*-monoalkylated indigo **19**.

Benzoindolonaphthyridinedione **20** was isolated as red needles, and under UV light (365 nm), its CH_2_Cl_2_ solution showed significant fluorescence. This ring-expanded molecule possesses the same heterocyclic core as in the previously reported derivatives from the propargylation of indigo **1** (**9** and **10**, Scheme 2) [5,10]. Analysis of the ^1^H NMR spectrum revealed resonances at δ_H_ 5.23 and 4.78 ppm assigned to the allenic H2″ and H4″ protons. A singlet at δ_H_ 1.61 ppm and a pair of doublets at δ_H_ 6.84 and 5.44 ppm (*J* = 7.9 Hz) were assigned to H1′, H6, and H7, respectively, indicating a second buta-2,3-dien-1-yl unit addition, but in this instance being incorporated into a ring. Analysis of the ^13^C NMR spectrum revealed two quaternary resonances at δ_C_ 193.6 and 178.1 ppm, assigned to the C8 and C14 carbonyls, respectively, indicating that no *O*-alkylation had occurred. Analysis of the NOESY spectrum showed a ^1^H NMR resonance at δ_H_ 5.00 ppm (H1b″), which was correlated to a resonance at δ_H_ 7.11 ppm, assigned to the H12 aromatic ring, confirming *N*-alkylation. The HRMS-ESI showed a peak at *m*/*z* 367.1445 assigned to [M + H]^+^ and was consistent with a molecular formula of C_24_H_19_N_2_O_2_.

**Figure 1:**
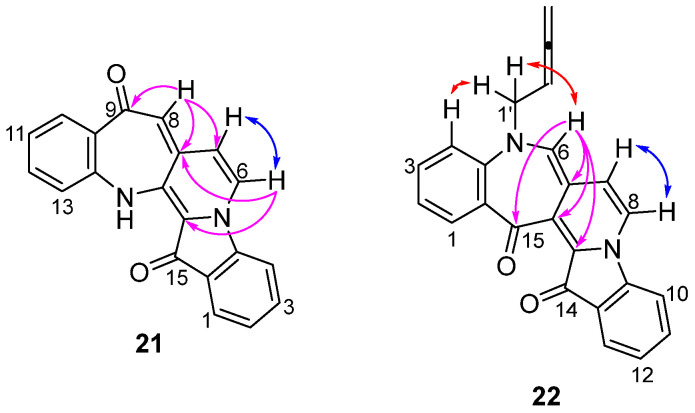
Key correlations in support of the structural elucidation of benzoazepinopyridoindolediones **21** and **22**. The correlations observed by COSY, NOESY, and HMBC are annotated in blue, red, and magenta, respectively.

Benzoazepino[2′,3′:3,4]pyridoindoledione **21** was isolated as a purple solid. Analysis of the HRMS-ESI spectrum showed a peak at *m*/*z* 313.0966 assigned to [M + H]^+^, confirming the molecular formula C_20_H_13_N_2_O_2_. Analysis of the ^1^H NMR spectrum revealed a singlet at δ_H_ 6.50 ppm assigned to H8 and a pair of doublets at δ_H_ 7.10 and 6.39 ppm (*J* = 2.8 Hz) assigned to H6 and H7, respectively, suggesting that the incorporation of buta-2,3-dien-1-yl unit as a new ring had occurred. Two downfield signals in the ^13^C NMR spectrum at δ_C_ 187.1 and 180.3 ppm were assigned to the carbonyl carbons C9 and C15, respectively, indicating no buta-2,3-dien-1-yl cyclization with the carbonyls. Analysis of the HMBC spectrum showed correlations of the ^1^H NMR resonance at δ_H_ 6.50 (H8) ppm with the ^13^C NMR resonances at δ_C_ 187.1, 126.5, and 120.5 ppm, which were assigned as C9, C7a, and C7, respectively. In addition, a doublet at δ_H_ 7.10 (H6) ppm revealed correlations to carbon resonances in the HMBC spectrum at δ_C_ 126.5 (C7a) and 126.9 (C14b) (Figure 1).

The skeletally isomeric benzoazepino[4′,3′:3,4]pyridoindoledione **22** was isolated as a red solid and displayed a significant fluorescence in CH_2_Cl_2_ solution under UV light (365 nm). Analysis of the ^1^H NMR spectrum of this molecule revealed two resonances at δ_H_ 5.24 and 4.93 ppm assigned to the allenic H2′ and H4′, indicating a buta-2,3-dien-1-yl substituent. Three proton resonances at δ_H_ 7.00, 7.11, and 7.88 ppm were assigned to H6, H7, and H8, respectively, indicative of a ring-embedded, second buta-2,3-dien-1-yl unit addition. Analysis of the HMBC spectrum showed correlations of the ^1^H NMR resonance at δ_H_ 7.00 (H6) with the ^13^C NMR resonances at δ_C_ 126.1, 130.4, 137.8, and 184.8 ppm, which were assigned as C6a, C14b, C14a, and C15, respectively. Analysis of the NOESY spectrum revealed key correlations between the aromatic resonance at δ_H_ 6.94 ppm, assigned to H4, and the H1′ methylene-allenic proton at δ_H_ 4.49 ppm, with the H1′ proton also correlating to the proton resonance at δ_H_ 7.00 (H6) ppm (Figure 1). The HRMS-ESI spectrum showed a peak at *m*/*z* 387.1123 assigned to [M + Na]^+^ and was consistent with a molecular formula of C_24_H_16_N_2_O_2_Na.

Efforts at optimizing this reaction are summarized in Table 1. Initial attempts targeted the synthesis of *N*-buta-2,3-dien-1-ylindigo **19** using a decreased reaction time (1 min), with the plan to use **19** as a starting material for subsequent alkylations. This resulted in decomposition to *N*-buta-2,3-dien-1-ylisatin **23** and tryptanthrin **24** in 24% and 15% yields, respectively, due to exposure to oxygen during quenching the reaction, with only a 2% yield of **19** (Table 1, entry 2). The decomposition products were isolated as yellow solids for the new *N*-buta-2,3-dien-1-ylisatin **23** and the known tryptanthrin **24**. The structure of **23** was confirmed by single-crystal X-ray analysis. A related but more highly substituted chiral isatin derivative has been synthesized from the Pd-catalyzed *N*-allenylic alkylation of isatin [27]. The lack of isolation of the side products **23** and **24** in the 7 min reaction (Table 1, entry 1) can be attributed to **23** and **24** being present in the reaction mixture in the early stages, but the longer reaction time would then allow for further base-initiated and oxidative cleavage processes to take place to give baseline material, as observed by TLC analysis.

The repetition of the 1 min reaction using double equivalence of Cs_2_CO_3_ (4 eq.) showed similar outcomes to the previous entry, with an extensive array of products leading to an inseparable mixture (Table 1, entry 3). Furthermore, a longer reaction time (7 min) with 4 eq. of Cs_2_CO_3_ (Table 1, entry 4) furnished a comparable result to Table 1, entry 1. At this stage, the equivalents of the base used showed no significant impact on the cascade reactions. However, the previously reported indigo **1** allylations and propargylations using 4 equivalents of base achieved better outcomes [5,8,9,10,11,12]. Thus, 4 eq. of Cs_2_CO_3_ was applied for the rest of the optimizations.

In order to maximize the initial deprotonation step, the alkaline indigo **1** suspension was heated at a slightly higher temperature (90 °C) before the addition of electrophile **17** (Table 1, entries 5–6). A significant decrease in the yield of *N*-buta-2,3-dien-1-ylindigo **19** (2%) was observed when this mixture was treated with an additional 3 eq. of electrophile **17** at 85–87 °C combined with a longer reaction time (15 min) with insignificant changes in the cascade products yields (3–6% for **20**–**22**) (Table 1, entry 5). Lowering the reaction temperature (from 85–87 to 70 °C) without an extra amount of electrophile **17** resulted in a little indigo **1** consumption as determined by TLC analysis (15 min). However, extending the reaction to 55 min led to an inseparable mixture of compounds due to decomposition (Table 1, entry 6). The absence of **20** and **22** indicated that the lower temperature generated products derived from *N*-monoalkylation, i.e., **19** (<1%) and **21** (4%).

The deprotonation time at 90 °C was increased from 60 to 90 min, and the reaction temperature was increased (Table 1, entries 7–9). Treatment of the resulting mixture with buta-2,3-dien-1-yl mesylate **17** at 75 °C for 10 min resulted in the isolation of **19** (4%), **20** (9%), and **21** (5%) (Table 1, entry 7). A shorter reaction time of 7 min afforded **19** (9%), **20** (3%), and **21** (3%), opposite yield trends to the previous attempt, suggesting *N*-buta-2,3-dien-1-ylindigo **19** served as an intermediate for further cascade outcomes (Table 1, entry 8). Heating the reaction to 80 °C for 7 min gave **19** (6%), **20** (3%), and **21** (8%), suggesting that the cyclization of *N*-monoalkylated indigo **19** to generate **21** is more competitive than the second *N*′-alkylation to produce **20** (Table 1, entry 9). The absence of **22** indicated that the compound formation followed more complex cyclization pathways, which require more energy (see mechanistic discussion).

All the efforts in the reaction optimization evoked the possibility that the greater stoichiometric use of buta-2,3-dien-1-yl mesylate **17** (5 eq.) likely enabled further reaction pathways, leading to the poor yields of the cascade products. Therefore, control of the reaction outcomes was attempted by decreasing the equivalents of the electrophile **17** (2.4 eq.) (Table 1, entry 10). This was realized by using 1.2 eq. of the base with a shorter deprotonation time (30 min) at 85 °C and a minimal work-up to avoid decomposition, however, this attempt resulted in a 48% yield of unreacted indigo **1** with the isolation of **19** (8%), **20** (1%), and **21** (4%). The poor solubility of indigo **1** limits the number of bases and solvents that can be used in these reactions [5].

As a result of these low yields (Table 1), trying to decrease the reactivity of indigo **1** by substituting one of the free NH groups in indigo **1** could possibly increase the yield of cascade products. It was hypothesized that more limited reaction sites would result in better reaction control and lead to a more selective formation of cascade outcomes. Therefore, *N-*monosubstituted methyl indigo **25** was prepared from indigo **1** in the presence of base and methyl iodide, giving a blue papery solid in 54% yield [7,10].

The extended investigation of the buta-2,3-dien-1-ylation of *N*-methylindigo **25** started with conditions based on Table 1—entry 1, with modifications on the reduced time for sonication (5 min) and deprotonation (30 min) due to the significantly higher solubility of **25** relative to the parent indigo **1** and decreased equivalents of buta-2,3-dien-1-yl methanesulfonate **17** (3 eq.) with only a single NH site in **25** (Table 2, entry 1). Following work-up, a sequence of silica gel chromatographic separations yielded benzoindolonaphthyridinedione (**26**, crude, 8%) with much unreacted *N*-methylindigo (**25**, 58%), and unidentified decomposition products on the baseline by TLC analysis.

Compound **26** was obtained as a red solid and showed significant fluorescence in CH_2_Cl_2_ solution under UV light (365 nm). Analysis of the ^1^H NMR spectra of **26** showed similar spectral properties to **20**, supporting a similar heterocycle scaffold, with a difference in the presence of a 3-proton singlet resonance at δ_H_ 3.72 ppm (H1″), instead of the *N*-buta-2,3-dien-1-yl substituent. Furthermore, the ^1^H NMR spectrum analysis of **26** revealed proton resonances at δ_H_ 1.53 ppm as a singlet, assigned to H1′, and at δ_H_ 6.87 and 5.52 ppm (*J* = 7.6 Hz) as a pair of doublets, assigned to H6 and H7, respectively. The ^1^H NMR spectrum indicated the presence of some minor impurities. However, with a total of <5 mg, further purification of compound **26** was precluded, and therefore, only a crude yield is reported. Analysis of the HRMS-ESI spectrum showed a peak at *m*/*z* 329.1293 assigned to [M + H]^+^ and was consistent with a molecular formula of C_21_H_17_N_2_O_2_.

In an attempt to minimize decomposition and increase the yield of **26**, a lower reaction temperature (75–80 °C) was used (Table 2, entry 2). A similar trend to the previous entry was observed at a 3 h reaction time, as monitored by TLC analysis, leading to an extra addition of buta-2,3-dien-1-yl mesylate **17** (2 eq.) and a longer reaction time of 24 h. However, this resulted in a lower yield of **26** (crude, 4%) and **25** returned (41%) due to more decomposition occurring. The reduced reaction temperature (from 85–87 to 75–80 °C) would likely slow any cascade reactions; however, more decomposition was likely to occur as a result of the prolonged reaction time.

Halving an equivalent of Cs_2_CO_3_ with a longer deprotonation time of 60 min was utilized (Table 2, entries 3–5). By using 5 eq. of electrophile **17** at 85–87 °C, this reaction showed a comparable result to the previous entries, even after additional electrophile **17** (2 eq.) was added during the 75 min reaction, with a 3% crude yield of **26** and a 24% yield of **25** returned (Table 2, entry 3). Increasing the time to 90 min with 7 eq. of **17** showed little change in the products by TLC analysis (Table 2, entry 4). A small conversion was also seen by TLC analysis when changing the solvent to THF (Table 2, entry 5). This result can be explained due to the poor solubility of Cs_2_CO_3_ in THF^†^, limiting the deprotonation of *N*-methylindigo 25. Extending this reaction to 21 h resulted in the decomposition of 25. (^†^ There was an imperceptible color change in the reaction mixture before the addition of electrophile 17 (blue → greenish blue), indicating that no or less deprotonation of *N*-methylindigo 25 occurred compared to using Cs_2_CO_3_ in DMF.)

These results suggested that *N*-methylindigo **25** is significantly less reactive than **1**, leading to a small consumption of **25**. Thus, a series of modifications were carried out, either replacing the leaving group of the electrophile **17** or changing the base (Table 2, entries 6–7). Under similar conditions to the earliest trial (Table 2, entry 1), adding KI to the reaction to convert mesylate to an iodide leaving group in situ resulted in an insignificant improvement based on TLC analysis (Table 2, entry 6). Furthermore, no major changes were found by TLC analysis when the reaction was repeated using KHMDS as a sterically hindered strong base in THF (Table 2, entry 7).

Based on the reaction observations, limiting the reaction sites by incorporating one *N*-methyl group in the indigo **1** core significantly impacted reactivity, and without a second NH group in the substrate, further cyclization pathways were blocked. The unstable nature of the 2,3-diene electrophile **17** was possibly also a factor in lowering yields of the indigo-based products.

### 2.2. Mechanistic Proposals for the Indigo Cascade Reactions

**Scheme 5:**
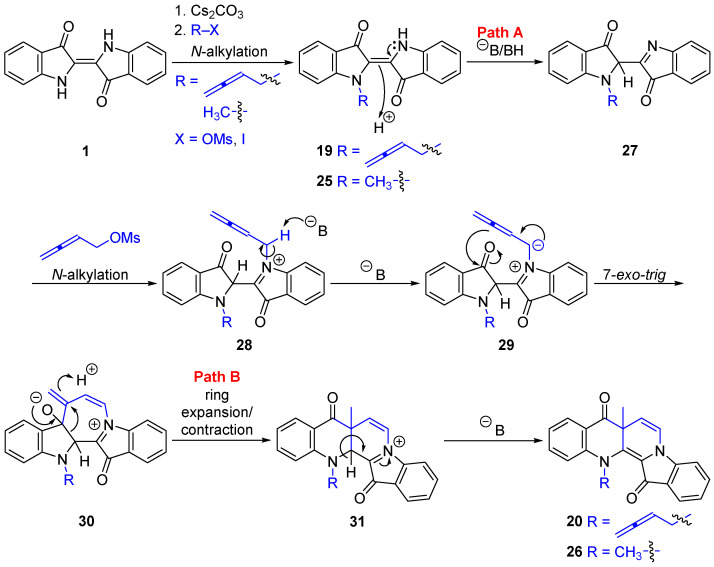
The proposed mechanism for benzoindolonaphthyridinediones **20** and **26** formation.

**Scheme 6:**
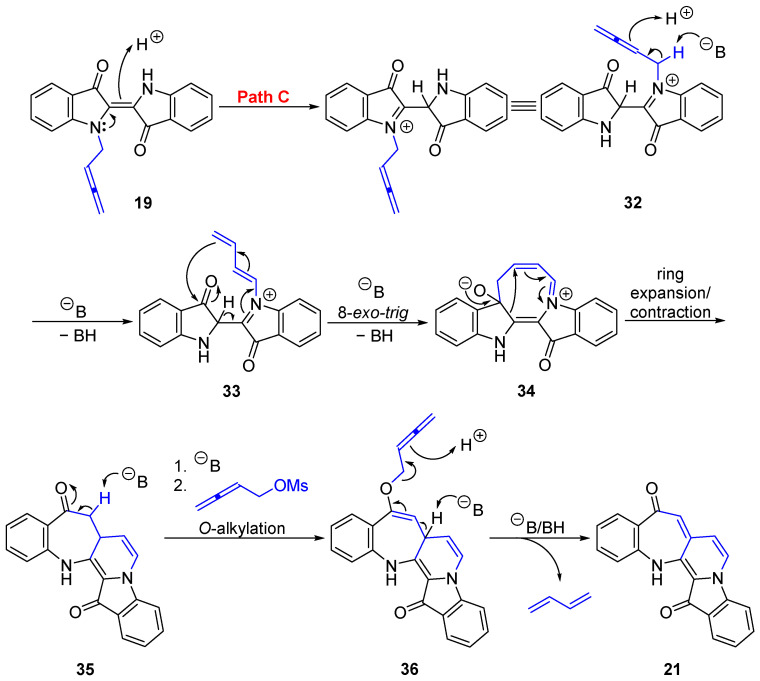
The proposed mechanism for the formation of benzoazepinopyridoindoledione **21**.

**Scheme 7:**
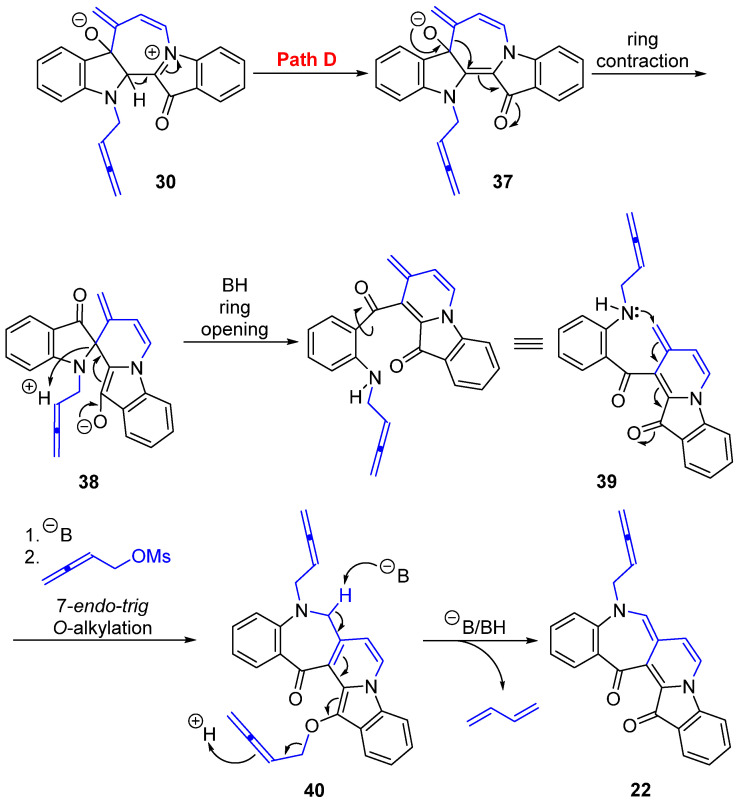
The proposed mechanism for the formation of benzoazepinopyridoindoledione **22**.

The proposed mechanisms for the cascade product outcomes from the addition of buta-2,3-dien-1-yl methanesulfonate **17** to indigo **1** are outlined in Scheme 5, Scheme 6 and Scheme 7. In these schemes, ^Ө^B/BH are most likely to be carbonate and bicarbonate entities. After the initial *N*-alkylation to generate *N*-monoalkylated indigo key products **19** and **25** (Scheme 5), prototropic tautomerism to give **27** (**Path A**), followed by a second *N*-alkylation, facilitates the formation of the *N*,*N*’-dialkylindigo intermediates **28**. Alternatively, compound **19** can be deprotonated, followed by a second *N*-alkylation and subsequent protonation of the double bond to give intermediate **28**. Deprotonation of the *N*-methylene group forms a stabilized ylid **29**, which undergoes a *7-exo-trig* cyclization with a neighboring indigoid carbonyl to generate a seven-membered heterocycle **30**. Importantly, intermediate **30** possesses the same heterocyclic skeleton as that proposed in intermediate **13** for **9** and **10** formation from the previous indigo **1** propargylations (Scheme 2) [5,10]. Further ring expansion/ring contraction to give **31** (**Path B**) and final deprotonation produces benzoindolonaphthyridinedione heterocycles **20** and **26**. The isolation of **20** and **26** from the buta-2,3-dien-1-yl addition to indigo **1** supports the previously reported proposed mechanisms for the propargylation of indigo **1** using 1-bromo-2-butyne [10].

The proposed mechanism for the formation of benzoazepinopyridoindoledione **21** starts from the key compound **19** (Scheme 6). *N*-Buta-2,3-dien-1-ylindigo **19** could undergo protonation to **32** (**Path C**) with subsequent base-induced isomerization to afford the 1,3-diene intermediate **33**. Intramolecular *8-exo-trig* cyclization onto the indolone carbonyl to give **34**, followed by ring expansion of one indoline unit with concomitant ring contraction of the 8-membered ring, would give the fused 7-membered intermediate **35**. Subsequent *O*-alkylallenylation to **36**, and a final deprotonation with buta-1,3-diene expulsion gives benzoazepinopyridoindoledione **21**. This compound contains one free NH in the structure and represents a new and unique complex heterocycle scaffold.

The benzoazepinopyridoindoledione **22** formation is derived from the key seven-membered ring intermediate **30** (Scheme 5; R = allenylmethyl) following **Path D **(Scheme 7). Proton loss would afford **37,** which is then set up for a ring contraction to give the spiro intermediate **38**. Ring opening then allows for rotation about the C-CO bond to give **39**, with groups appropriately positioned for a *7-endo-trig* cyclization through nitrogen attack on the terminal alkene, with further *O*-alkylallenylation to furnish **40**. Such ring contractions followed by ring opening have previously been demonstrated in the formation of pyrrolodione from indigo **1** propargylation using 3-phenylpropiolaldehyde [10]. In the final step, deprotonation drives the extended conjugation to release buta-1,3-diene and affords the novel structural motif of benzoazepinopyridoindoledione **22**. Mechanistically, the formation of all cascade outcomes from indigo **1** buta-2,3-dien-1-ylation is supported by prior studies under comparable reaction conditions and consistent with the unifying mechanistic proposals for cascade reactions of indigo, e.g., reaction of propargyl and/or allyl moieties with indigo **1** [5,8,9,10]. These findings also support the allenic intermediate formed from previous indigo **1** propargylations and further expand the wider understanding of the mechanistic pathways of indigo **1** cascade reactions.

**Scheme 8:**
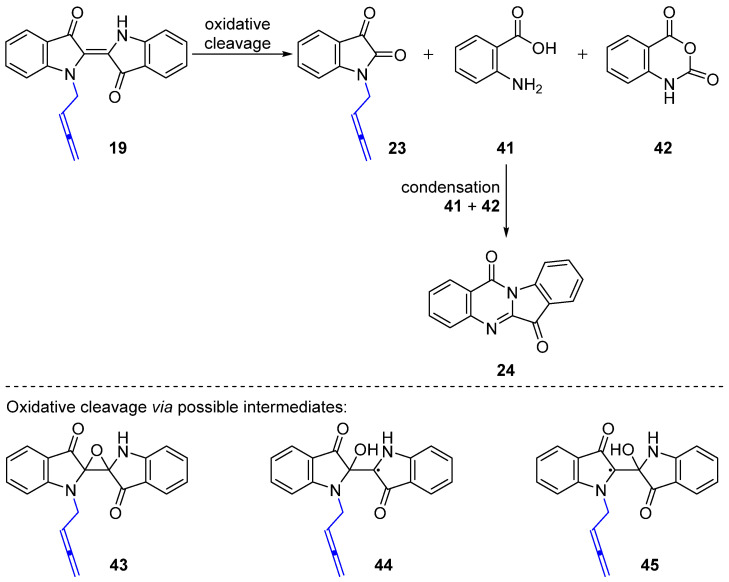
The proposed pathway of *N*-buta-2,3-dien-1-ylisatin **23** and tryptanthrin **24** formation via oxidative cleavage.

The *N*-buta-2,3-dien-1-ylisatin **23** and tryptanthrin **24** are likely to be formed from the oxidative cleavage of the *N*-allenylmethylindigo **19** under basic conditions with buta-2,3-dien-1-yl mesylate **17**, as described in Scheme 8. As previously reported, the decomposition of indigo **1** derivatives via oxidative cleavage can arise in the presence of oxidants or bases to generate isatin or derivatives, for example **23**, as well as anthranilic acid **41**, or isatoic anhydride **42**, which can further condense to yield tryptanthrin **24** [28,29]. Based on previous proposals [30,31,32] and with possible traces of atmospheric oxygen present during the reaction, the potential intermediates in the fragmentation process are suggested to be derived from initial triplet oxygen addition to the central double bond in **19**. This includes the epoxide **43**, and the stabilized radicals **44** and **45**, although these may also be formed by hydroxyl radical attack on **19**. A longer reaction time with excess buta-2,3-dien-1-yl mesylate **17** at a high temperature (>85 °C) is likely to contribute to the cleavage process in comparison with milder conditions (<85 °C), where no oxidation outcomes were observed.

## 3. Materials and Methods

### 3.1. Chemistry

Reagents and solvents obtained from commercial sources were used without purification unless otherwise stated. Buta-2,3-dien-1-yl methanesulfonate **17** was two-step synthesized as reported [20]. Methanesulfonyl chloride (MsCl), triethylamine (Et_3_N), potassium iodide (KI), and potassium bis(trimethylsilyl)amide (KHMDS, 0.1 M in tetrahydrofuran (THF)) were purchased from Merck (Sigma–Aldrich, St. Louis, MO, USA). Indigo **1** and cesium carbonate (Cs_2_CO_3_) were purchased from AK Scientific (Union City, CA, USA). Cs_2_CO_3_ was stored in a desiccator and pre-dried by regularly heating with a heat gun for an hour under a high vacuum. *N*-Methylindigo **25** was synthesized as reported with some modifications on the base used [7,10]. High-performance liquid chromatography (HPLC) grade dichloromethane (CH_2_Cl_2_) and THF were used, and all other solvents were purchased reagent grade from ChemSupply (Gillman, SA, Australia). THF was distilled over sodium/benzophenone ketyl or stored under molecular sieves (3 Å, 20% *m*/*v*) for 72 h. Dry CH_2_Cl_2_ was prepared by storing under 3 Å M.S. (10% *m*/*v*) for a day or accelerated by sonicating and N_2_ purging for an hour. Anhydrous DMF was purchased from Merck (Sigma-Aldrich, St. Louis, MO, USA) and was stored under a N_2_ atmosphere.

Reverse osmosis (RO) water used to prepare aqueous solutions and for extraction was obtained from a Millipore purification system. A brine solution was a saturated aqueous solution prepared from salt purchased from ChemSupply (Gillman, SA, Australia). Molecular sieves (3 Å M.S.) were activated in a furnace at 300 °C. The N_2_ used in reactions was passed through a drying tube filled with silica gel beads (blue → yellow indicator). Sonication was performed using a UC-S3200H Ultrasonic Cleaner (40 KHz). Thin-layer chromatography (TLC) was performed on aluminum-supported sheets (silica gel 60 F_254_, 200 μm thickness). Preparative TLC (PTLC) was performed using silica gel 60 coated with glass plates (0.5 mm thickness, 20 × 20 cm). Column chromatography was performed on silica gel 60 (40–63 μm, 230–400 mesh). Automated flash chromatography was accomplished using BUCHI C-815 Flash with iLOK^®^ Flash Cartridges (silica gel 60, 40–63 µm). Solvent extracts and chromatographic fractions were concentrated in vacuo by rotary evaporation (BUCHI Rotavapor R-300).

Melting points were determined using a Griffin A010106 and are uncorrected. ^1^H and ^13^C NMR spectra (CDCl_3_) were recorded at either 500 and 126 MHz (Bruker Avance NEO 500 MHz) or 400 and 101 MHz (Bruker Avance III Nanotray 400 MHz), respectively. Chemical shifts (δ) were reported as parts per million (ppm) relative to TMS (δ = 0.00 ppm). Multiplicities are reported as singlets (s), doublets (d), quintets (quint), doublet of doublets (dd), doublet of triplets (dt), doublet of quartets (dq), triplet of doublets (td), triplet of triplets (tt), doublet of doublet of doublets (ddd), doublet of doublet of triplets (ddt), multiplets (m), or apparent (appt). Coupling constants (*J*) are reported as the average values in Hertz (Hz). Infrared (IR) spectra were collected on a Bruker Vertex 70 FTIR, either as neat or as thin films of the samples. UV-vis absorption spectra (CH_2_Cl_2_) were recorded using a Shimadzu UV-1800 double-beam spectrophotometer at room temperature. Low-Resolution Mass Spectrometry (LRMS) Electron Impact (EI) and Electrospray Ionisation (ESI) were performed on Shimadzu QP5050 and Shimadzu LCMS-2020, respectively, with ion mass-to-charge values (*m*/*z*) reported as percentage of relative abundances in parentheses. High-Resolution Mass Spectrometry (HRMS) was performed using a Waters Quadrupole-Time of Flight (QTOF) Xevo with a Leucine–Enkephalin standard.

### 3.2. Synthesis of the Electrophile

#### 3.2.1. Synthesis of Buta-2,3-dien-1-ol **16**

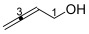



Buta-2,3-dien-1-ol **16** was synthesized as reported [20] in 45% yield (1.39 g, 19.83 mmol) as a colorless oil. ^1^H NMR (400 MHz, CDCl_3_) δ 5.32 (1H, quint, *J* = 6.4 Hz, H2), 4.83 (2H, dt, *J* = 6.4, 2.9 Hz, H4), 4.12 (2H, dt, *J* = 6.4, 2.9 Hz, H1), 3.04 (1H, s, OH). ^13^C NMR (101 MHz, CDCl_3_) δ 208.1 (C3), 90.8 (C2), 76.8 (C4), 60.2 (C1). FTIR (neat) *ν*_max_ 3314 (bm), 2931 (m), 2873 (m), 1955 (m), 1647 (m), 1438 (m), 1362 (m), 1208 (m), 1117 (m), 1007 (s), 843 (s), 532 (m) cm^−1^. LRMS-EI (100%) *m*/*z* 70 [M]^+^. The spectroscopic data were consistent with those reported in [20].

#### 3.2.2. Synthesis of Buta-2,3-dien-1-yl Methanesulfonate **17**

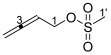



Buta-2,3-dien-1-yl methanesulfonate **17** was synthesized as reported [20] in 99% yield (0.67 g, 4.52 mmol) as a colorless oil. ^1^H NMR (400 MHz, CDCl_3_) δ 5.36 (1H, tt, *J* = 7.4, 6.6 Hz, H2), 4.95 (2H, dt, *J* = 6.6, 2.1 Hz, H4), 4.74 (2H, dt, *J* = 7.4, 2.1 Hz, H1), 3.03 (3H, s, H1′). ^13^C NMR (101 MHz, CDCl_3_) δ 210.6 (C3), 85.4 (C2), 77.4 (C4), 67.9 (C1), 38.3 (C1′). FTIR (neat) *ν*_max_ 3036 (m), 3020 (m), 2933 (m), 2853 (m), 1956 (m), 1348 (s), 1168 (s), 968 (m), 919 (s), 810 (m), 748 (m), 525 (s), 489 (s) cm^−1^. LRMS-EI (100%) *m*/*z* 148 [M]^+^. The spectroscopic data were consistent with those reported in [20].

#### 3.2.3. Synthesis of Buta-1,3-dien-2-yl Methanesulfonate **18**

Method 1: MsCl (1.33 g, 0.90 mL, 11.63 mmol) was added dropwise to a solution containing buta-2,3-dien-1-ol **16** (0.81 g, 11.57 mmol) and Et_3_N (2.40 g, 3.3 mL, 23.68 mmol) in CH_2_Cl_2_ (60 mL; non-anhydrous) at 0 °C under a N_2_ atmosphere. The reaction mixture was stirred at 0 °C for 1 h, quenched with water (60 mL), and extracted with Et_2_O (3 × 60 mL). The combined organic layers were washed with 5% (*v*/*v*) aqueous HCl (3 × 60 mL) and brine solution (3 × 60 mL), dried (MgSO_4_), and concentrated in vacuo (T ≤ 25 °C) to afford buta-1,3-dien-2-yl methanesulfonate **18** (0.85 g, 5.74 mmol, 49%) as a colorless oil.

Method 2: buta-2,3-dien-1-yl methanesulfonate **17** (0.51 g, 3.47 mmol) was kept at room temperature overnight to afford buta-1,3-dien-2-yl methanesulfonate **18** (quantitative yield) as a colorless oil.

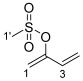



^1^H NMR (400 MHz, CDCl_3_) δ 6.26 (1H, dd, *J* = 17.1, 10.9 Hz, H3), 5.62 (1H, dq, *J* = 17.1, 0.7 Hz, H4a), 5.32 (1H, ddt, *J* = 10.9, 1.5, 0.7 Hz, H4b), 5.27 (1H, ddd, *J* = 2.5, 1.5, 0.7 Hz, H1a), 5.06 (1H, d, *J* = 2.5 Hz, H1b), 3.14 (3H, s, H1′). ^13^C NMR (101 MHz, CDCl_3_) δ 151.1 (C2), 130.4 (C3), 117.7 (C4), 106.4 (C1), 37.9 (C1′). FTIR (neat) *ν*_max_ 2920 (s), 2853 (s), 1709 (m), 1456 (s), 1362 (s), 1174 (s), 1038 (m), 949 (m), 523 (s) cm^−1^. LRMS-EI (100%) *m*/*z* 148 [M]^+^. HRMS-ESI *m*/*z* for C_5_H_9_O_3_S calculated 149.0272 [M + H]^+^, found 149.0273.

### 3.3. General Procedure for the Reaction of Indigo with Buta-2,3-dien-1-yl Methanesulfonate

A suspension of indigo **1** (1.0 eq.) in anhydrous DMF (40 mL/mmol) was sonicated for 60 min under a N_2_ atmosphere. The resulting suspension was transferred using a cannula to a flask containing pre-dried Cs_2_CO_3_ (1.2–4.0 eq.) and plunged into a pre-heated oil bath at 85–90 °C under N_2_ with stirring. After 30–90 min, buta-2,3-dien-1-yl methanesulfonate **17** (2.4–5.0 eq.) was added, and the reaction mixture was stirred for 1–55 min at 70–87 °C (the addition of 3 × 1.0 eq. of electrophile **17** was carried out only for entry 5 (Table 1)). The reaction was then quenched in an ice bath (400 mL/mmol) and filtered after the ice melted (Table 1, entries 1–9). The filtrate was extracted with EtOAc (3 × 60–100 mL/mmol) and washed with brine (3 × 60–100 mL/mmol) and distilled water (5 × 60–100 mL/mmol). The organic layer was collected, dried (Na_2_SO_4_), and concentrated in vacuo. In addition, the precipitate was washed with *n*-hexane (3 × 10 mL/mmol), dissolved in EtOAc (60–100 mL/mmol), and concentrated in vacuo. A different workup procedure was also used without filtration after the ice melted (Table 1, entry 10) as follows: the reaction outcome was directly transferred to a separatory funnel and extracted with CH_2_Cl_2_ (3 × 100 mL/mmol), brine (3 × 100 mL/mmol), and distilled water (5 × 100 mL/mmol). The organic layer was collected, dried (Na_2_SO_4_), and concentrated in vacuo. In a separate separation and purification, either the filtrate or the precipitate was subjected to column chromatography using 10% → 50% EtOAc/n-hexane (dry loading method with 20 × sample weight for silica absorbed and 100–200 × sample weight for silica column) to give five major fractions (F_1_–F_5_) including baseline decomposition. Fraction F_1_ contained an intense blue papery solid **19**. Compound **20**, as red needles, was isolated from further silica gel chromatography of fraction F_3_ using 20% → 60% EtOAc/n-hexane (dry loading method with 20 × sample weight for silica absorbed and 100–200 × sample weight for silica column). Furthermore, fraction F_5_ was subjected to another column chromatography using 30% → 70% EtOAc/n-hexane (dry loading method with 20 × sample weight for silica absorbed and 100–200 × sample weight for silica column) to obtain an intense purple solid **21**. Compound **22** was collected as a red waxy solid by multiple rounds of PTLC (30% EtOAc/n-hexane) of fraction F_4_. In addition, fraction F_2_ was subjected to further silica gel chromatography of fraction F_3_ using 20% → 60% EtOAc/n-hexane (dry loading method with 20 × sample weight for silica absorbed and 100–200 × sample weight for silica column) to yield two sub-fractions (F_2.1_ and F_2.2_). Compound **23**, as a yellow needle crystal, was obtained from trituration (5% EtOAc/n-hexane) followed by fractional crystallization (20% CH_2_Cl_2_/*n*-hexane) of sub-fraction F_2.1_, while compound **24** was collected from sub-fraction F_2.2_ with multiple attempts of trituration (5% EtOAc/*n*-hexane) as a yellow papery solid.

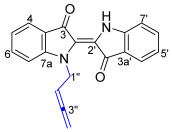

*(E)-1-(Buta-2,3-dien-1-yl)-[2,2′-biindolinylidene]-3,3′-dione* **19**

Compound **19** was isolated as a blue papery solid. R*_f_* (30% EtOAc/*n*-hexane) 0.70. mp. 144–146 °C. ^1^H NMR (400 MHz, CDCl_3_) δ 10.67 (1H, s, NH), 7.74 (1H, ddd, *J* = 7.6, 1.3, 0.7 Hz, H4), 7.69 (1H, appt. ddd, *J* = 7.6, 1.3, 0.8 Hz, H4′), 7.54 (1H, ddd, *J* = 8.4, 7.6, 1.3 Hz, H6), 7.47 (1H, ddd, *J* = 8.2, 7.6, 1.3 Hz, H6′), 7.15 (1H, d, *J* = 8.4 Hz, H7), 7.03 (1H, appt. td, *J* = 7.6, 0.7 Hz, H5), 7.00 (1H, dt, *J* = 8.2, 0.8 Hz, H7′), 6.95 (1H, appt. td, *J* = 7.6, 0.8 Hz, H5′), 5.29 (1H, quint, *J* = 6.3 Hz, H2″), 5.14 (2H, dt, *J* = 6.3, 2.9 Hz, H1″), 4.71 (2H, dt, *J* = 6.3, 2.9 Hz, H4″). ^13^C NMR (101 MHz, CDCl_3_) δ 208.9 (C3″), 189.9 (C3), 187.2 (C3′), 152.9 (C7a), 151.5 (C7a’), 136.1 (C6′), 135.8 (C6), 125.6 (C2), 124.8 (C4′), 124.1 (C4), 122.8 (C3a′), 121.2 (C3a), 120.9 (C5), 120.7 (C5′), 120.2 (C2′), 111.9 (C7′), 111.4 (C7), 87.1 (C2″), 77.2 (C4″), 46.0 (C1″). FTIR (thin film) *ν*_max_ 3287 (m), 1955 (m), 1637 (m), 1608 (s), 1466 (s), 1384 (m), 1300 (s), 1169 (m), 1069 (s), 1036 (s), 1013 (m), 756 (m) cm^−1^. UV-vis (CH_2_Cl_2_) λ_max_/nm (ε, M^−1^ cm^−1^) 249 (6835), 290 (9938), 627 (5893). LRMS-ESI (10%) *m*/*z* 315 [M + H]^+^; HRMS-ESI *m*/*z* for C_20_H_14_N_2_O_2_Na calculated 337.0953 [M + Na]^+^, found 337.0942.

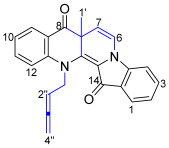

*13-(Buta-2,3-dien-1-yl)-7a-methylbenzo[b]indolo[1,2-h][1,7]naphthyridine-8,14(7aH,13H)-dione* **20**

Compound **20** was isolated as a red crystalline solid (needles). R*_f_* (30% EtOAc/*n*-hexane) 0.60. mp. 140–142 °C. ^1^H NMR (400 MHz, CDCl_3_) δ 7.93 (1H, dd, *J* = 8.0, 1.7 Hz, H9), 7.73 (1H, ddd, *J* = 8.1, 1.3, 0.8 Hz, H1), 7.53 (1H, td, *J* = 7.3, 1.7 Hz, H11), 7.51 (1H, td, *J* = 7.2, 1.3 Hz, H3), 7.11 (1H, dt, *J* = 8.0, 0.8 Hz, H12), 7.10 (1H, d, *J* = 8.1 Hz, H4), 6.99 (1H, ddd, *J* = 8.0, 7.3, 0.8 Hz, H10), 6.98 (1H, ddd, *J* = 8.1, 7.2, 0.8 Hz, H2), 6.84 (1H, d, *J* = 7.9 Hz, H6), 5.44 (1H, d, *J* = 7.9 Hz, H7), 5.23 (1H, quint, *J* = 6.8 Hz, H2″), 5.00 (1H, ddt, *J* = 15.9, 6.8, 2.6 Hz, H1b″), 4.78 (2H, m, H4″), 4.56 (1H, ddt, *J* = 15.9, 6.8, 2.6 Hz, H1a″), 1.61 (3H, s, H1′). ^13^C NMR (101 MHz, CDCl_3_) δ 209.8 (C3″), 193.6 (C8), 178.1 (C14), 146.7 (C4a), 144.5 (C12a), 139.0 (C13a), 135.5 (C11), 134.9 (C3), 128.7 (C9), 124.5 (C1), 122.6 (C8a), 121.3 (C13b), 120.8 (C10), 120.5 (C2), 120.0 (C6), 119.8 (C14a), 116.4 (C4), 108.7 (C12), 103.4 (C7), 86.3 (C2″), 77.2 (C4″), 52.3 (C1″), 48.6 (C7a), 29.7 (C1′). FTIR (thin film) *ν*_max_ 2918 (s), 2850 (s), 1957 (m), 1692 (s), 1615 (s), 1563 (s), 1469 (s), 1378 (m), 1331 (s), 1257 (m), 1191 (m), 1141 (s), 1106 (s), 996 (m), 851 (m), 753 (s), 423 (m) cm^−1^. UV-vis (CH_2_Cl_2_) λ_max_/nm (ε, M^−1^ cm^−1^) 235 (8560), 279 (6869), 542 (1706). LRMS-ESI (15%) *m*/*z* 367 [M + H]^+^; HRMS-ESI *m*/*z* for C_24_H_19_N_2_O_2_ calculated 367.1447 [M + H]^+^, found 367.1445.

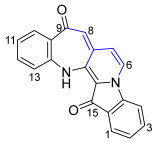

*9H-Benzo[6′,7′]azepino[2′,3′:3,4]pyrido[1,2-a]indole-9,15(14H)-dione* **21**

Compound **21** was isolated as a purple solid. R*_f_* (30% EtOAc/*n*-hexane) 0.30. mp. 168–170 °C. ^1^H NMR (400 MHz, CDCl_3_) δ 10.73 (1H, s, NH), 7.67 (1H, appt. ddd, *J* = 7.6, 1.4, 0.8 Hz, H10), 7.63 (1H, ddd, *J* = 7.7, 1.3, 0.7 Hz, H1), 7.47 (1H, dt, *J* = 7.7, 1.3 Hz, H3), 7.45 (1H, dt, *J* = 7.6, 1.4 Hz, H12), 7.17 (1H, td, *J* = 7.7, 0.7 Hz, H2), 7.12 (1H, dt, *J* = 7.7, 0.7 Hz, H4), 7.10 (1H, d, *J* = 2.8 Hz, H6), 7.10 (1H, dt, *J* = 7.6, 0.8 Hz, H13), 6.90 (1H, td, *J* = 7.6, 0.8 Hz, H11), 6.50 (1H, s, H8), 6.39 (1H, d, *J* = 2.8 Hz, H7). ^13^C NMR (101 MHz, CDCl_3_) δ 187.1 (C9), 180.3 (C15), 153.0 (C13a), 142.4 (C4a), 137.9 (C14a), 136.4 (C12), 134.6 (C3), 129.6 (C15a), 126.9 (C14b), 126.5 (C7a), 125.8 (C2), 125.0 (C1), 124.8 (C10), 121.4 (C6), 120.7 (C9a), 120.5 (C7), 120.1 (C11), 112.2 (C13), 110.5 (C4), 101.1 (C8). FTIR (thin film) *ν*_max_ 3360 (m), 2918 (s), 2850 (s), 1712 (s), 1610 (s), 1481 (s), 1468 (s), 1375 (m), 1312 (s), 1254 (s), 1201 (m), 1111 (s), 889 (m), 852 (m), 755 (s) cm^−1^. UV-vis (CH_2_Cl_2_) λ_max_/nm (ε, M^−1^ cm^−1^) 236 (8664), 264 (7116), 352 (1685), 561 (1922). LRMS-ESI (25%) *m*/*z* 313 [M + H]^+^; HRMS-ESI *m*/*z* for C_20_H_13_N_2_O_2_ calculated 313.0977 [M + H]^+^, found 313.0966.

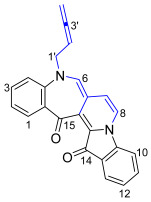

*5-(Buta-2,3-dien-1-yl)-5H-benzo[6′,7′]azepino[4′,3′:3,4]pyrido[1,2-a]indole-14,15-dione* **22**

Compound **22** was isolated as a red waxy solid. R*_f_* (30% EtOAc/*n*-hexane) 0.40. ^1^H NMR (400 MHz, CDCl_3_) δ 7.88 (1H, dd, *J* = 2.9, 0.7 Hz, H8), 7.71 (1H, ddd, *J* = 7.4, 1.3, 0.7 Hz, H1), 7.61 (1H, dd, *J* = 7.7, 1.3 Hz, H13), 7.49 (1H, ddd, *J* = 8.3, 7.4, 1.3 Hz, H3), 7.44 (1H, td, *J* = 7.7, 1.3 Hz, H11), 7.15 (1H, td, *J* = 7.7, 0.8 Hz, H12), 7.14 (1H, appt. ddd, *J* = 7.7, 1.3, 0.8 Hz, H10), 7.11 (1H, dd, *J* = 2.9, 0.7 Hz, H7), 7.00 (1H, s, H6), 6.94 (1H, appt. dd, *J* = 8.3, 0.7 Hz, H4), 6.91 (1H, appt. td, *J* = 7.4, 0.7 Hz, H2), 5.24 (1H, quint, *J* = 6.1 Hz, H2′), 4.93 (2H, dt, *J* = 6.1, 3.0 Hz, H4′), 4.49 (2H, dt, *J* = 6.1, 3.0 Hz, H1′). ^13^C NMR (101 MHz, CDCl_3_) δ 209.3 (C3′), 184.8 (C15), 178.7 (C14), 151.8 (C4a), 142.9 (C9a), 137.8 (C14a), 136.0 (C3), 133.8 (C11), 130.8 (C13a), 130.4 (C14b), 126.1 (C6a), 125.3 (C10), 124.8 (C1), 124.4 (C13), 121.3 (C15a), 119.8 (C7), 119.6 (C2), 117.9 (C8), 110.5 (C12), 109.1 (C4), 106.3 (C6), 85.0 (C2′), 77.9 (C4′), 41.4 (C1′). FTIR (thin film) *ν*_max_ 2959 (s), 2922 (s), 2853 (s), 1955 (m), 1676 (s), 1616 (s), 1476 (s), 1340 (s), 1260 (s), 1190 (s), 1096 (s), 1019 (s), 799 (s), 752 (m), 415 (m) cm^−1^. UV-vis (CH_2_Cl_2_) λ_max_/nm (ε, M^−1^ cm^−1^) 225 (16158), 260 (10332), 370 (5735), 543 (4369). LRMS-ESI (30%) *m*/*z* 397 [M + CH_3_OH + H]^+^; HRMS-ESI *m*/*z* for C_24_H_16_N_2_O_2_Na calculated 387.1109 [M + Na]^+^, found 387.1123.

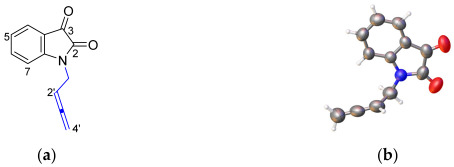

*1-(Buta-2,3-dien-1-yl)indoline-2,3-dione* **23**: (**a**) structure; (**b**) X-ray structure

Compound **23** was isolated as a yellow crystalline solid (needles). X-ray quality crystals were grown through slow crystallization from 20% CH_2_Cl_2_/*n*-hexane. R*_f_* (30% EtOAc/*n*-hexane) 0.38. ^1^H NMR (400 MHz, CDCl_3_) δ 7.61 (1H, ddd, *J* = 7.7, 1.4, 0.7 Hz, H4), 7.59 (1H, td, *J* = 7.7, 1.4 Hz, H6), 7.13 (1H, td, *J* = 7.7, 0.7 Hz, H5), 6.94 (1H, dt, *J* = 7.7, 0.7 Hz, H7), 5.20 (1H, quint, *J* = 6.3 Hz, H2′), 4.86 (2H, dt, *J* = 6.3, 3.0 Hz, H4′), 4.36 (2H, dt, *J* = 6.3, 3.0 Hz, H1′). ^13^C NMR (101 MHz, CDCl_3_) δ 209.3 (C3′), 183.3 (C3), 157.8 (C2), 150.7 (C7a), 138.3 (C6), 125.4 (C4), 123.8 (C5), 117.7 (C3a), 110.9 (C7), 84.8 (C2′), 78.2 (C4′), 38.7 (C1′). FTIR (thin film) *ν*_max_ 2919 (s), 2850 (m), 1959 (m), 1740 (s), 1613 (s), 1470 (s), 1349 (s), 1178 (m), 1096 (m), 854 (m), 755 (s), 474 (m) cm^−1^. UV-vis (CH_2_Cl_2_) λ_max_/nm (ε, M^−1^ cm^−1^) 242 (11812), 285 (1983). LRMS-ESI (100%) *m*/*z* 254 [M + CH_3_OH + Na]^+^; HRMS-ESI *m*/*z* for C_12_H_9_NO_2_Na calculated 222.0531 [M + Na]^+^, found 222.0526.

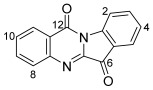

*Indolo[2,1-b]quinazoline-6,12-dione* **24** *(Tryptanthrin)*

Compound **24** was isolated as a yellow papery solid. R*_f_* (30% EtOAc/*n*-hexane) 0.37. mp. 258–260 °C. ^1^H NMR (400 MHz, CDCl_3_) δ 8.64 (1H, dt, *J* = 7.8, 0.8 Hz, H2), 8.44 (1H, dd, *J* = 8.1, 1.4 Hz, H11), 8.04 (1H, dd, *J* = 8.1, 1.4 Hz, H8), 7.92 (1H, ddd, *J* = 7.8, 1.4, 0.8 Hz, H5), 7.86 (1H, ddd, *J* = 8.1, 7.3, 1.4 Hz, H9), 7.79 (1H, td, *J* = 7.8, 1.4 Hz, H3), 7.68 (1H, ddd, *J* = 8.1, 7.3, 1.4 Hz, H10), 7.43 (1H, td, *J* = 7.8, 0.8 Hz, H4). ^13^C NMR (101 MHz, CDCl_3_) δ 182.6 (C6), 158.1 (C12), 146.7 (C7a), 146.4 (C1a), 144.4 (C6a), 138.3 (C3), 135.1 (C9), 130.8 (C8), 130.3 (C10), 127.6 (C11), 127.2 (C4), 125.4 (C5), 123.8 (11a), 122.0 (C5a), 118.0 (C2). FTIR (thin film) *ν*_max_ 2950 (s), 2923 (s), 2853 (s), 1734 (s), 1695 (s), 1596 (s), 1461 (s), 1354 (s), 1316 (s), 1261 (s), 1214 (m), 1184 (m), 1103 (s), 1080 (s), 1021 (s), 926 (m), 868 (m), 800 (s), 776 (m), 757 (s), 688 (m), 543 (m), 472 (m) cm^−1^. UV-vis (CH_2_Cl_2_) λ_max_/nm (ε, M^−1^ cm^−1^) 242 (18673), 275 (4130), 308 (4409), 328 (4081), 343 (3504), 392 (3429). LRMS-EI (100%) *m*/*z* 248 [M]^+^; HRMS-ESI *m*/*z* for C_15_H_9_N_2_O_2_ calculated 249.0664 [M + H]^+^, found 249.0669. The spectroscopic data were consistent with those reported [33,34].

### 3.4. Preparation of N-Methylindigo

*N*-Methylindigo **25** was synthesized as reported with some modifications on the base used [7,10]. A suspension of indigo **1** (0.66 g, 2.5 mmol, 1.0 eq.) in anhydrous DMF (40 mL/mmol) was sonicated for 60 min under a N_2_ atmosphere. The resulting suspension was transferred using a cannula to a flask containing pre-dried Cs_2_CO_3_ (3.26 g, 10 mmol, 4.0 eq.) and plunged into a pre-heated oil bath at 90 °C under N_2_ with stirring. After 90 min, iodomethane (1.77 g, 0.78 mL, 12.5 mmol, 5.0 eq.) was added, and the reaction mixture was stirred for 5 sec at 90 °C. The reaction was then quenched in an ice bath (400 mL/mmol) and filtered after the ice melted. The precipitate was washed with *n*-hexane (3 × 10 mL/mmol), dissolved in CH_2_Cl_2_ (60–100 mL/mmol), and concentrated in vacuo. The precipitate (420 mg) was subjected to purification using automated flash chromatography using 40% CH_2_Cl_2_/*n*-hexane → 100% CH_2_Cl_2_ (dry loading method with 13 × sample weight for silica absorbed and 40 g of silica flash cartridge) to furnish *N*-methylindigo **25** (0.37 g, 1.35 mmol, 54%).

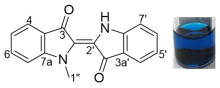



Compound **25** was isolated as a blue papery solid; the image shown of the solution of **25** in CH_2_Cl_2_ was taken under ambient lighting. R*_f_* (30% EtOAc/*n*-hexane) 0.68. mp. 252–254 °C. ^1^H NMR (400 MHz, CDCl_3_) δ 10.51 (1H, s, NH), 7.73 (1H, ddd, *J* = 7.4, 1.3, 0.8 Hz, H4), 7.68 (1H, appt. ddd, *J* = 7.4, 1.4, 0.8 Hz, H4′), 7.55 (1H, ddd, *J* = 8.3, 7.4, 1.3 Hz, H6), 7.46 (1H, ddd, *J* = 8.2, 7.4, 1.4 Hz, H6′), 7.07 (1H, d, *J* = 8.3 Hz, H7), 7.02 (1H, appt. td, *J* = 7.4, 0.8 Hz, H5), 7.00 (1H, dt, *J* = 8.2, 0.8 Hz, H7′), 6.95 (1H, appt. td, *J* = 7.4, 0.8 Hz, H5′), 3.88 (3H, s, C1″). ^13^C NMR (101 MHz, CDCl_3_) δ 189.9 (C3), 187.0 (C3′), 153.7 (C7a), 151.4 (C7a’), 136.0 (C6′), 135.9 (C6), 125.2 (C2′), 124.7 (C4′), 124.2 (C2), 124.0 (C4), 120.7 (C5 and C5′), 120.6 (C3a), 120.4 (C3a’), 111.9 (C7′), 110.5 (C7), 35.3 (C1″). FTIR (thin film) *ν*_max_ 3260 (m), 1682 (w), 1641 (s), 1611 (s), 1473 (s), 1393 (s), 1295 (m), 1066 (s), 1013 (s), 923 (m), 737 (s) cm^−1^. UV-vis (CH_2_Cl_2_) λ_max_/nm (ε, M^−1^ cm^−1^) 248 (17338), 292 (16208), 631 (10077). LRMS-ESI (78%) *m*/*z* 276 [M]^+^; HRMS-ESI *m*/*z* for C_17_H_13_N_2_O_2_ calculated 277.0977 [M + H]^+^, found 277.0966. The spectroscopic data were consistent with those reported in [7,35].

### 3.5. General Procedure for the Reaction of N-Methylindigo with Buta-2,3-dien-1-yl Methanesulfonate

A solution of *N*-methylindigo **25** (1.0 eq.) in anhydrous DMF or THF (40 mL/mmol) was sonicated for 5 min under a N_2_ atmosphere. The resulting solution was transferred using a cannula to a flask containing pre-dried Cs_2_CO_3_ (1.0–2.0 eq.) or KHMDS (0.1 M in THF, 2.0 eq) and plunged into a pre-heated oil bath at 85–87 °C for anhydrous DMF or 65–68 °C for anhydrous THF under N_2_ with stirring. After 30–60 min, buta-2,3-dien-1-yl methanesulfonate **17** (3.0–5.0 eq.) was added, and the reaction mixture was stirred for 7 min to 24 h at 65–87 °C with the addition of electrophile **17** during the reaction as stated in Table 2. The reaction was then quenched in an ice bath (400 mL/mmol) and filtered after the ice melted. The filtrate was extracted with EtOAc (3 × 60–100 mL/mmol) and washed with brine (3 × 60–100 mL/mmol) and distilled water (5 × 60–100 mL/mmol). The organic layer was collected, dried (Na_2_SO_4_), and concentrated in vacuo. In addition, the precipitate was washed with *n*-hexane (3 × 10 mL/mmol), dissolved in EtOAc (60–100 mL/mmol), and concentrated in vacuo. In a separate separation and purification, either the filtrate or the precipitate was subjected to column chromatography using 20% *n*-hexane/DCM → 20% EtOAc/DCM or 20% → 50% EtOAc/n-hexane (dry loading method with 20 × sample weight for silica absorbed and 100–200 × sample weight for silica column) to yield minor fraction (F_1_), two major fractions, i.e., F_2_ (unreacted N-methylindigo **25**) and F_3_ (pink fraction), and baseline decomposition material. Further partial purification by multiple rounds of PTLC (40% EtOAc/n-hexane and 1% MeOH/CH_2_Cl_2_) gave cascade product **26** (crude), with a total of *<*5 mg, precluding further purification. Note that for entry 6 (Table 2), buta-2,3-dien-1-yl methanesulfonate **17** (1 eq.) was treated with KI (1 eq.) for 1 h at room temperature under an N_2_ atmosphere to generate buta-2,3-dien-1-yl iodide in situ. The iodide electrophile was then used directly without further purification.

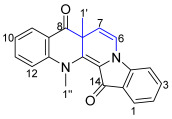

*7a,13-Dimethylbenzo[b]indolo[1,2-h][1,7]naphthyridine-8,14(7aH,13H)-dione* **26**

Compound **26** was isolated as a red waxy solid. R*_f_* (40% EtOAc/*n*-hexane) 0.63. ^1^H NMR (500 MHz, CDCl_3_) δ 7.93 (1H, dd, *J* = 7.8, 1.7 Hz, H9), 7.75 (1H, ddd, *J* = 7.8, 1.4, 0.8 Hz, H1), 7.60 (1H, ddd, *J* = 8.4, 7.2, 1.7 Hz, H11), 7.50 (1H, ddd, *J* = 8.4, 7.2, 1.4 Hz, H3), 7.13 (1H, dt, *J* = 8.4, 0.9 Hz, H12), 7.11 (1H, dd, *J* = 8.4, 0.8 Hz, H4), 7.06 (1H, ddd, *J* = 7.8, 7.2, 0.9 Hz, H10), 7.00 (1H, ddd, *J* = 7.8, 7.2, 0.8 Hz, H2), 6.87 (1H, d, *J* = 7.6 Hz, H6), 5.52 (1H, d, *J* = 7.6 Hz, H7), 3.72 (3H, s, H1″), 1.53 (3H, s, H1′). ^13^C NMR–insufficient material available. FTIR (thin film) *ν*_max_ 2924 (s), 2854 (s), 1690 (s), 1602 (s), 1561 (s), 1473 (s), 1350 (m), 1331 (s), 1260 (m), 1198 (m), 1148 (s), 1101 (s), 997 (m), 905 (m), 753 (s) cm^−1^. UV-vis (CH_2_Cl_2_) λ_max_/nm (ε, M^−1^ cm^−1^) 231 (12315), 275 (7214), 519 (2181). LRMS-ESI (36%) *m*/*z* 329 [M + H]^+^; HRMS-ESI *m*/*z* for C_21_H_17_N_2_O_2_ calculated 329.1290 [M + H]^+^, found 329.1293.

## 4. Conclusions

A new synthetic route has been established to afford *N*-heterocyclic scaffolds from the readily available and cheap indigo **1** dye. These reactions reveal that the reactivity of indigo **1** could be raised once its N-H proton is deprotonated. The reactive indigo **1** anion can undergo reaction with buta-2,3-dien-1-yl methanesulfonate **17**, allowing access to the novel, complex, and diverse *N*-heterocycles. Here, we identified the new ring expansion products (**20**–**22**) via a multi-step, one-pot, and domino-type series of indigo **1** reactions with an allene-based electrophile **17**, with two new heterocyclic scaffolds arising from intriguing new cascade pathways.

These investigations have provided valuable insights into the chemistry of indigo **1,** and using buta-2,3-dien-1-yl mesylate **17** as an electrophile further extended the reaction possibilities. Multiple reactions take place, however, with poor yields of the cascade products. The difference between using an allenylic moiety as an electrophile and allenics as cascade intermediates from alkynes is that there are more defined reaction pathway options in allenic intermediates, resulting in moderate to high yields of cascade products. Despite the modest yields, the buta-2,3-dien-1-ylation of indigo **1** contributes to the new science of indigo **1**, including the boundaries of reactivity. Further, this work contributes to the understanding of the unifying mechanistic pathways of indigo cascade reactions, with proposals presented in this work based on prior studies reported in the literature under analogous conditions [5,8,9,10]. However, due to the complexity of the reaction mixture and the rapid formation of multiple products, as indicated by TLC analysis, detailed mechanistic investigations are beyond the scope of this work. Possible intermediate isolations, isotopic labelling experiments, and computational studies are planned as part of future investigations to further underpin the mechanistic rationale. Future work will also include investigating photophysical properties and biological activities, e.g., antibacterial and antiplasmodial, of the synthesized compounds. Extensions to the directions in synthetic chemistry include exploring the cascade reactions of indigo **1** analogs and investigating electron-withdrawing groups in the allenylic unit to moderate reactivity.

## Figures and Tables

**Table 1:** The addition of buta-2,3-dien-1-yl methanesulfonate **17** to indigo **1** and optimization attempts. All images are CH_2_Cl_2_ solutions taken under ambient lighting or with 365 nm UV irradiation.

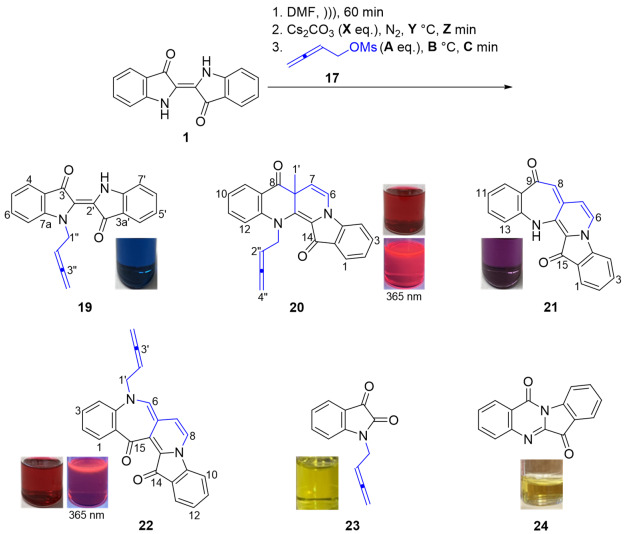												
Entry	Reaction Conditions						Compounds Yield (%)					
	X eq.	Y °C	Z min	A eq.	B °C	C min	19	20	21	22	23	24
1	2	85–87	60	5	85–87	7	16	3	6	<1	–	–
2	2	85–87	60	5	85–87	1	2	–	–	–	24	15
3	4	85–87	60	5	85–87	1	1	–	–	–	–	4
4	4	85–87	60	5	85–87	7	16	4	6	<1	<1	–
5 ^a^	4	90	60	8	85–87	15	2	3	6	3	2	4
6	4	90	60	5	70	55	<1	–	4	–	–	–
7	4	90	90	5	75	10	4	9	5	–	–	–
8	4	90	90	5	75	7	9	3	3	–	–	–
9	4	90	90	5	80	7	6	3	8	–	–	–
10 ^b^	1.2	85	30	2.4	85	7	8	1	4	–	–	–

^a^ 5 eq. of electrophile **17** at 0 min, followed by adding 3 × 1 eq. at 4, 8, and 12 min. ^b^ Different work-up procedures; unreacted indigo **1** (48%).

**Table 2:** The buta-2,3-dien-1-ylation of *N*-methylindigo **25** and efforts at optimization. The image of the solution in CH_2_Cl_2_ was taken under ambient lighting or with 365 nm UV irradiation.

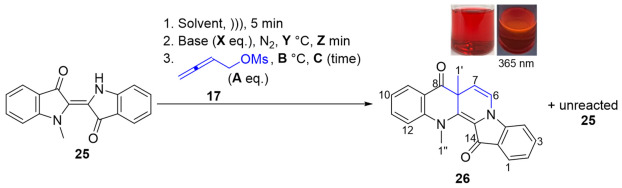										
Entry	Reactions Conditions								Compound Yield (%)	
	Solvent	Base	X eq.	Y °C	Z min	A eq.	B °C	C	25	26 *
1	DMF	Cs_2_CO_3_	2	85–87	30	3	85–87	7 min	58	8
2 ^a^	DMF	Cs_2_CO_3_	2	85–87	30	5	75–80	24 h	41	4
3 ^b^	DMF	Cs_2_CO_3_	1	85–87	60	7	85–87	75 min	24	3
4 ^c^	DMF	Cs_2_CO_3_	1	85–87	60	7	85–87	90 min	NP	NP
5 ^d^	THF	Cs_2_CO_3_	1	65–68	60	8	65–68	21 h	NP	NP
6 ^e^	DMF	Cs_2_CO_3_	2	85–87	30	6	85–87	40 min	NP	NP
7 ^f^	THF	KHMDS	2	65–68	30	6	65–68	40 min	NP	NP

* Reported as crude yield. ^a^ 3 eq. of electrophile **17** at 0 h, followed by adding 1 × 2 eq. at 3 h. ^b^ 5 eq. of electrophile **17** at 0 min, followed by adding 2 × 1 eq. at 30 and 60 min. ^c^ 5 eq. of electrophile **17** at 0 min, followed by adding 2 × 1 eq. at 5 and 10 min. ^d^ 5 eq. of electrophile **17** at 0 min, followed by adding 3 × 1 eq. at 5, 10 min, and 20 h. ^e^ Additional KI to the reaction; 3 eq. of electrophile **17** at 0 min, followed by adding 3 × 1 eq. at 10, 15, and 25 min. ^f^ KHMDS (0.1 M in THF); 3 eq. of electrophile **17** at 0 min, followed by adding 3 × 1 eq. at 5, 10, and 20 min. NP: Reaction mixture was not purified due to excessive unreacted *N*-methylindigo **25** and decomposition products.

## Data Availability

The original contributions presented in the study are included in the article or Supplementary Materials. Further inquiries can be directed to the corresponding author.

## Supplementary Materials

The following supporting information can be downloaded at https://www.mdpi.com/article/10.3390/molecules30142899/s1, reports all NMR data for synthesized compounds, the X-ray crystallographic data for compound **23**, and HRMS data for all new compounds.
