# Supplementary material for: Cascade Reactions of Indigo with an Allenylic Reactant
Source: Molecules. 2025 Jul 8;30(14):2899. doi: 10.3390/molecules30142899 (PMC12299176; doi:10.3390/molecules30142899)
Supplement: Supplementary file 1 [file molecules-30-02899-s001.zip › molecules-3712549-supplementary.pdf]

SUPPORTING INFORMATION

Cascade Reactions of Indigo with an Allenylic Reactant

Dyah U. C. Rahayu [1], Christopher Richardson [1], John B. Bremner [1] and Paul A. Keller [1,*]

[1] School of Science, Molecular Horizons, University of Wollongong, Wollongong, NSW 2522, Australia

Table of Contents

NMR Spectra for Buta-2,3-dien-1-ol (**16**) (Figures S1 to S2) S2

NMR Spectra for Buta-2,3-dien-1-yl Methanesulfonate (**17**) (Figures S3 to S4) S3

NMR Spectra for Buta-1,3-dien-2-yl Methanesulfonate (**18**) (Figures S5 to S10) S4

NMR Spectra for (*E*)-1-(Buta-2,3-dien-1-yl)-[2,2'-biindolinylidene]-3,3'-dione (**19**) (Figures S11 to S16) S7

NMR Spectra for 13-(Buta-2,3-dien-1-yl)-7a-methylbenzo[*b*]indolo[1,2-*h*][1,7]naphthyridine-8,14(7a*H*,13*H*)-dione (**20**) (Figures S17 to S22) S10

NMR Spectra for 9*H*-Benzo[6',7']azepino[2',3':3,4]pyrido[1,2-*a*]indole-9,15(14*H*)-dione (**21**) (Figures S23 to S28) S13

NMR Spectra for 5-(Buta-2,3-dien-1-yl)-5*H*-benzo[6',7']azepino[4',3':3,4]pyrido[1,2-*a*]indole-14,15-dione (**22**) (Figures S29 to S34) S16

NMR Spectra for 1-(Buta-2,3-dien-1-yl)indoline-2,3-dione (**23**) (Figures S35 to S40) S19

NMR Spectra for Indolo[2,1-*b*]quinazoline-6,12-dione (**24**) (Figures S41 to S42) S22

NMR Spectra for (*E*)-1-Methyl-[2,2'-biindolinylidene]-3,3'-dione (**25**) (Figures S43 to S44) S23

NMR Spectra for 7a,13-Dimethylbenzo[*b*]indolo[1,2-*h*][1,7]naphthyridine-8,14(7a*H*,13*H*)-dione (**26**) (Figures S45 to S47) S24

X-Ray Crystallography Data for 1-(Buta-2,3-dien-1-yl)indoline-2,3-dione (**23**) (Tables S1 to S7) S26

Mass Spectra for New Compounds (**18**, **19**, **20**, **21**, **22**, **23**, and **26**) (Figures S48 to S54)... S30

NMR Spectra for Buta-2,3-dien-1-ol (16)

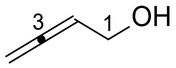

Compound **16**

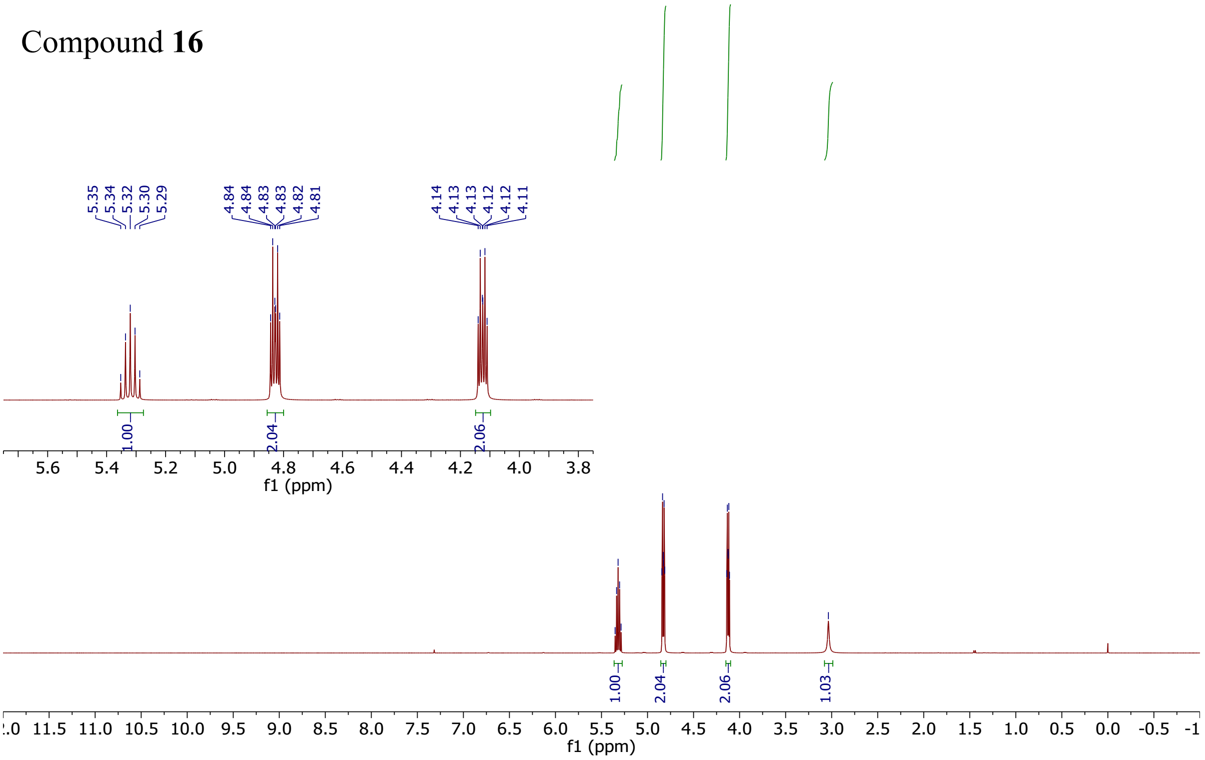

Figure S1. ^{1}H NMR spectrum of **16** (400 MHz, $CDCl_3$)

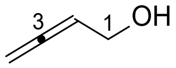

Compound **16**

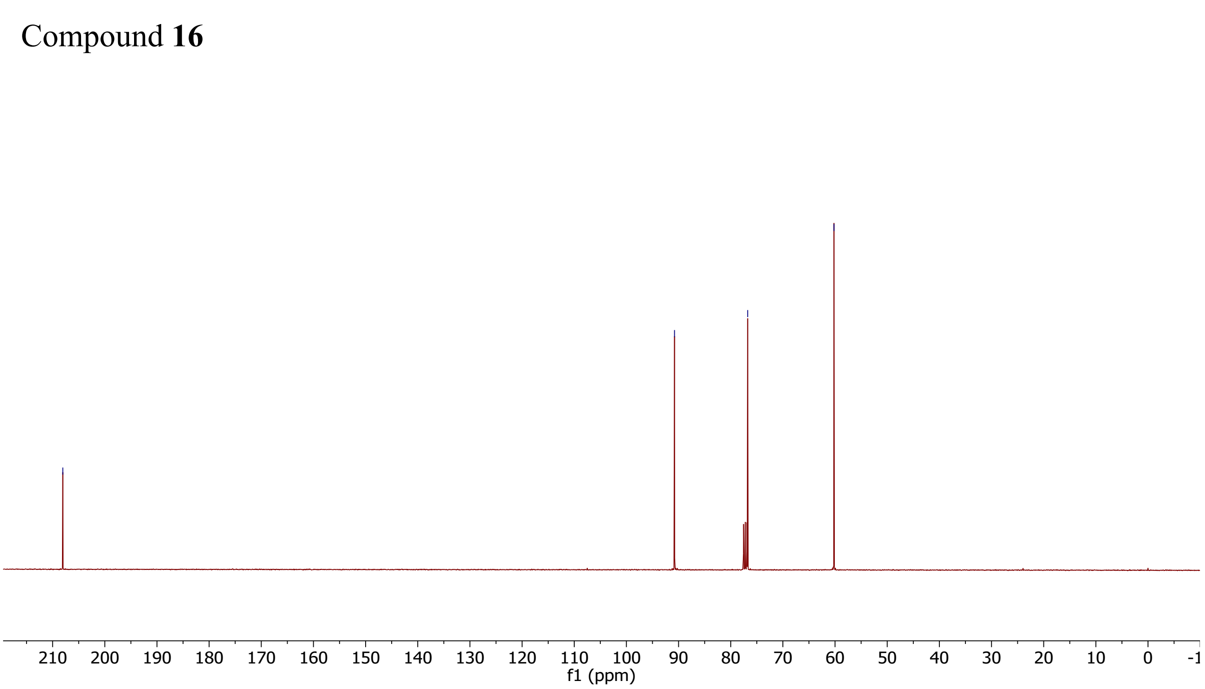

Figure S2. ^{13}C NMR spectrum of **16** (100 MHz, $CDCl_3$)

NMR Spectra for Buta-2,3-dien-1-yl Methanesulfonate (17)

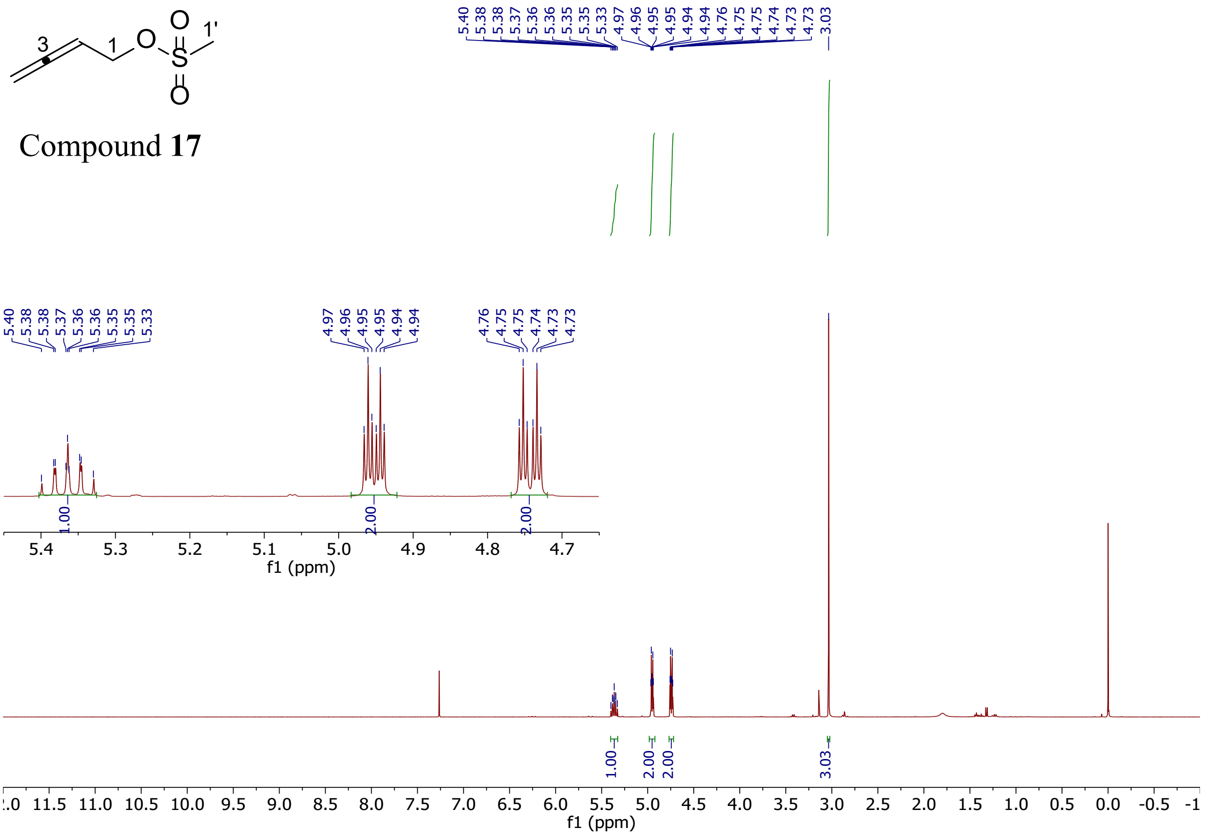

Figure S3. ^{1}H NMR spectrum of **17** (400 MHz, $CDCl_3$)

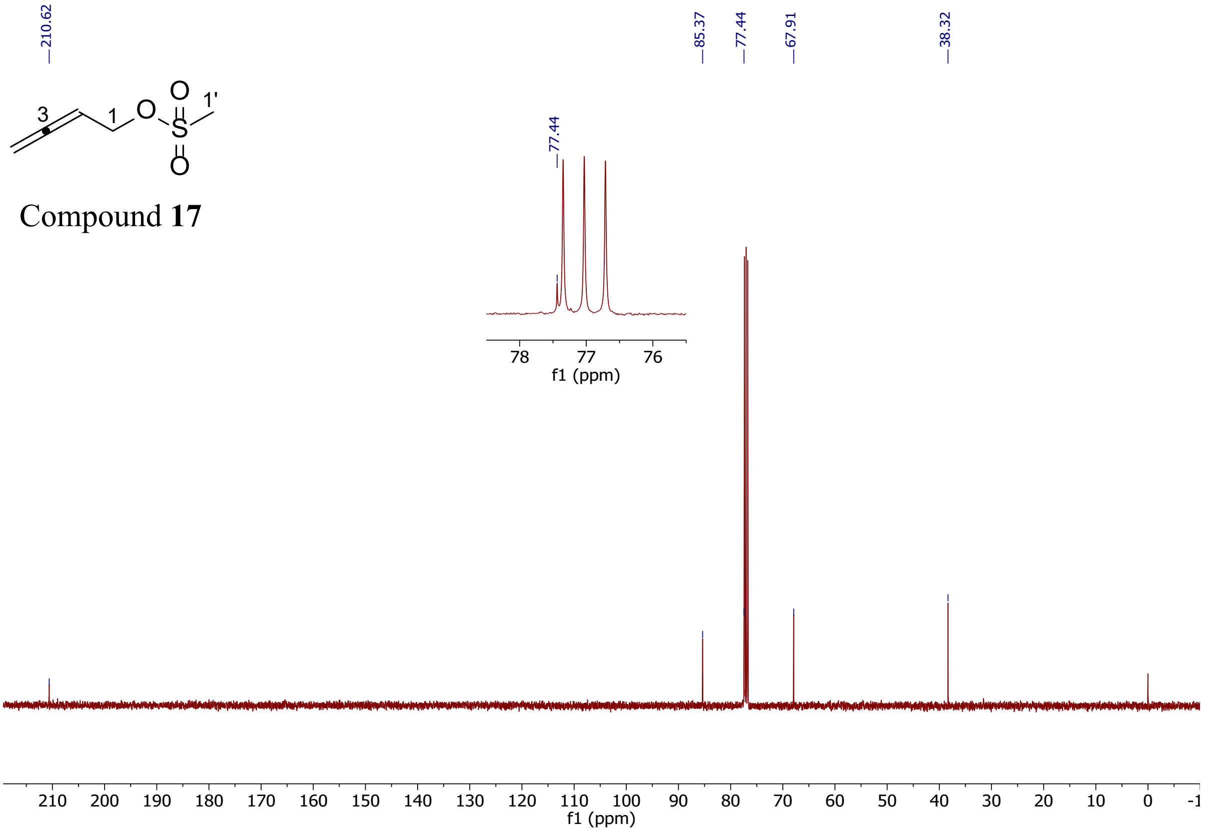

Figure S4. ^{13}C NMR spectrum of **17** (100 MHz, $CDCl_3$)

NMR Spectra for Buta-1,3-dien-2-yl Methanesulfonate (18)

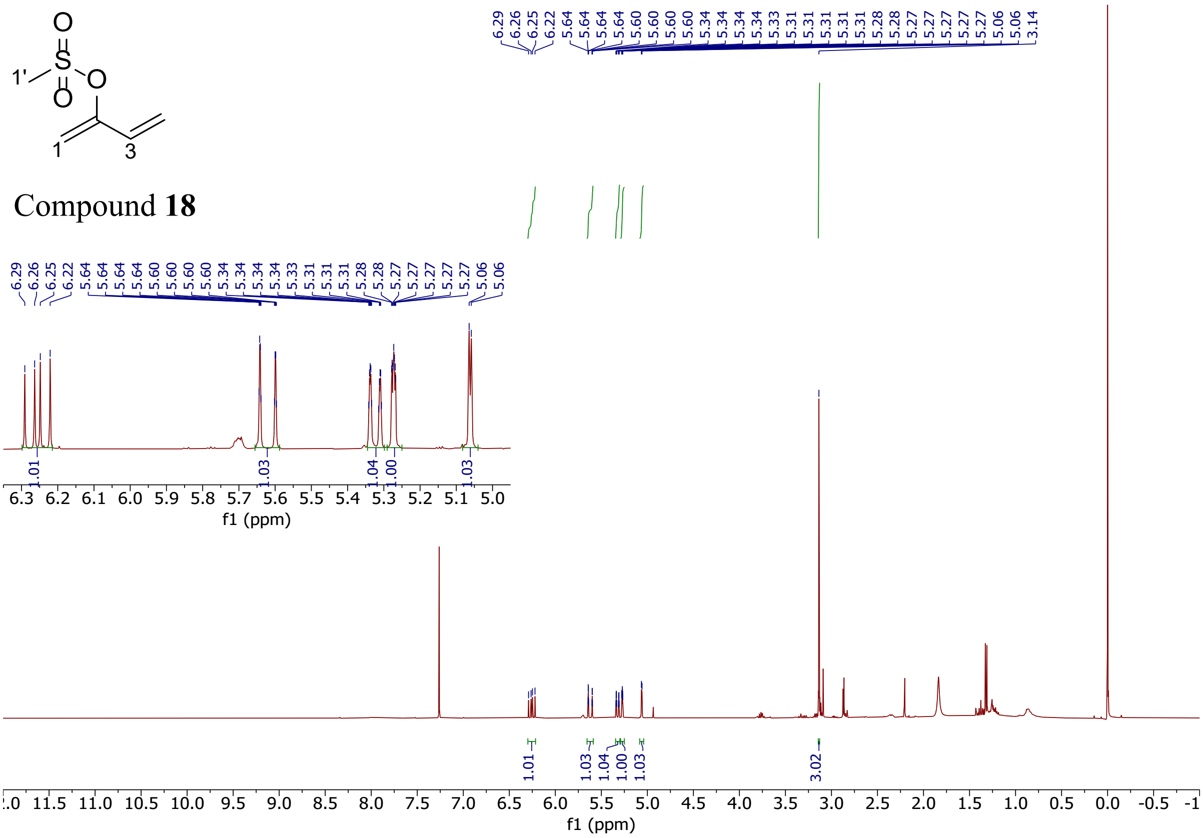

Figure S5. ^{1}H NMR spectrum of **18** (400 MHz, $CDCl_3$) with some grease/impurities

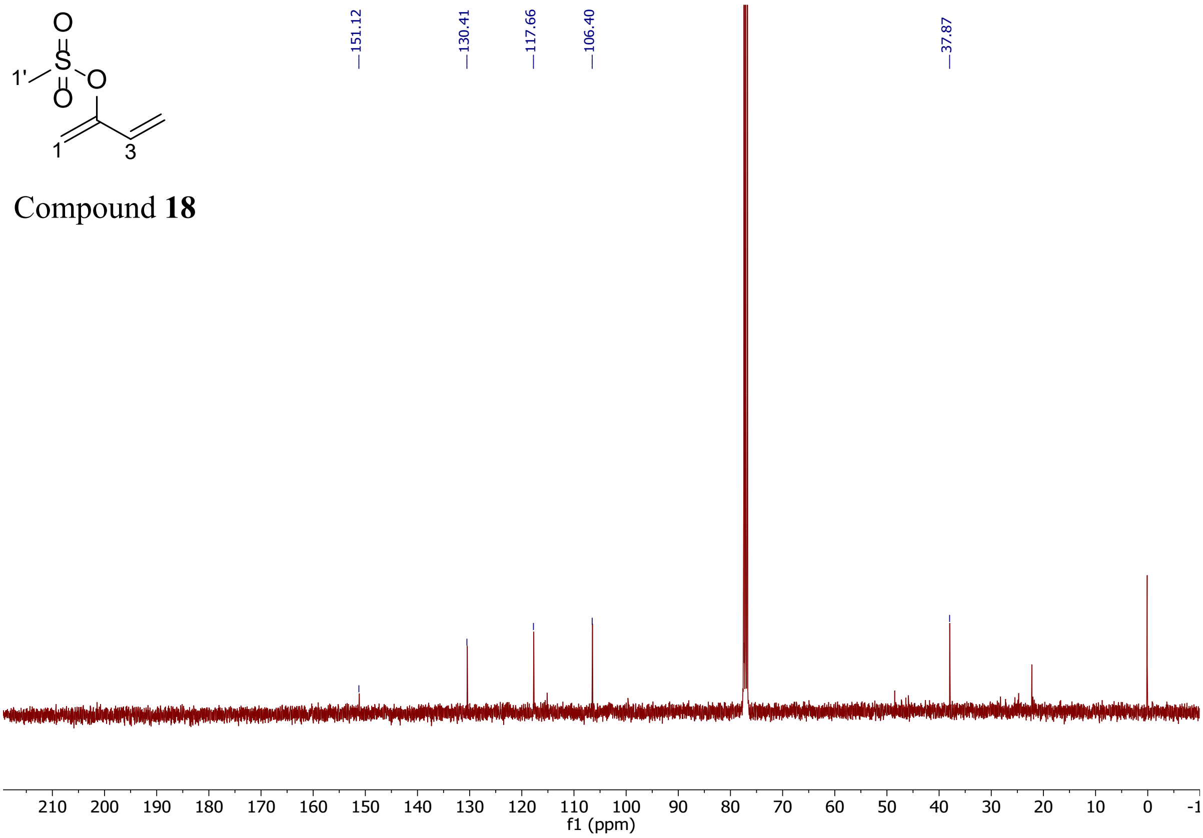

Figure S6. ^{13}C NMR spectrum of **18** (100 MHz, $CDCl_3$) with some grease/impurities

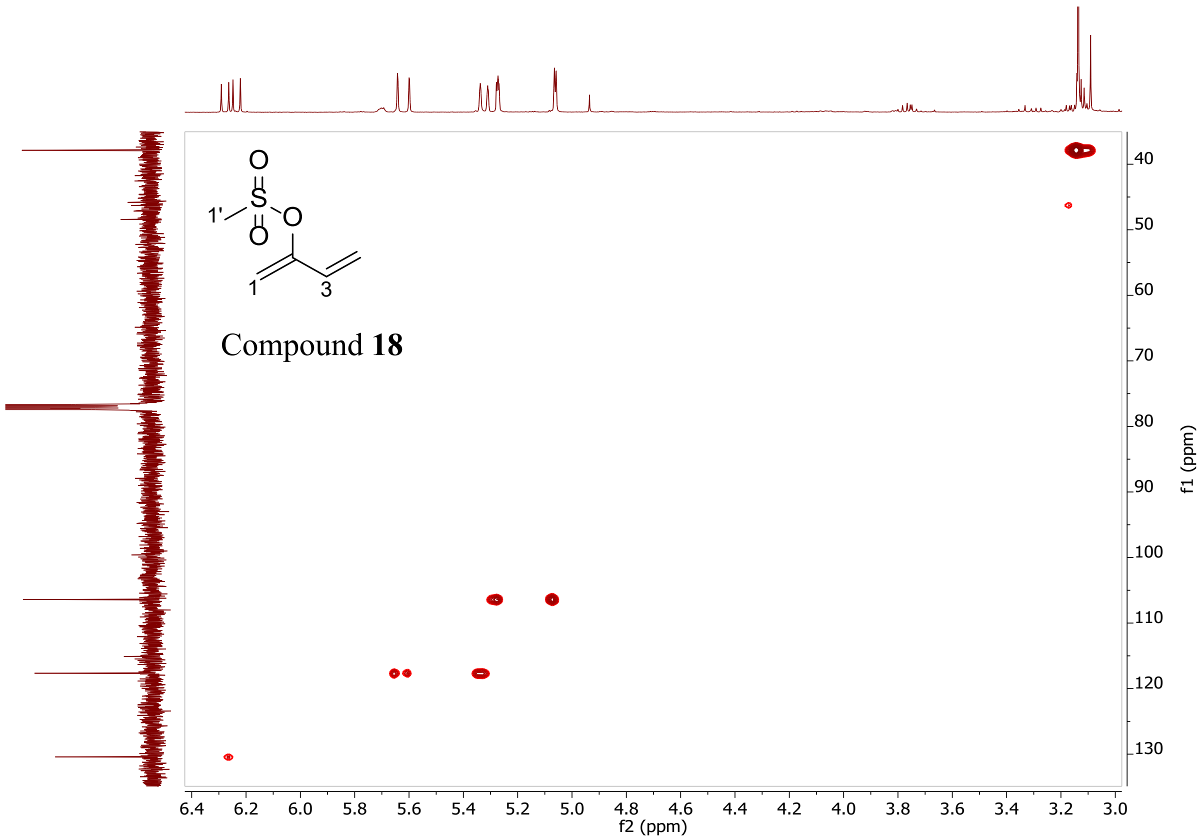

Figure S7. HSQC spectrum of **18** ($CDCl_3$)

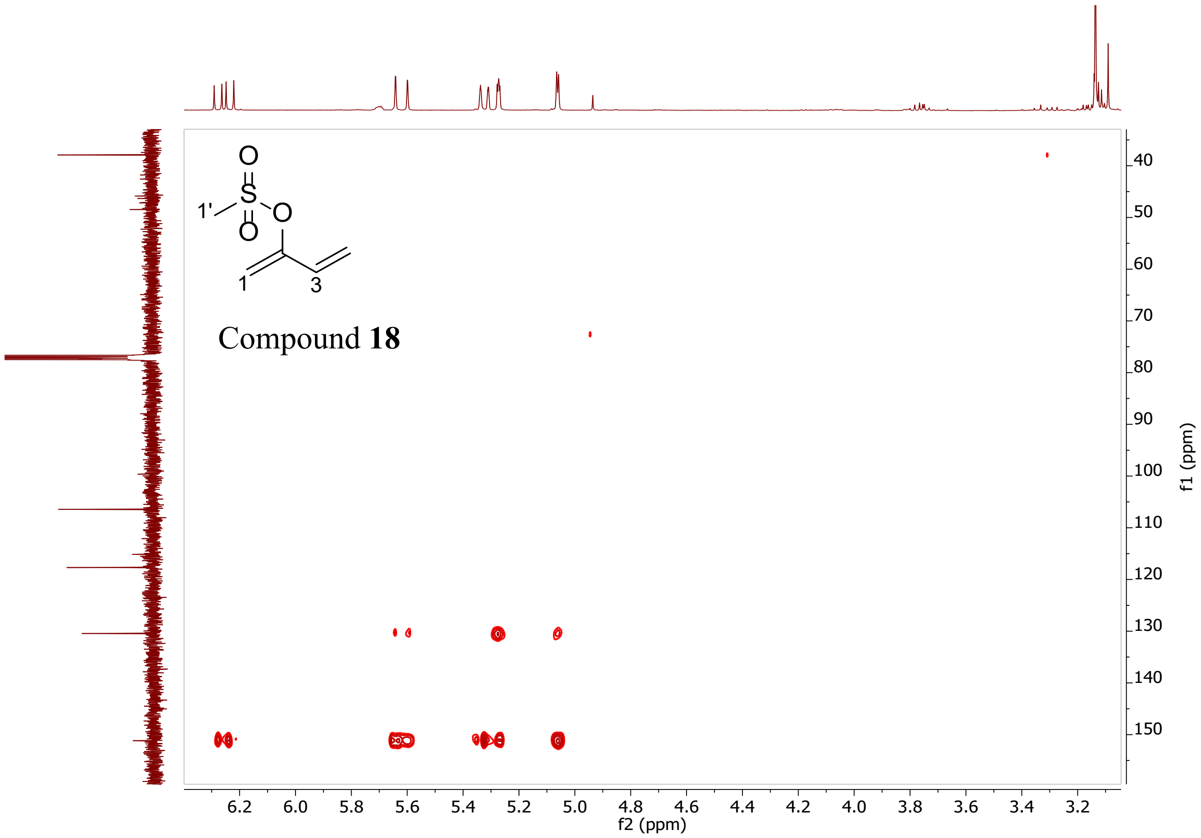

Figure S8. HMBC spectrum of **18** ($CDCl_3$)

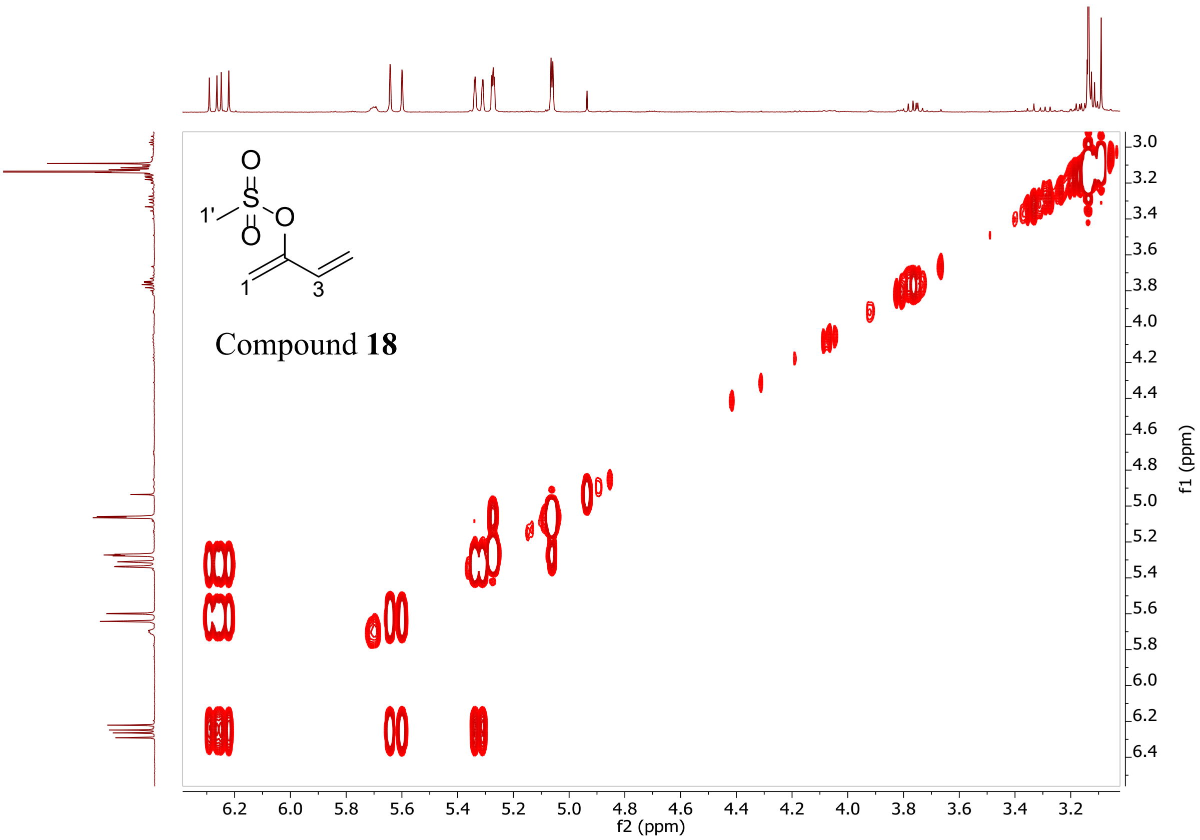

Figure S9. COSY spectrum of **18** ($CDCl_3$)

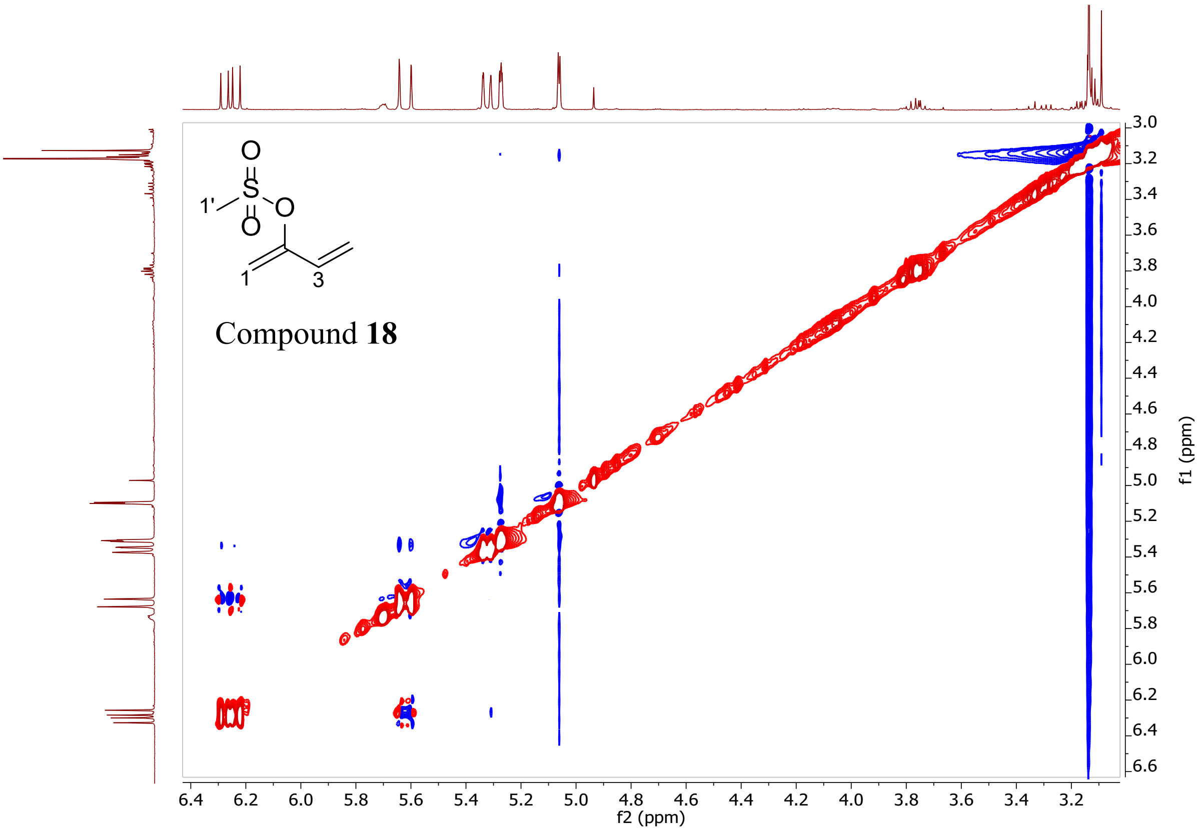

Figure S10. NOESY spectrum of **18** ($CDCl_3$)

NMR Spectra for (*E*)-1-(Buta-2,3-dien-1-yl)-[2,2'-biindolinylidene]-3,3'-dione (19)

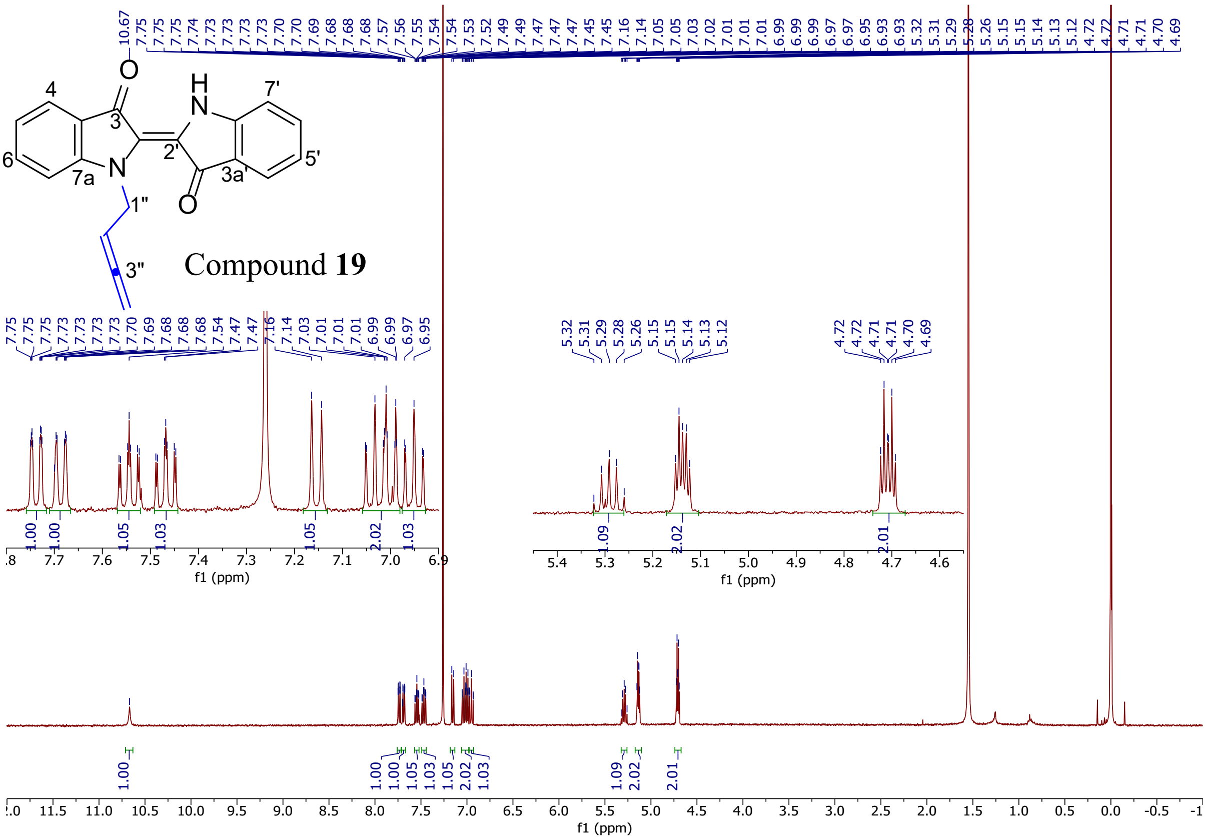

Figure S11. ^{1}H NMR spectrum of **19** (400 MHz, $CDCl_3$)

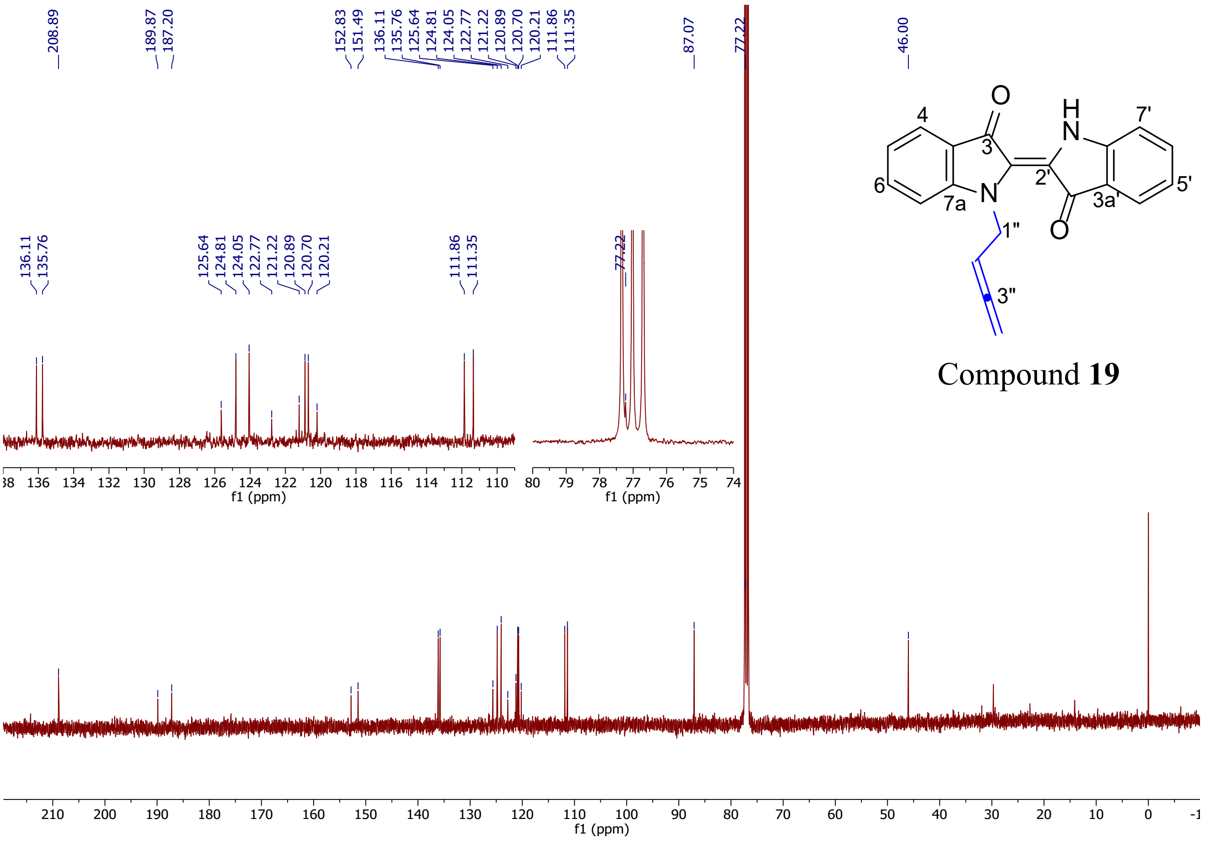

Figure S12. ^{13}C NMR spectrum of **19** (100 MHz, $CDCl_3$)

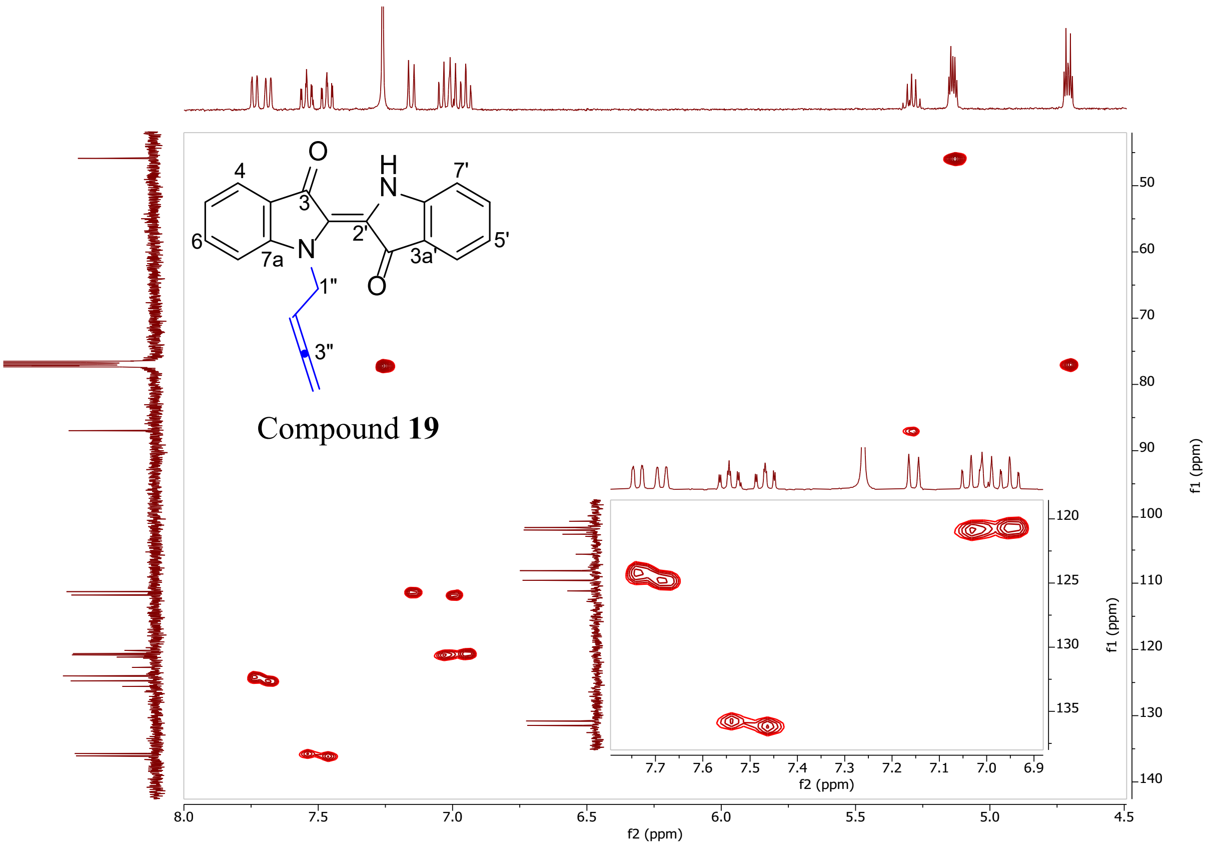

Figure S13. HSQC spectrum of **19** ($CDCl_3$)

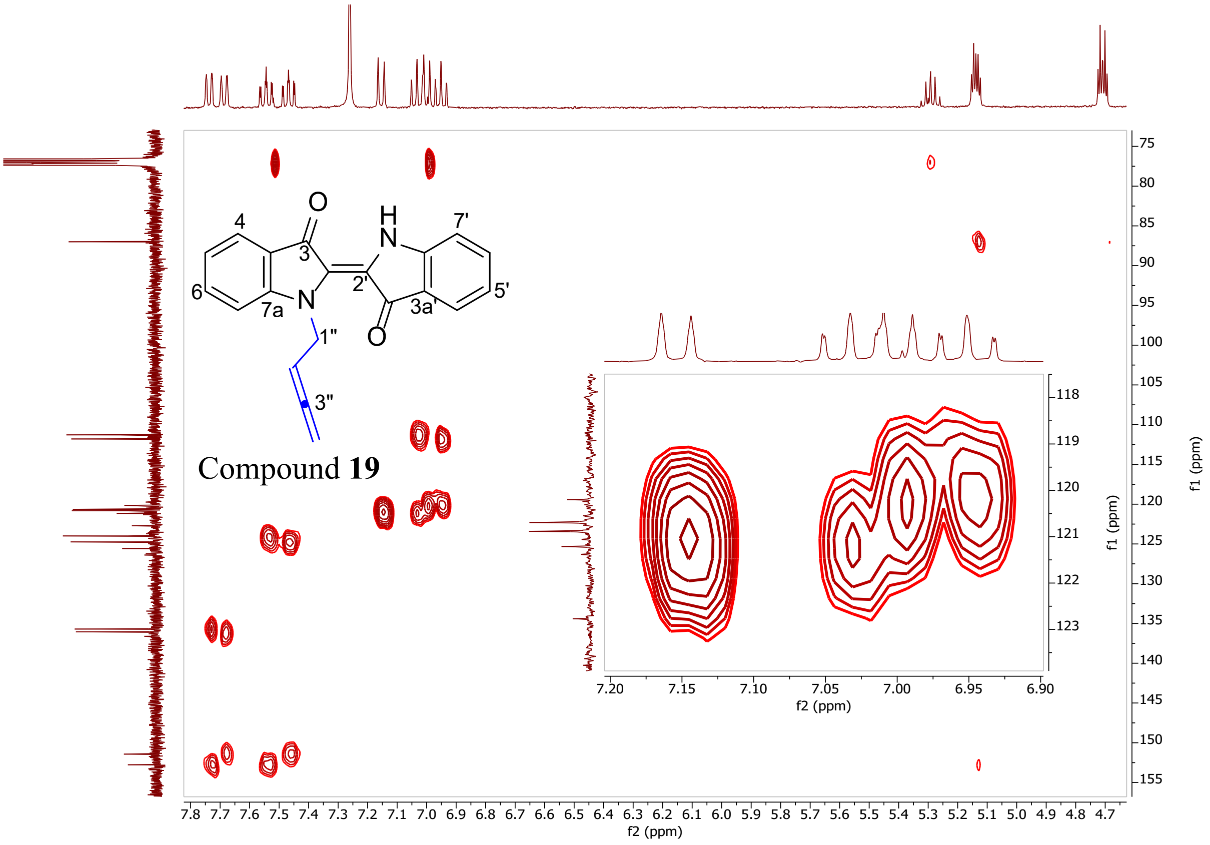

Figure S14. HMBC spectrum of **19** ($CDCl_3$)

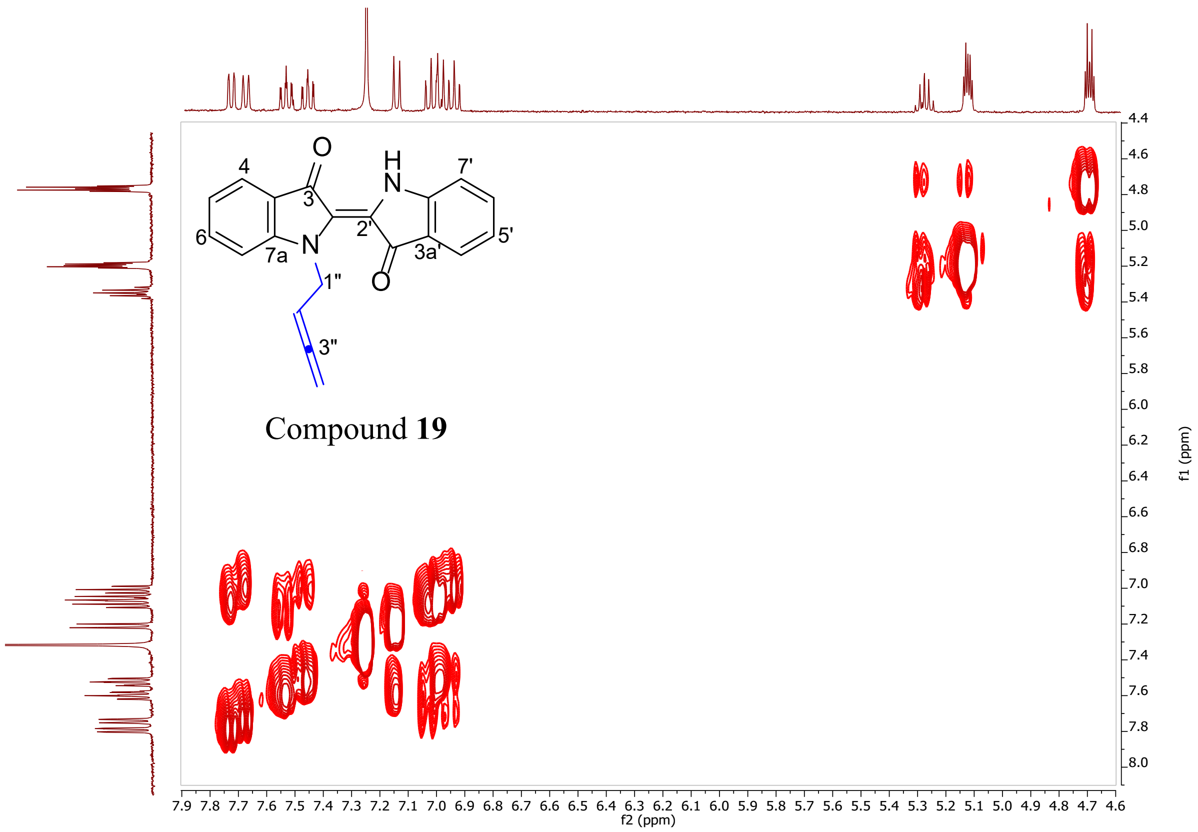

Figure S15. COSY spectrum of **19** ($CDCl_3$)

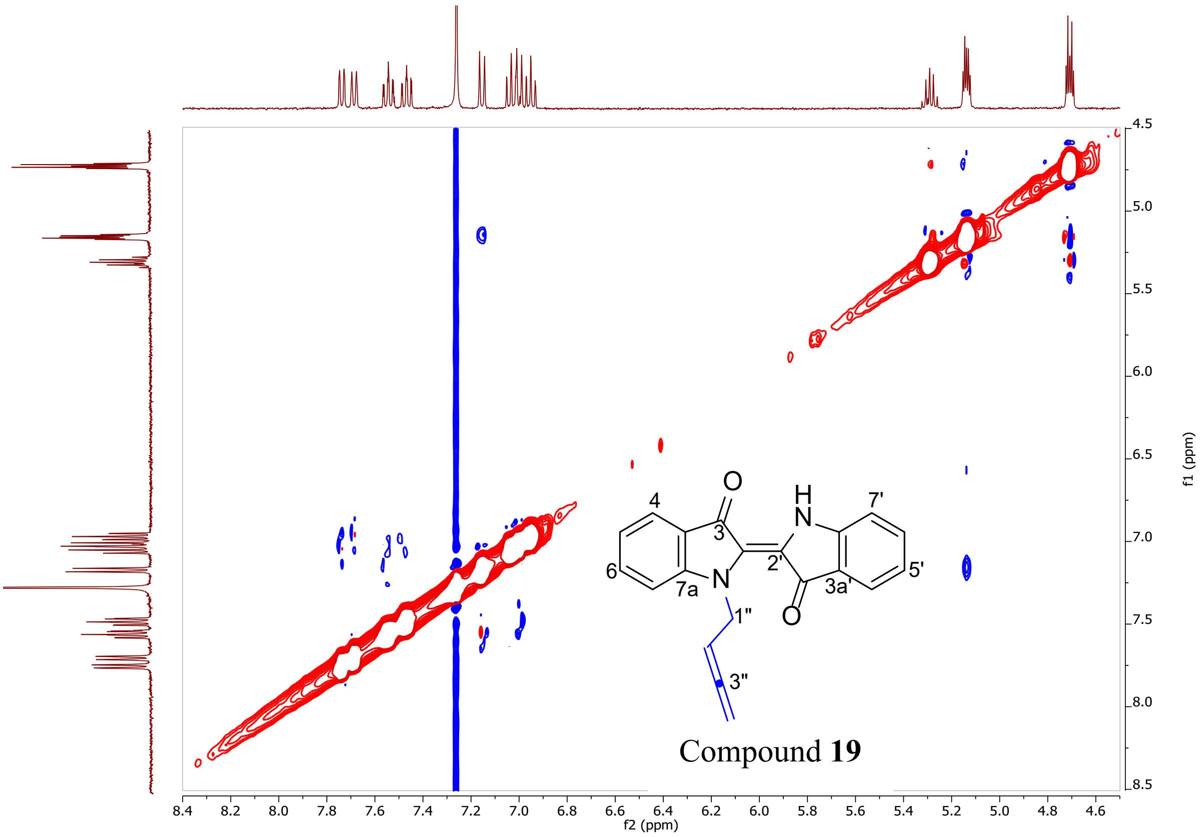

Figure S16. NOESY spectrum of **19** ($CDCl_3$)

NMR Spectra for 13-(Buta-2,3-dien-1-yl)-7a-methylbenzo[*b*]indolo[1,2-*h*][1,7]naphthyridine-8,14(7a*H*,13*H*)-dione (20)

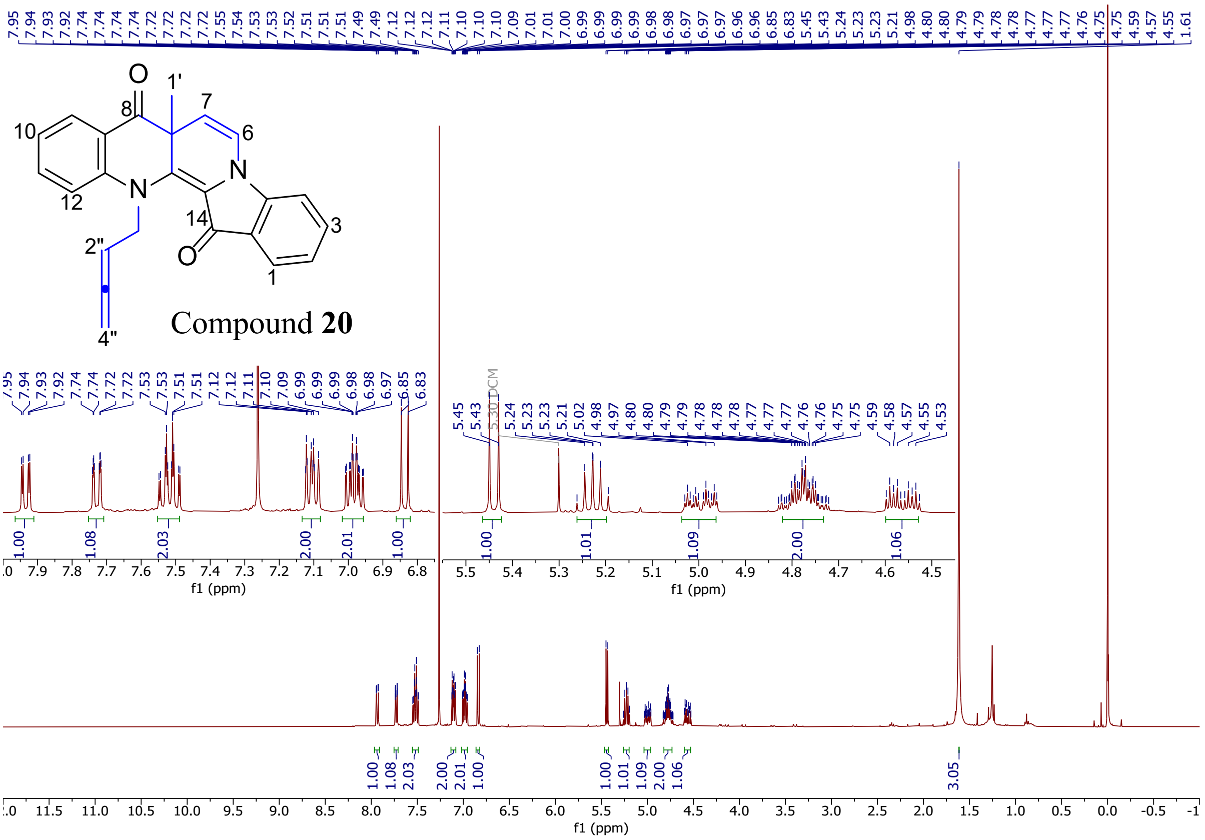

Figure S17. ^{1}H NMR spectrum of **20** (400 MHz, $CDCl_3$)

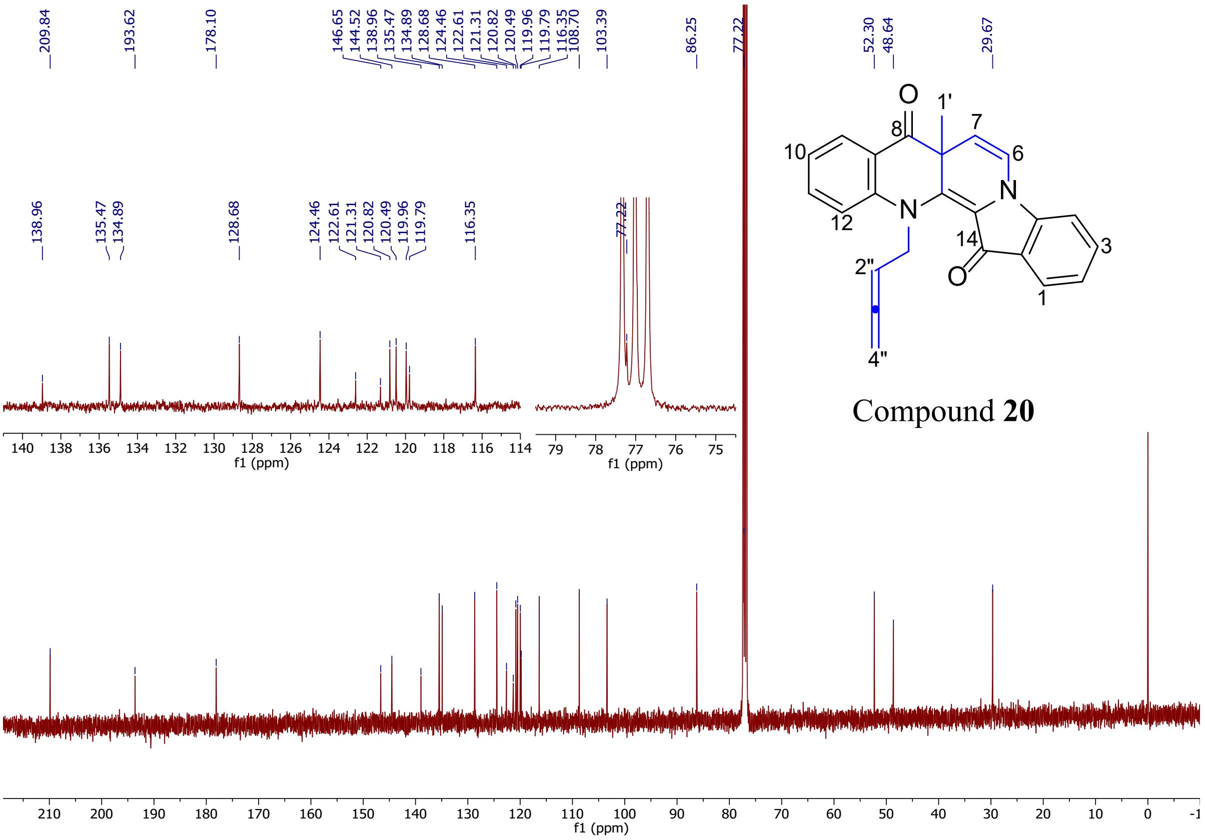

Figure S18. ^{13}C NMR spectrum of **20** (100 MHz, $CDCl_3$)

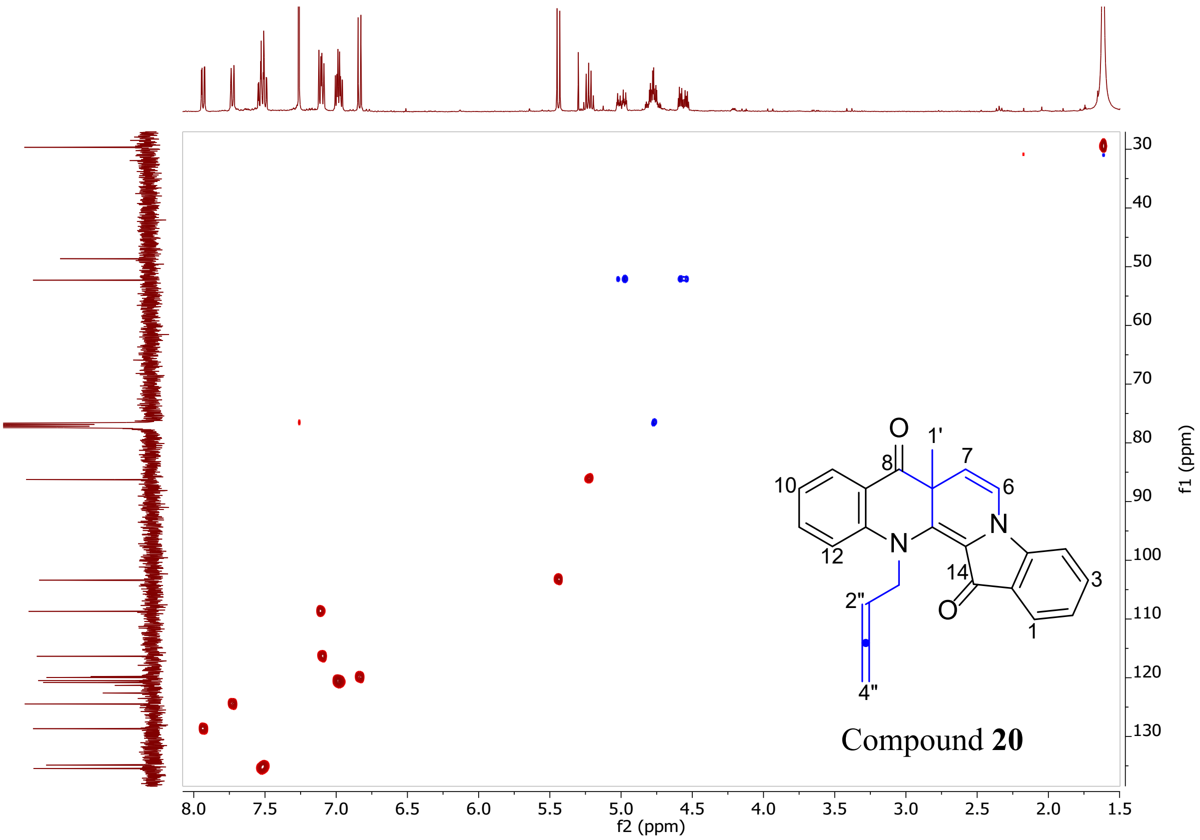

Figure S19. HSQC spectrum of **20** ($CDCl_3$)

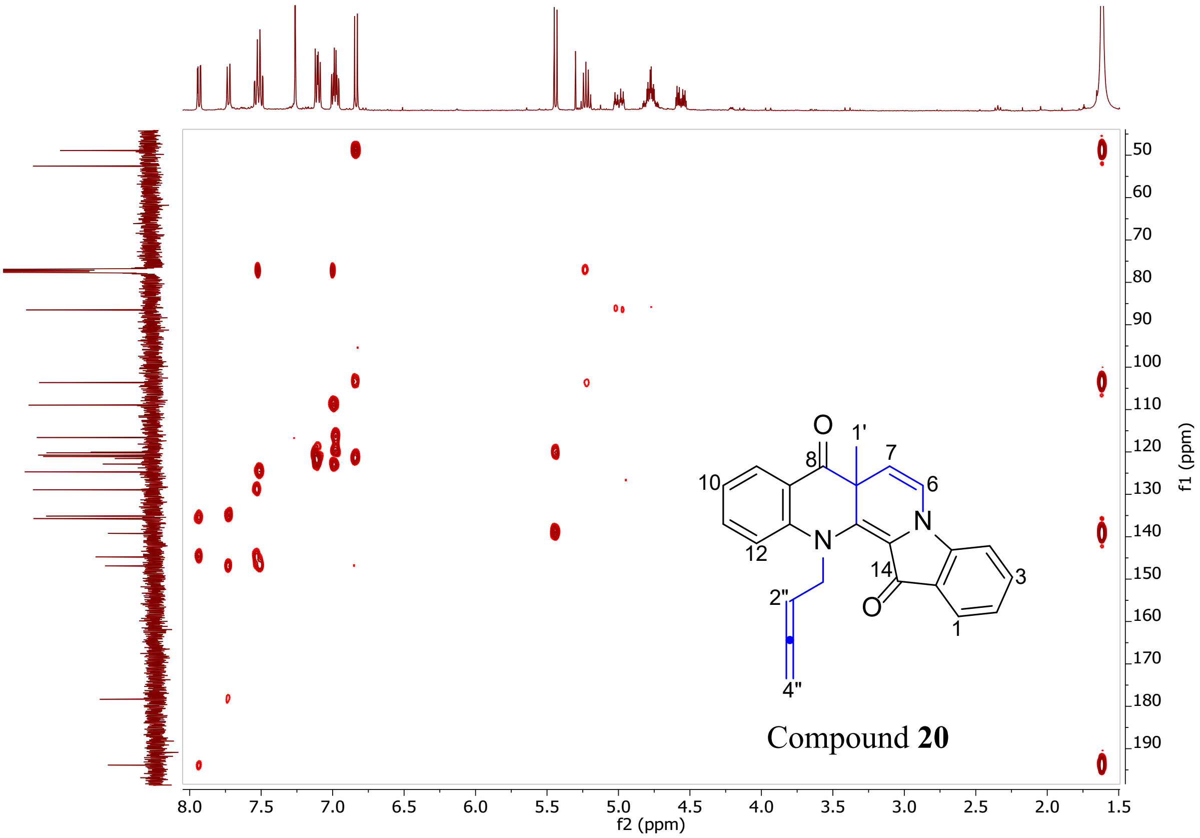

Figure S20. HMBC spectrum of **20** ($CDCl_3$)

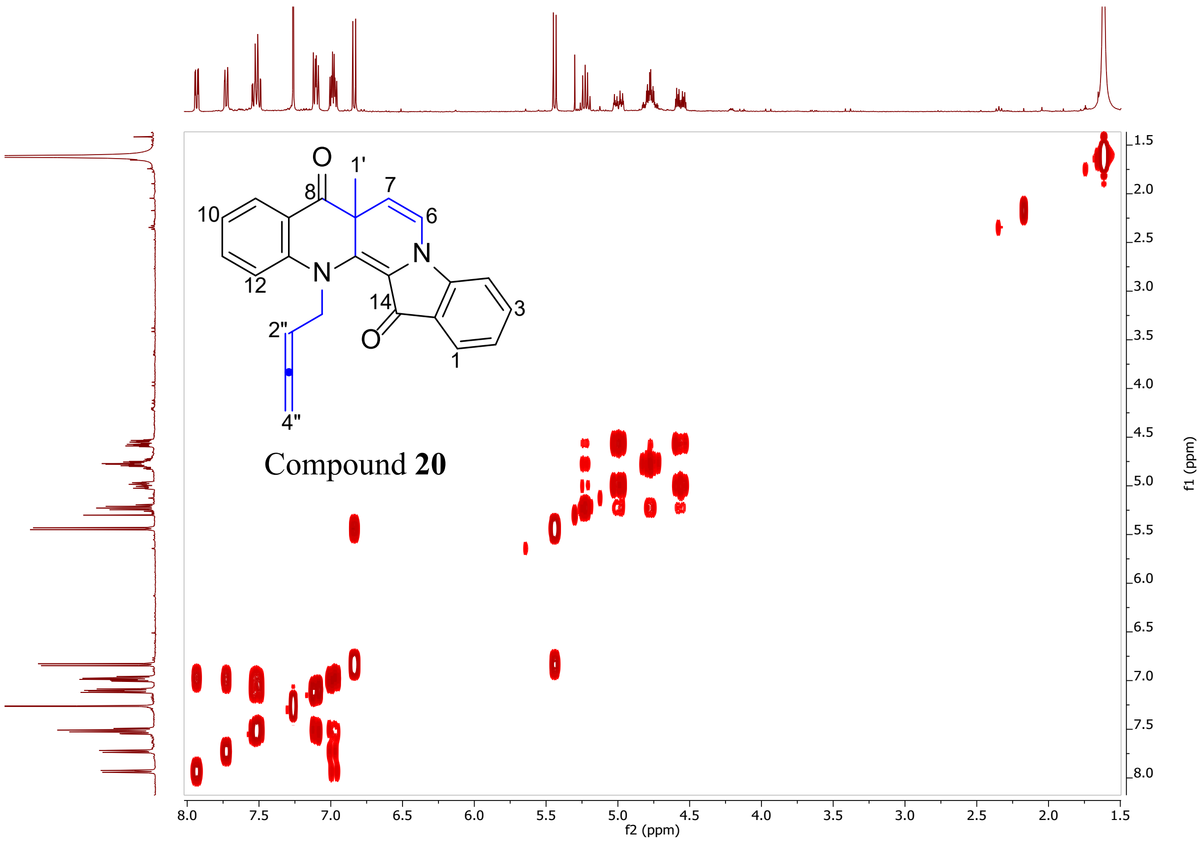

Figure S21. COSY spectrum of **20** ($CDCl_3$)

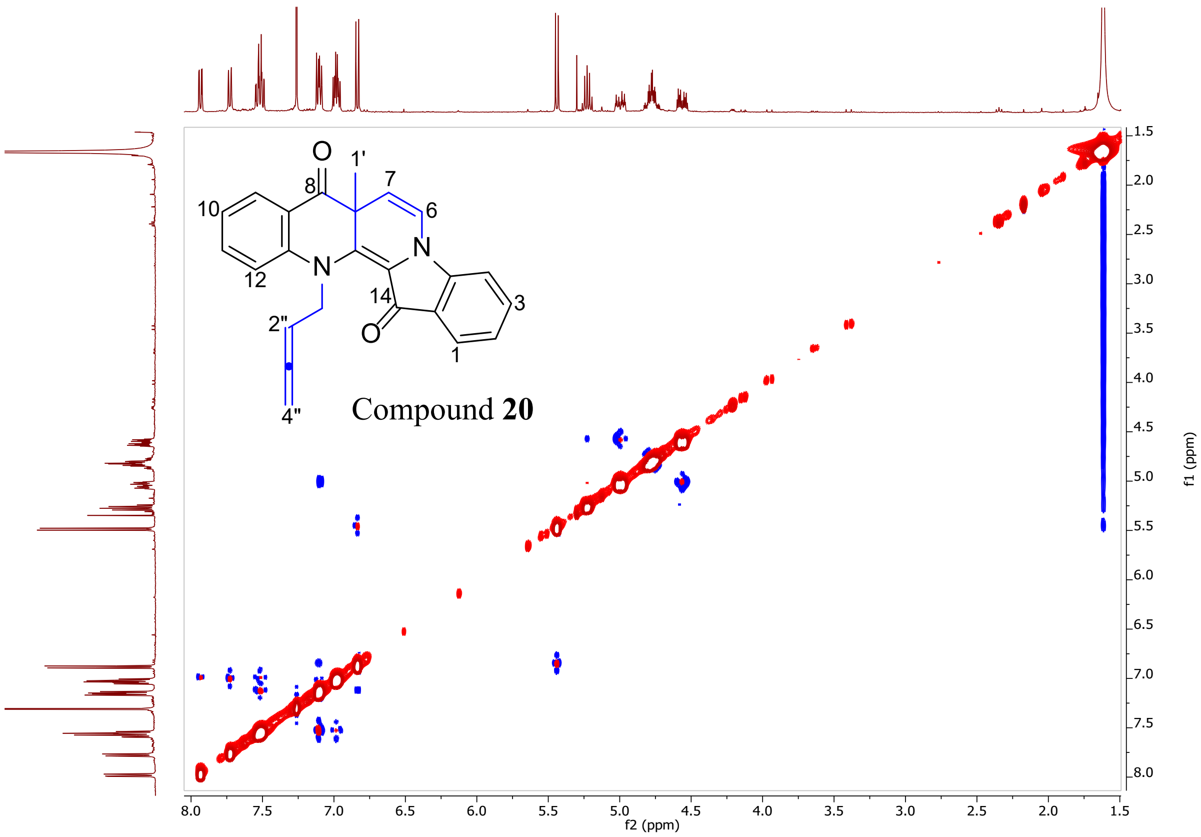

Figure S22. NOESY spectrum of **20** ($CDCl_3$)

NMR Spectra for 9*H*-Benzo[6',7']azepino[2',3':3,4]pyrido[1,2-*a*]indole-9,15(14*H*)-dione (21)

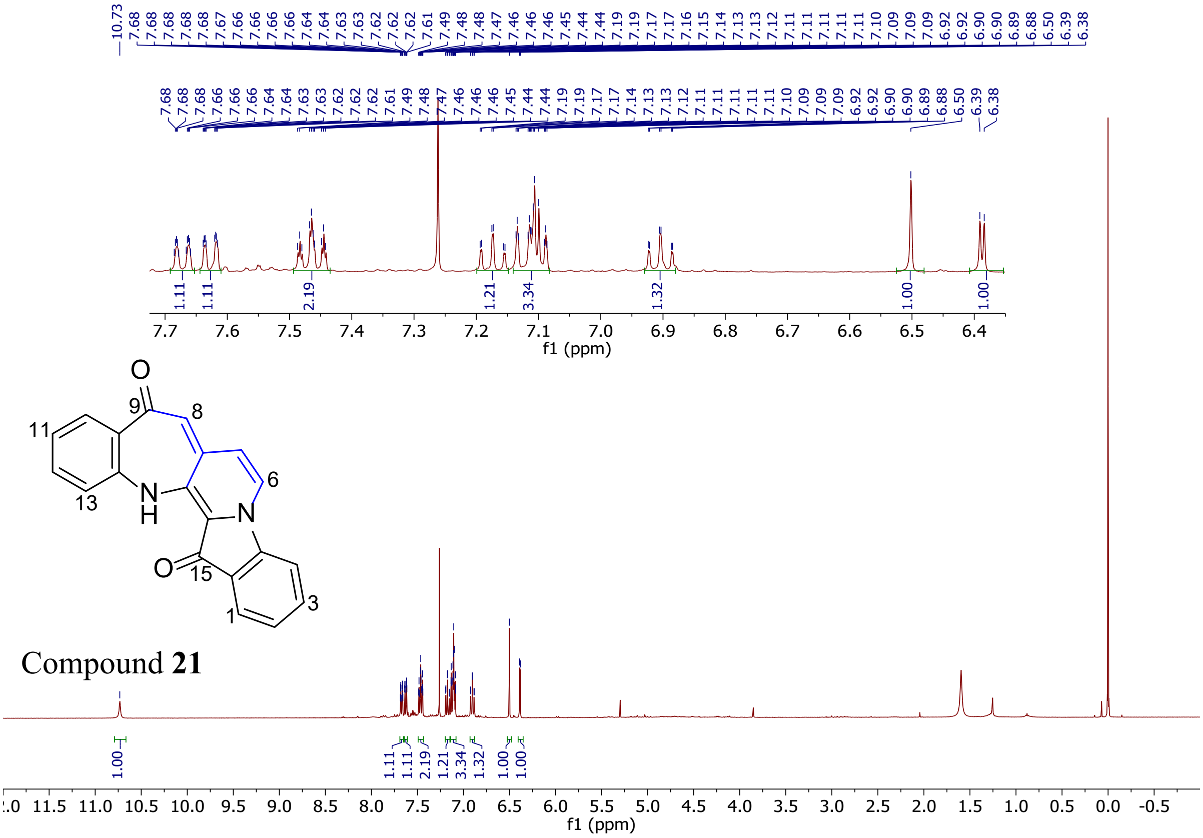

Figure S23. ^{1}H NMR spectrum of **21** (400 MHz, $CDCl_3$)

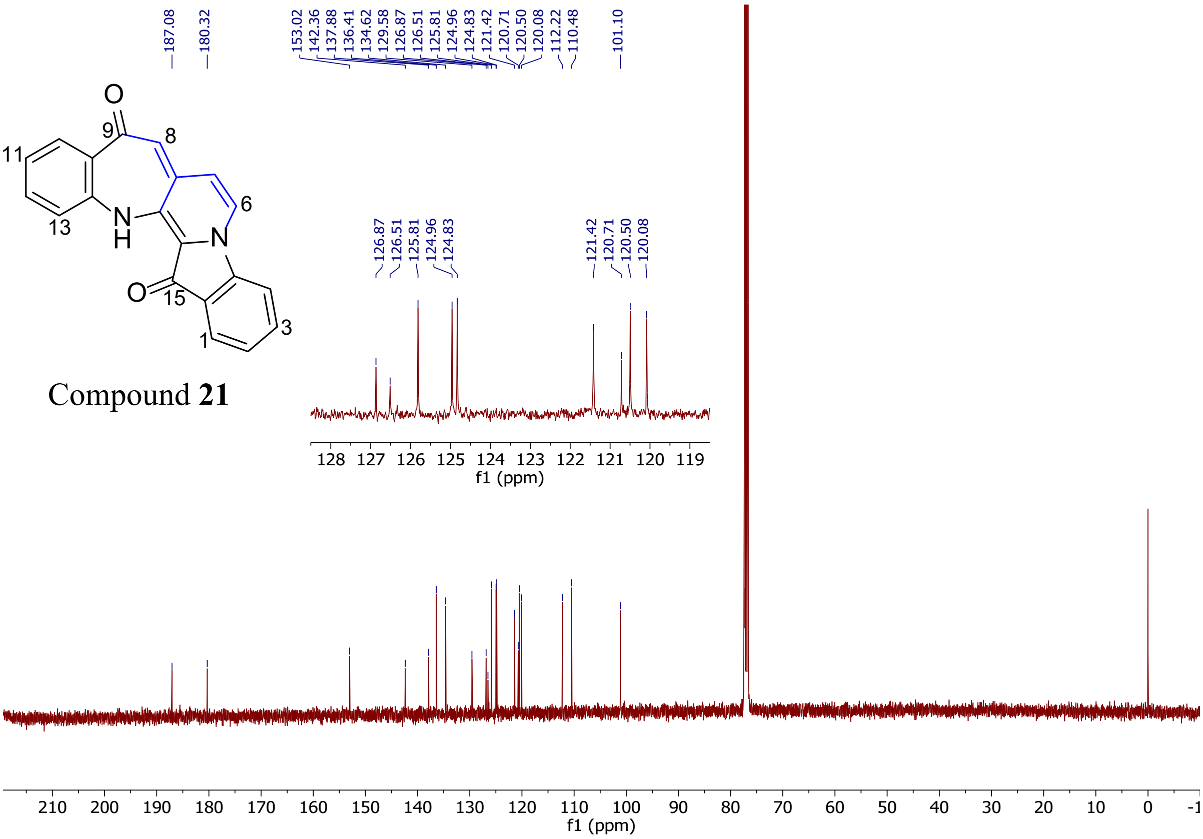

Figure S24. ^{13}C NMR spectrum of **21** (100 MHz, $CDCl_3$)

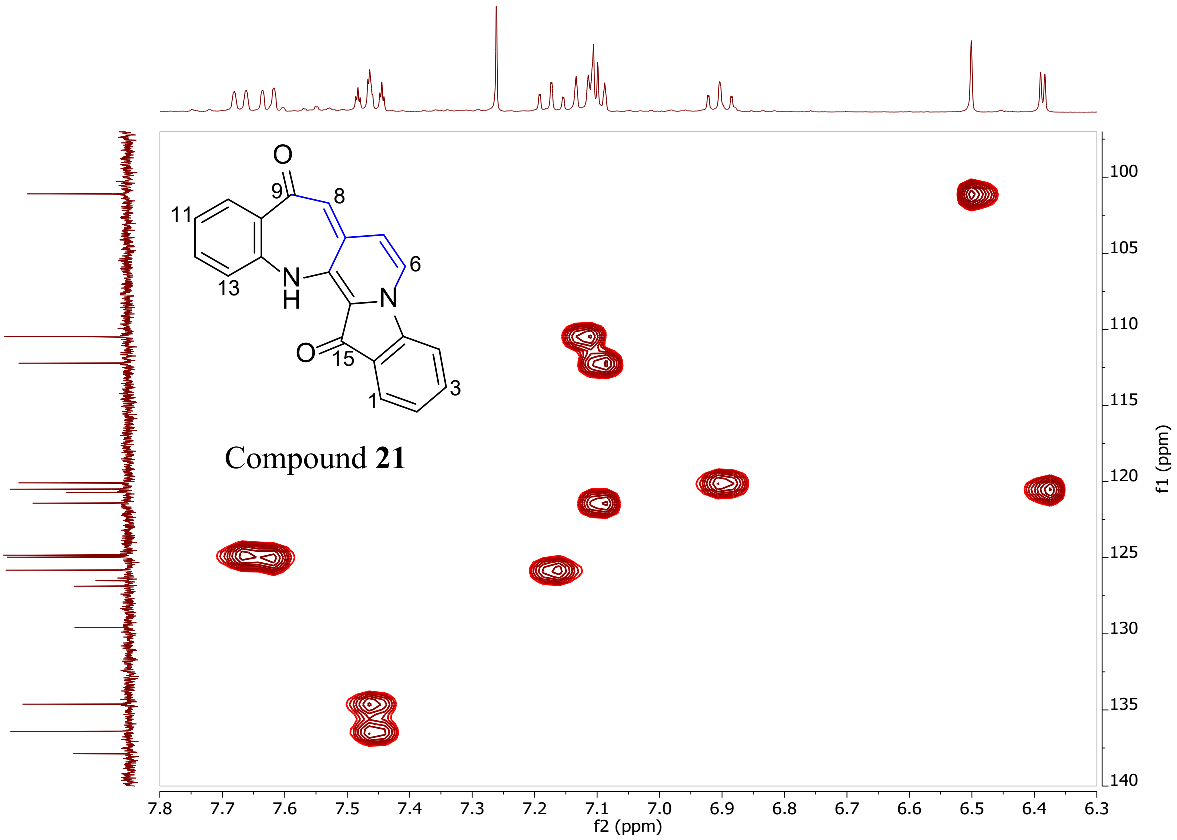

Figure S25. HSQC spectrum of **21** ($CDCl_3$)

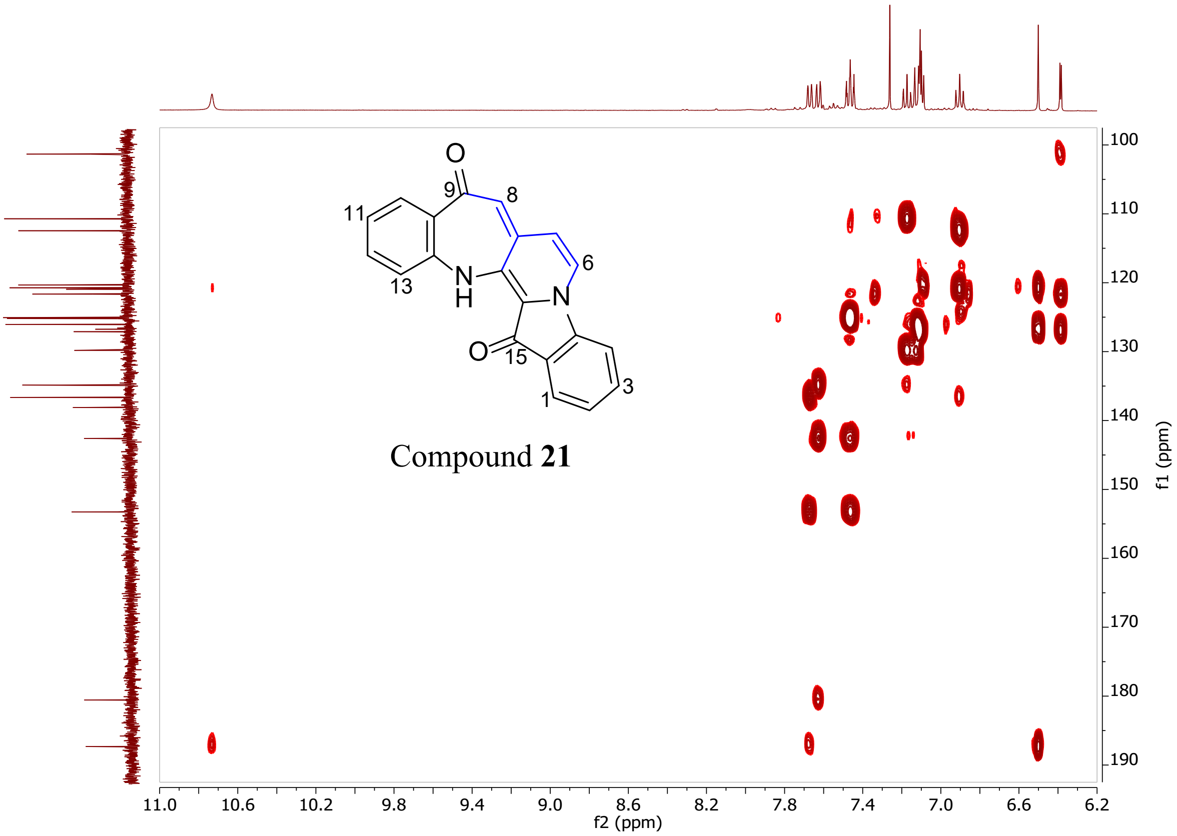

Figure S26. HMBC spectrum of **21** ($CDCl_3$)

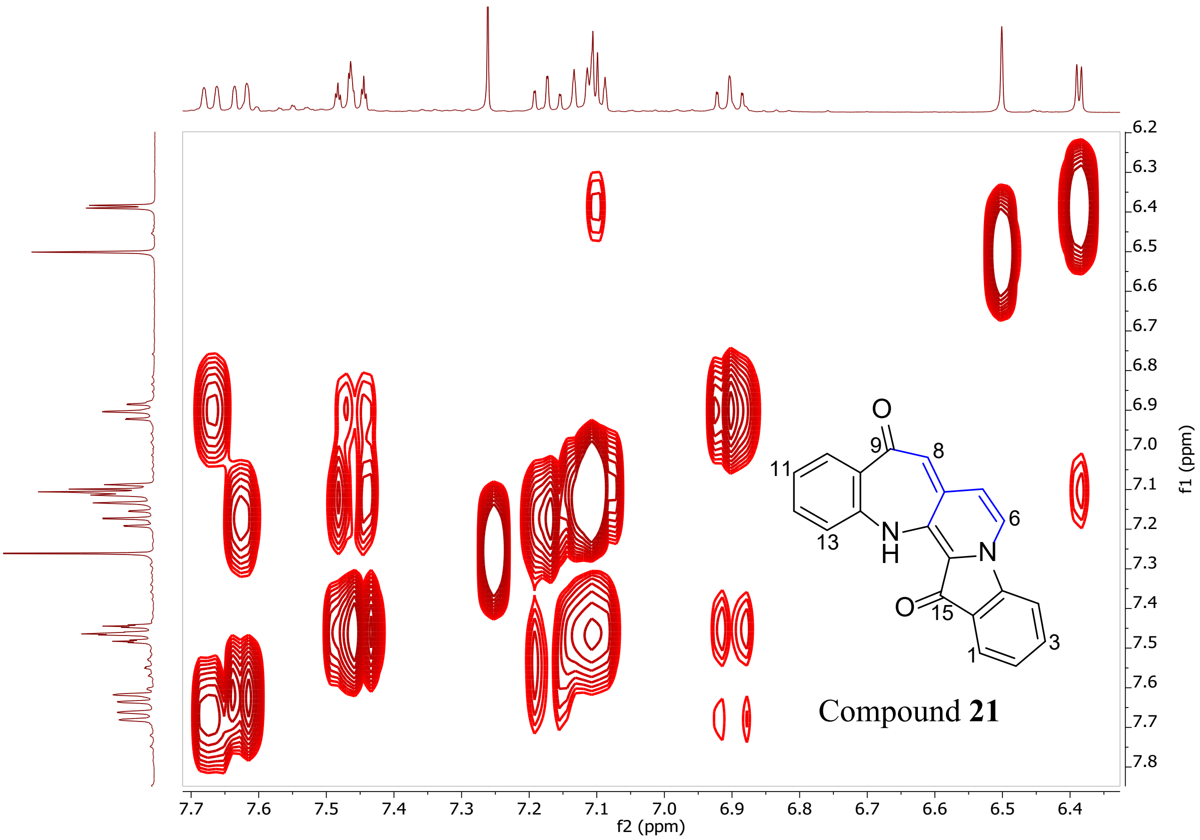

Figure S27. COSY spectrum of **21** ($CDCl_3$)

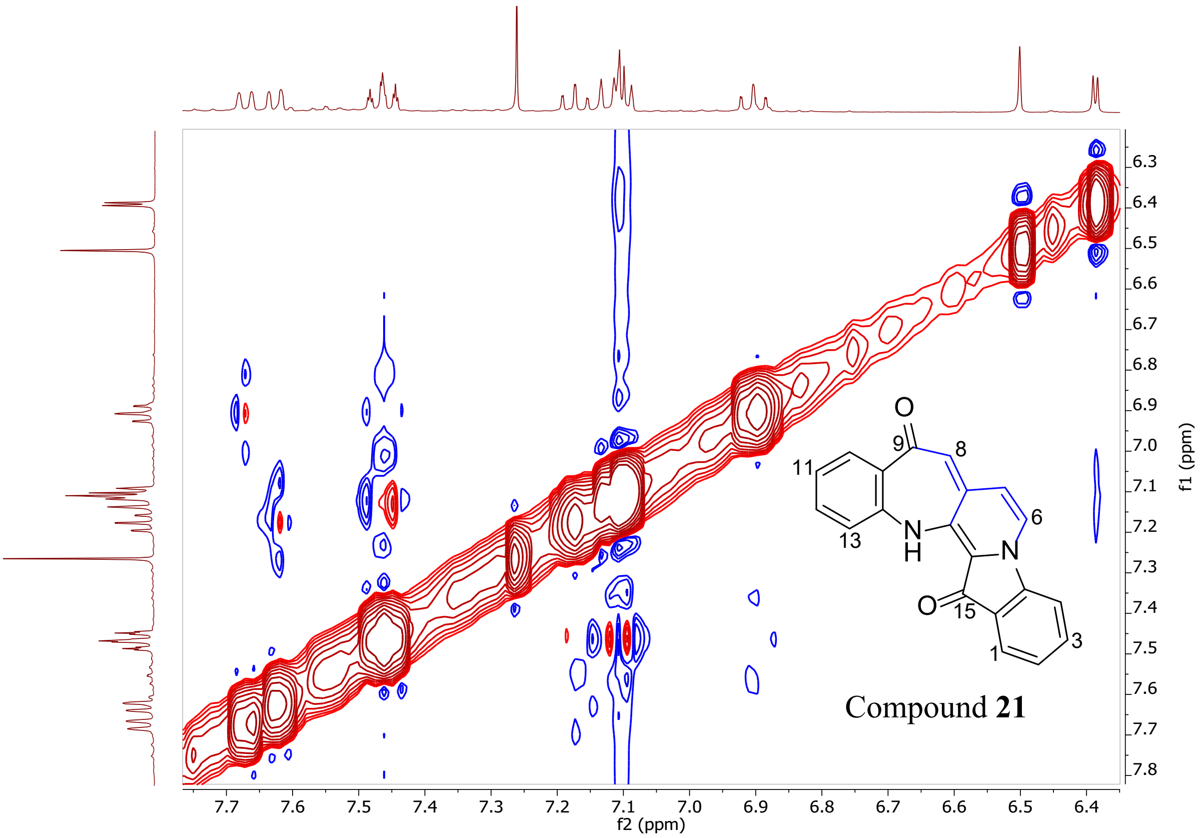

Figure S28. NOESY spectrum of **21** ($CDCl_3$)

NMR Spectra for 5-(Buta-2,3-dien-1-yl)-5*H*-benzo[6',7']azepino[4',3':3,4]pyrido[1,2-*a*]indole-14,15-dione (22)

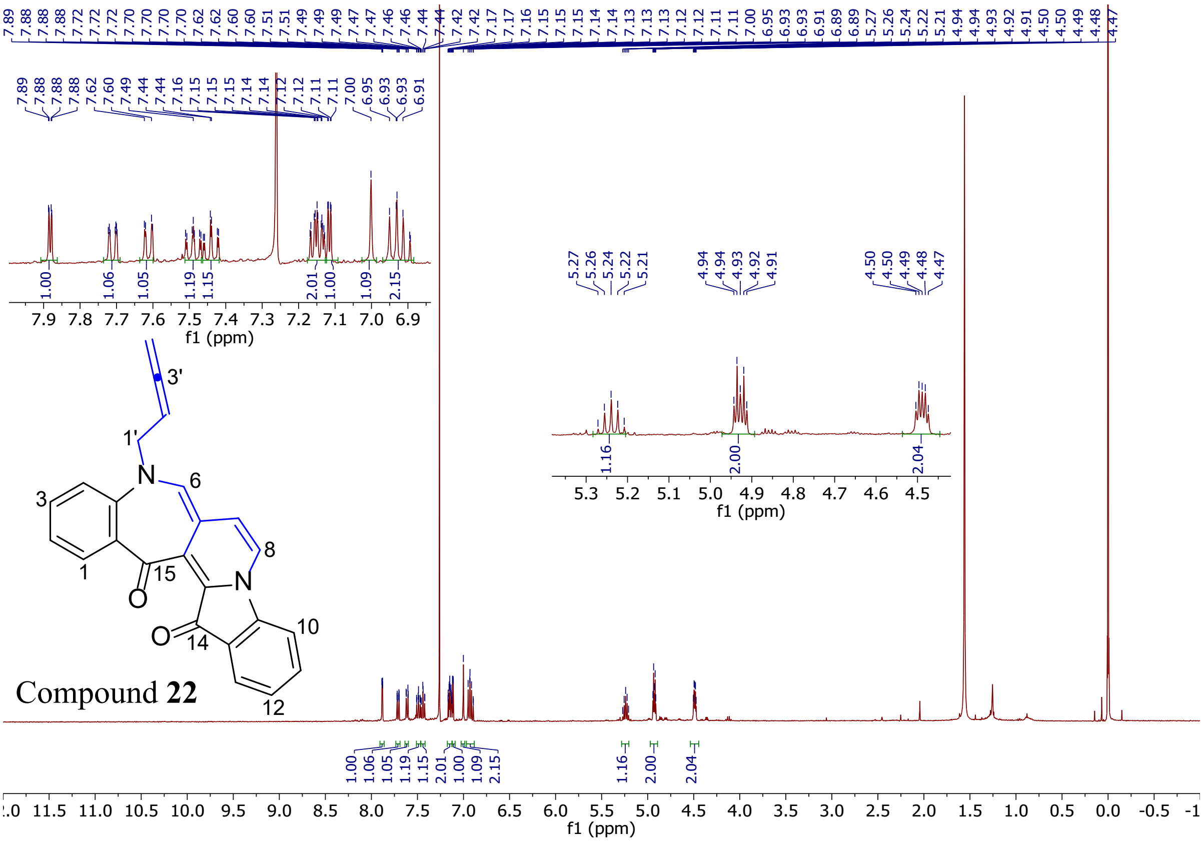

Figure S29. ^{1}H NMR spectrum of **22** (400 MHz, $CDCl_3$)

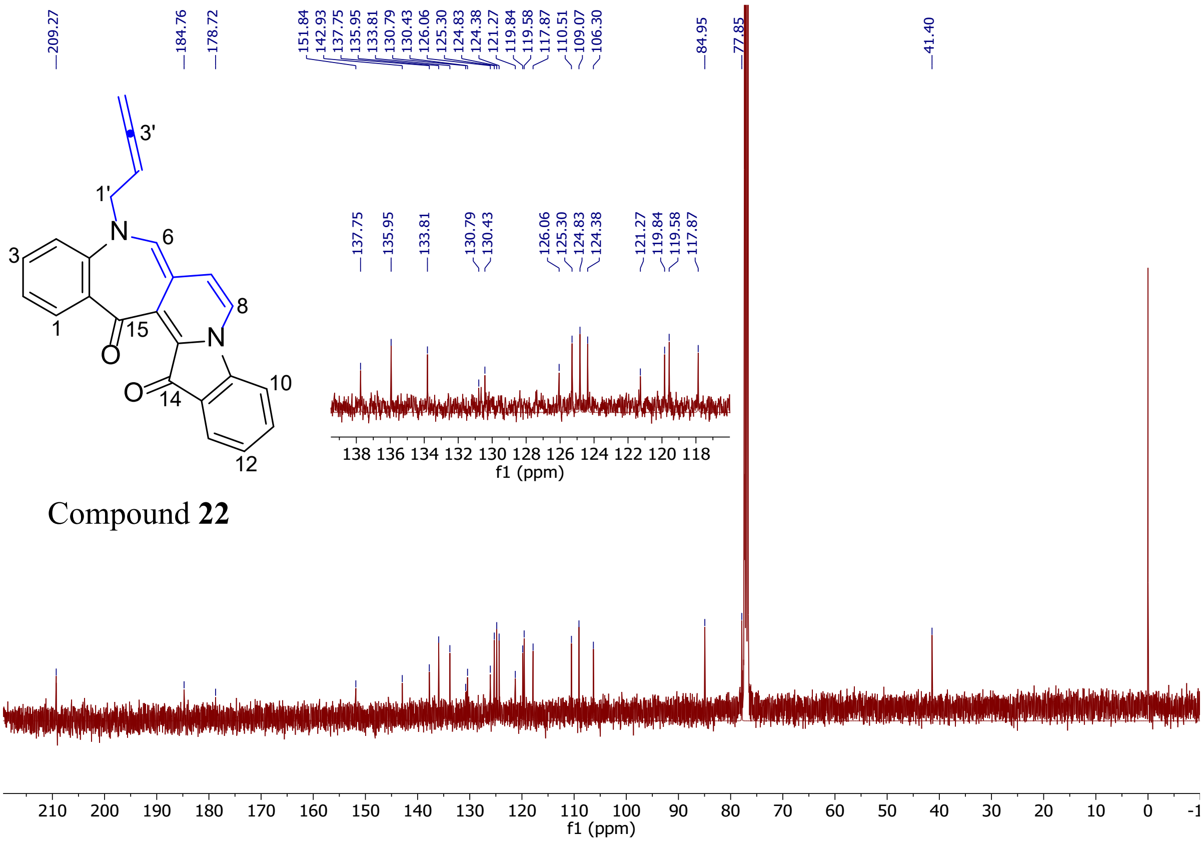

Figure S30. ^{13}C NMR spectrum of **22** (100 MHz, $CDCl_3$)

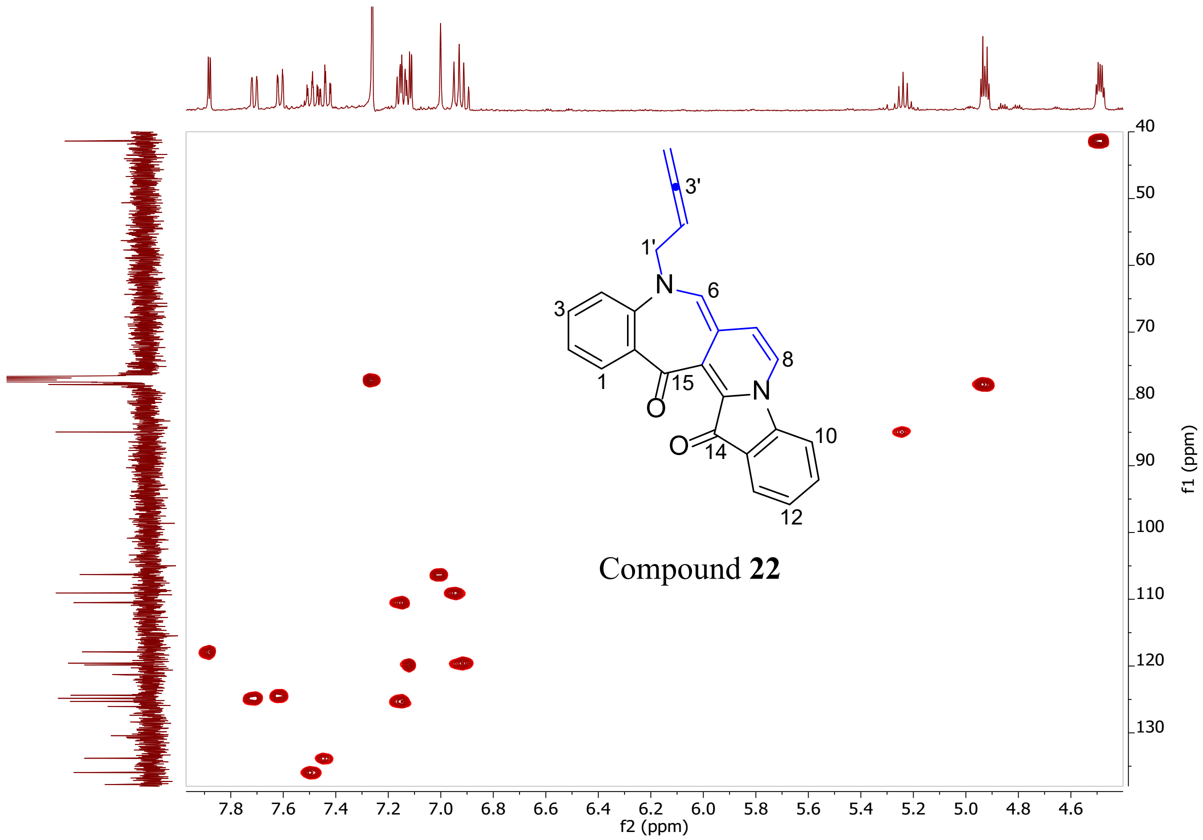

Figure S31. HSQC spectrum of **22** ($CDCl_3$)

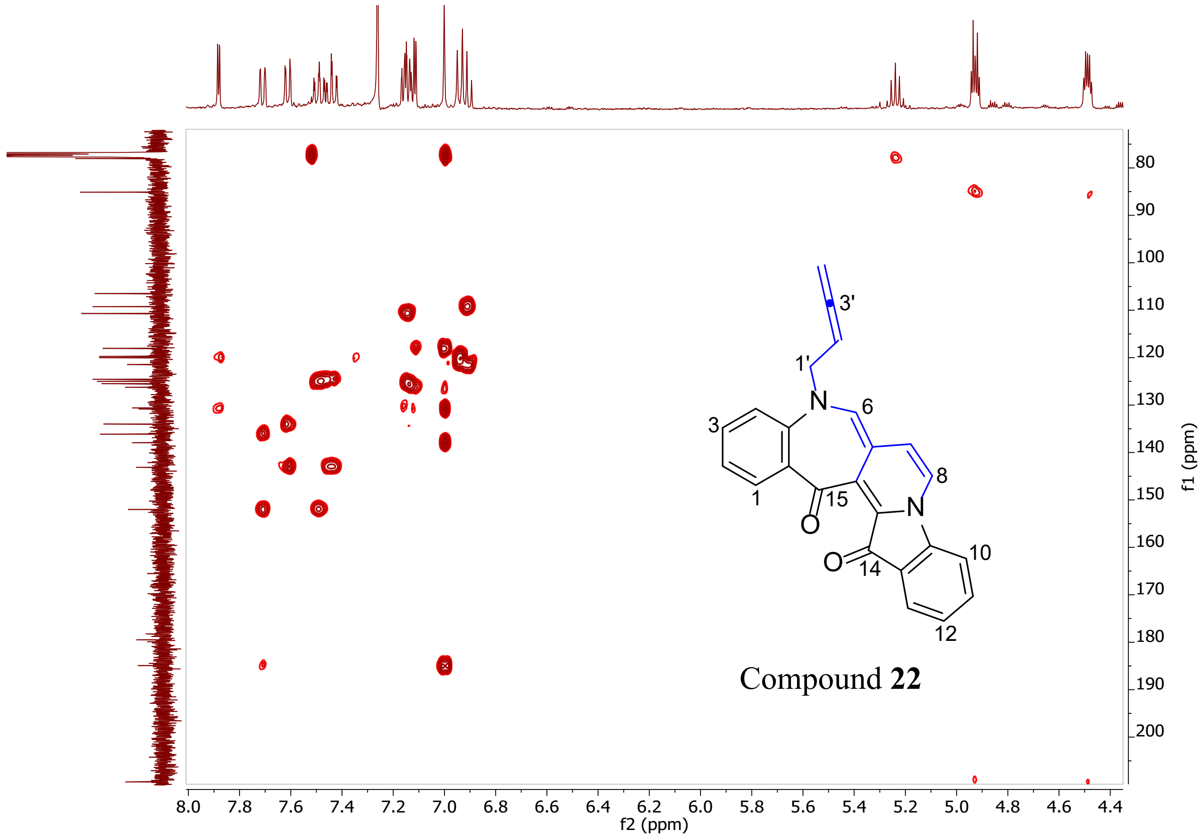

Figure S32. HMBC spectrum of **22** ($CDCl_3$)

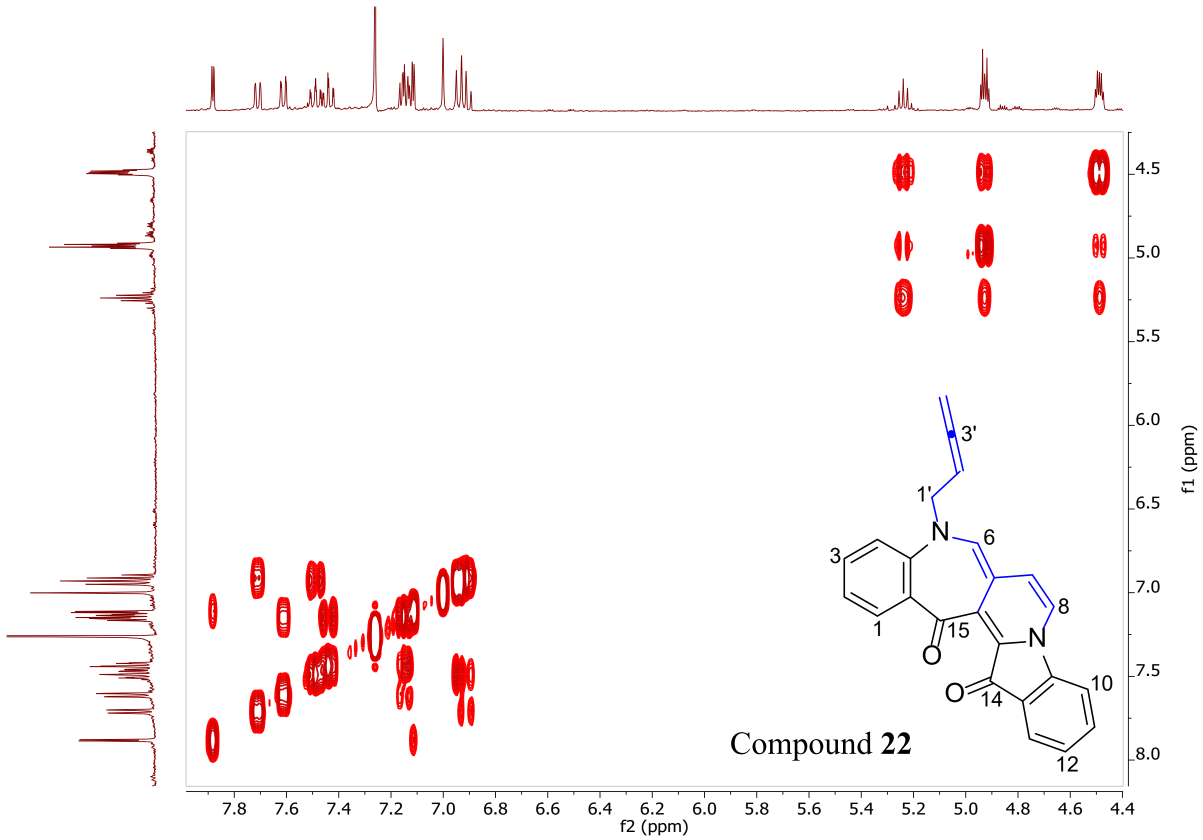

Figure S33. COSY spectrum of **22** ($CDCl_3$)

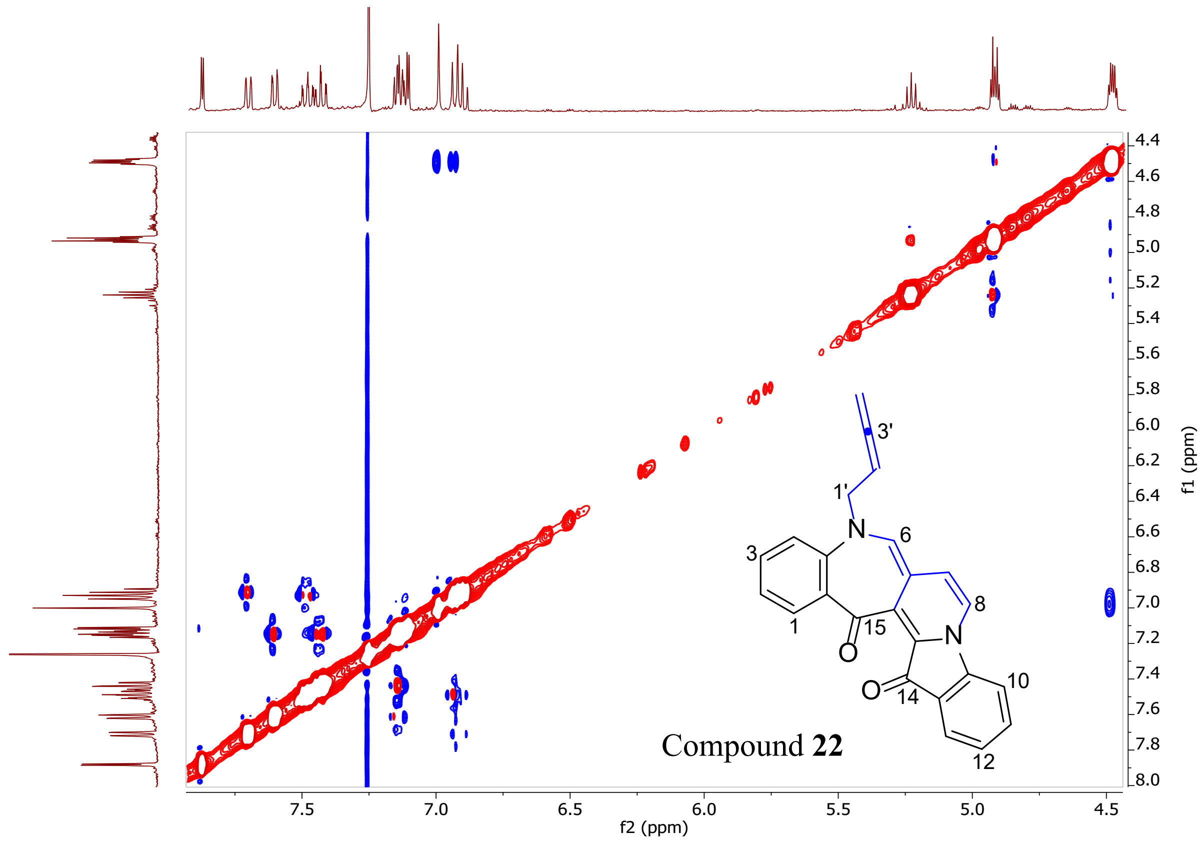

Figure S34. NOESY spectrum of **22** ($CDCl_3$)

NMR Spectra for 1-(Buta-2,3-dien-1-yl)indoline-2,3-dione (23)

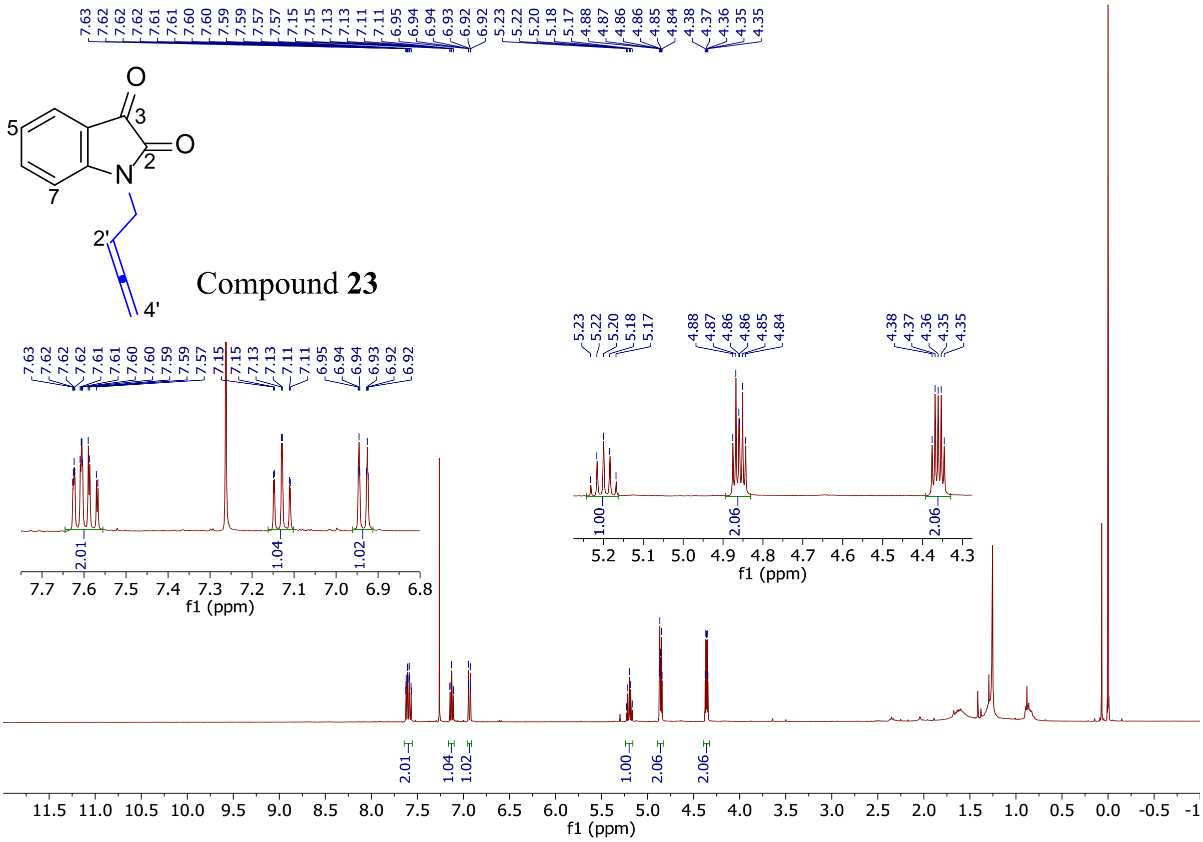

Figure S35. ^{1}H NMR spectrum of **23** (400 MHz, $CDCl_3$)

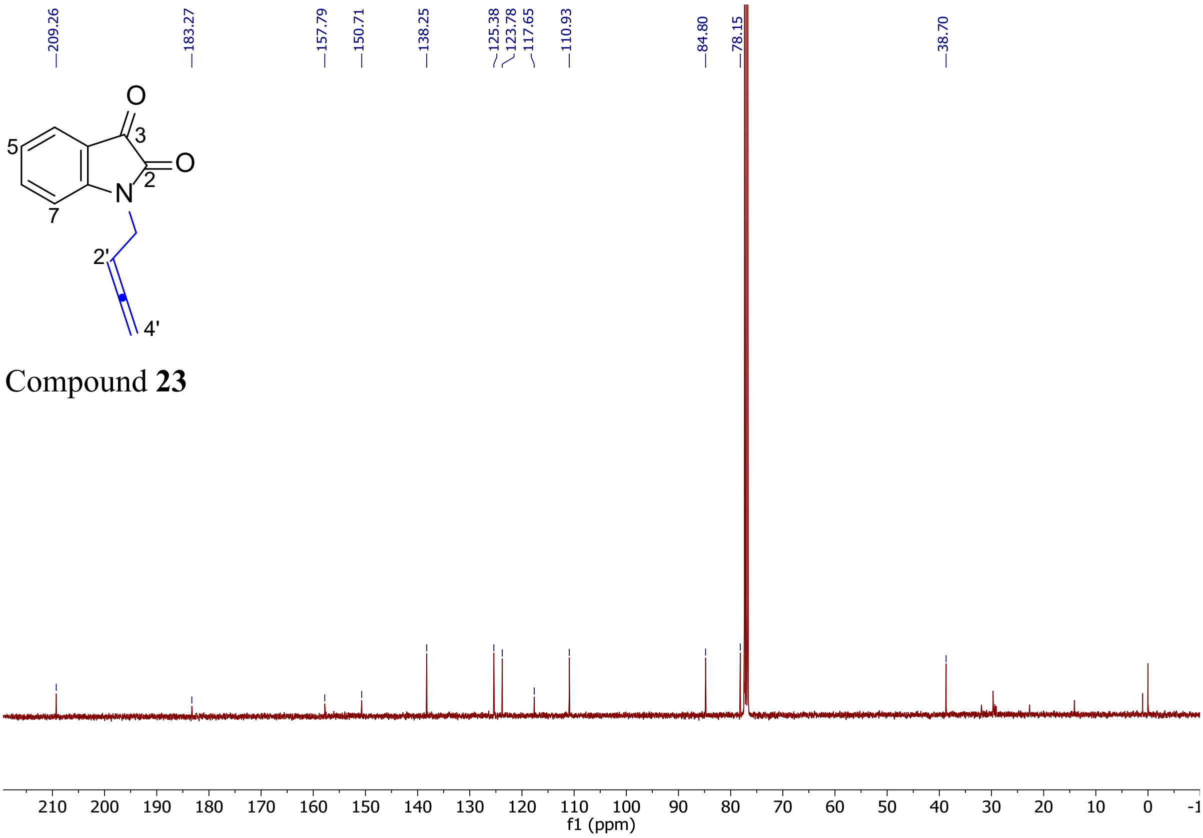

Figure S36. ^{13}C NMR spectrum of **23** (100 MHz, $CDCl_3$)

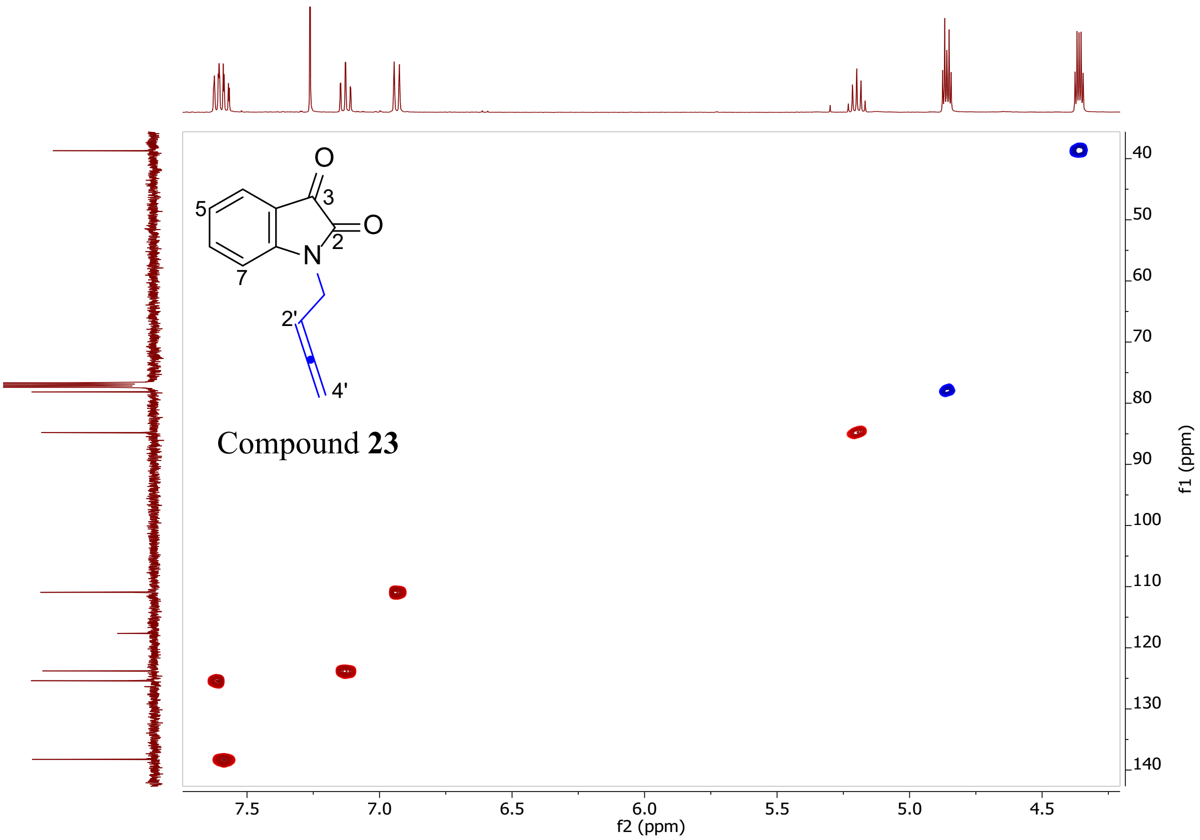

Figure S37. HSQC spectrum of **23** ($CDCl_3$)

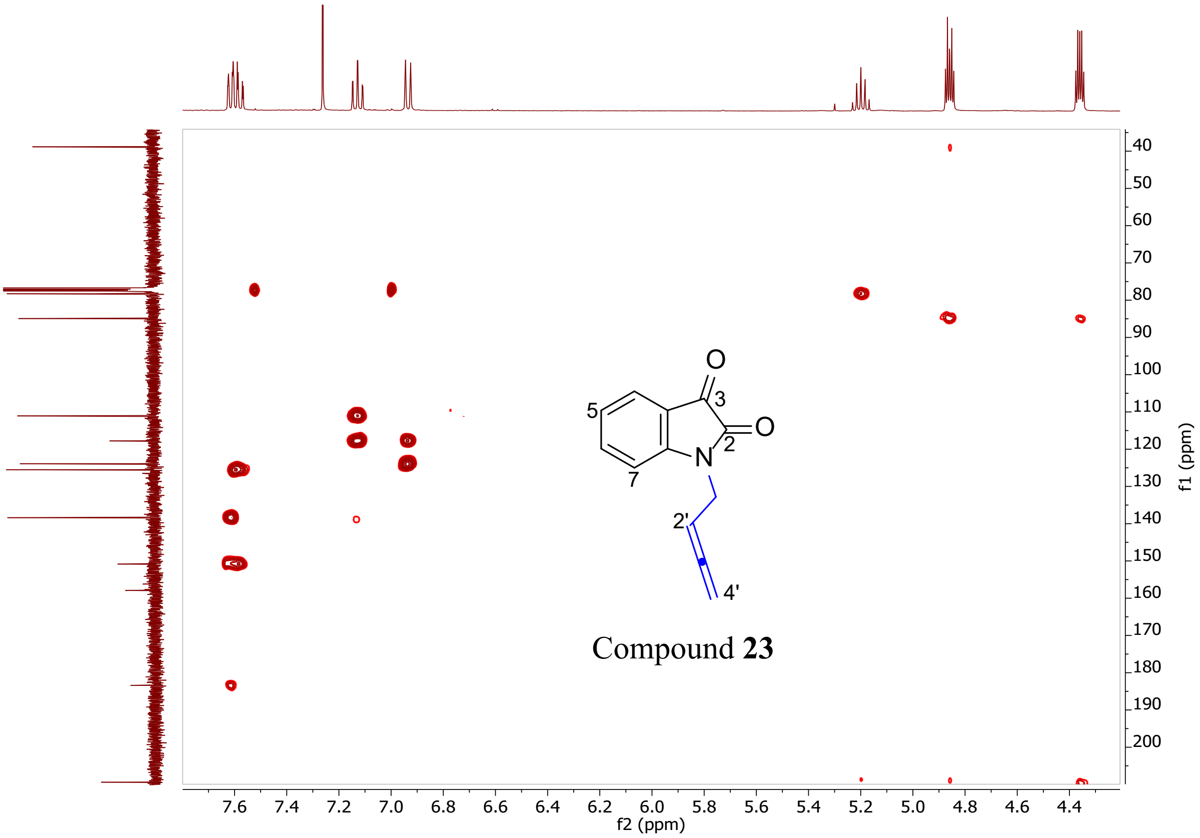

Figure S38. HMBC spectrum of **23** ($CDCl_3$)

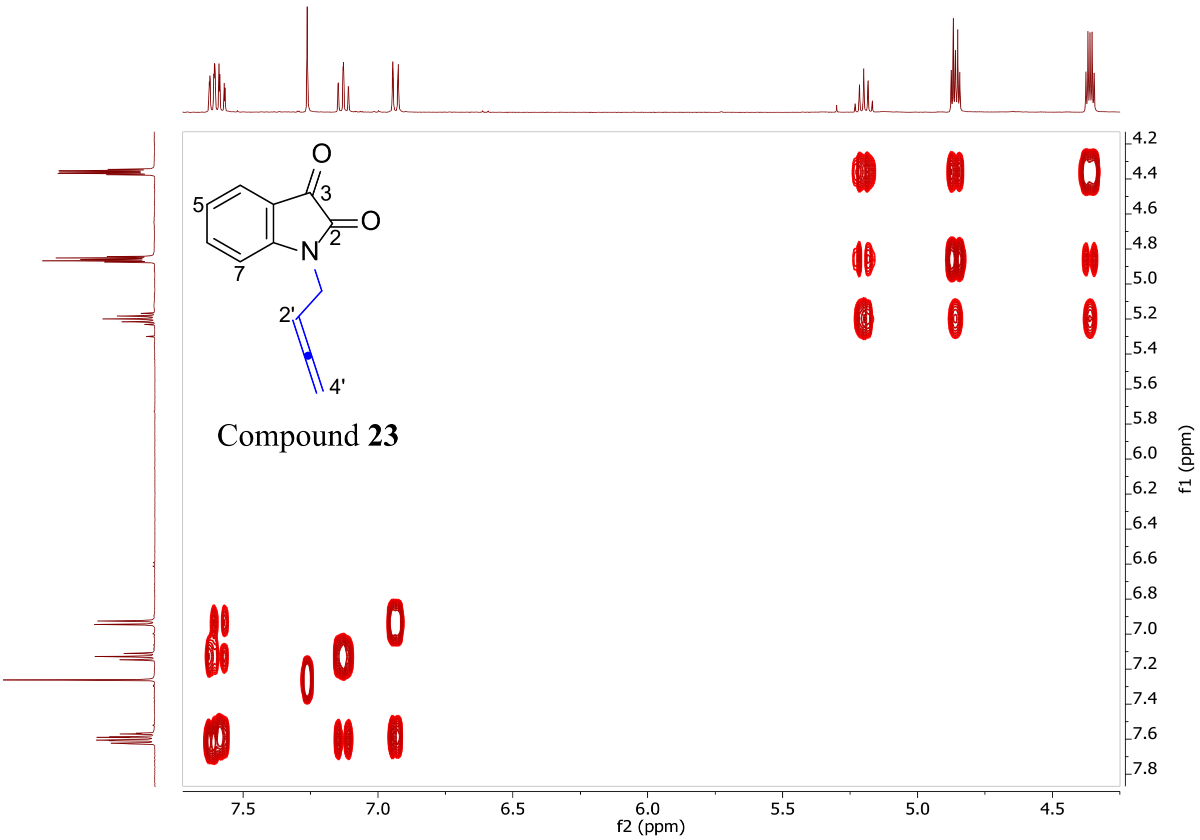

Figure S39. COSY spectrum of **23** ($CDCl_3$)

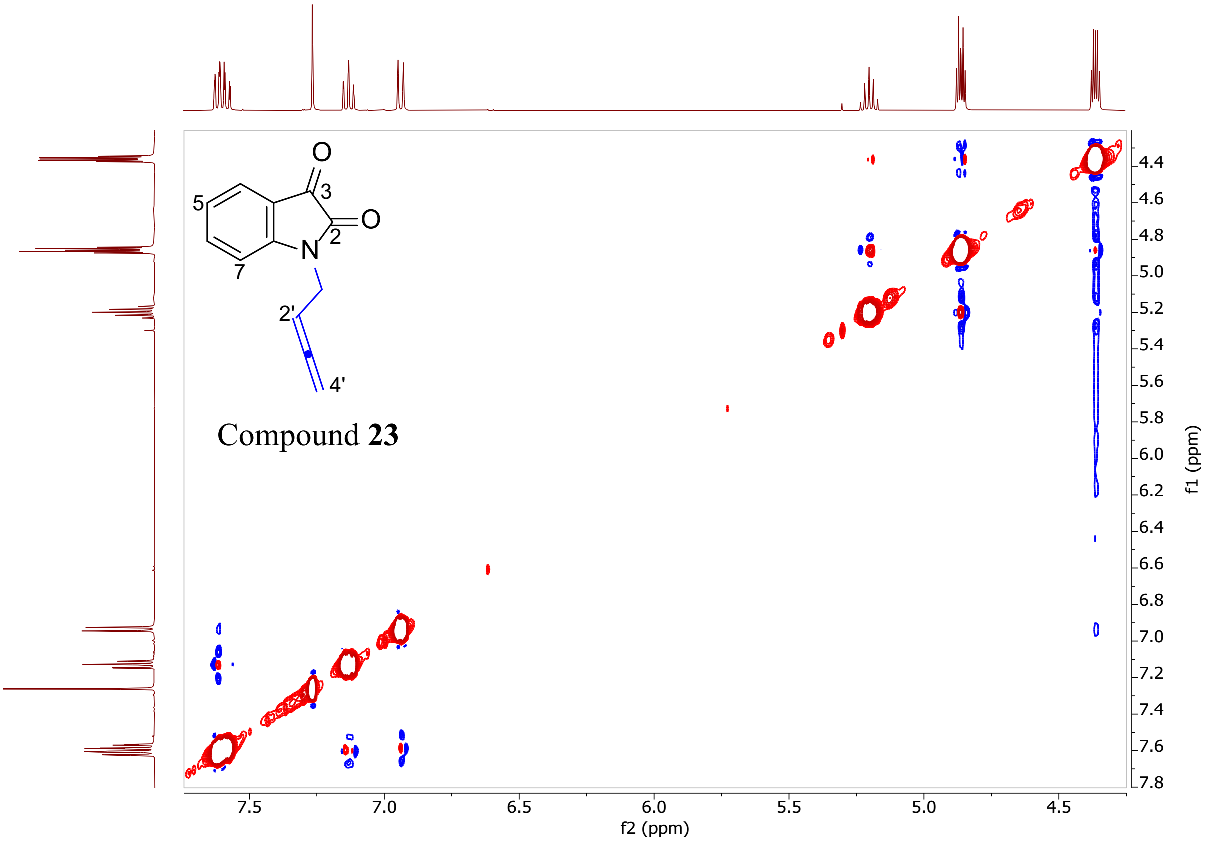

Figure S40. NOESY spectrum of **23** ($CDCl_3$)

NMR Spectra for Indolo[2,1-*b*]quinazoline-6,12-dione (24)

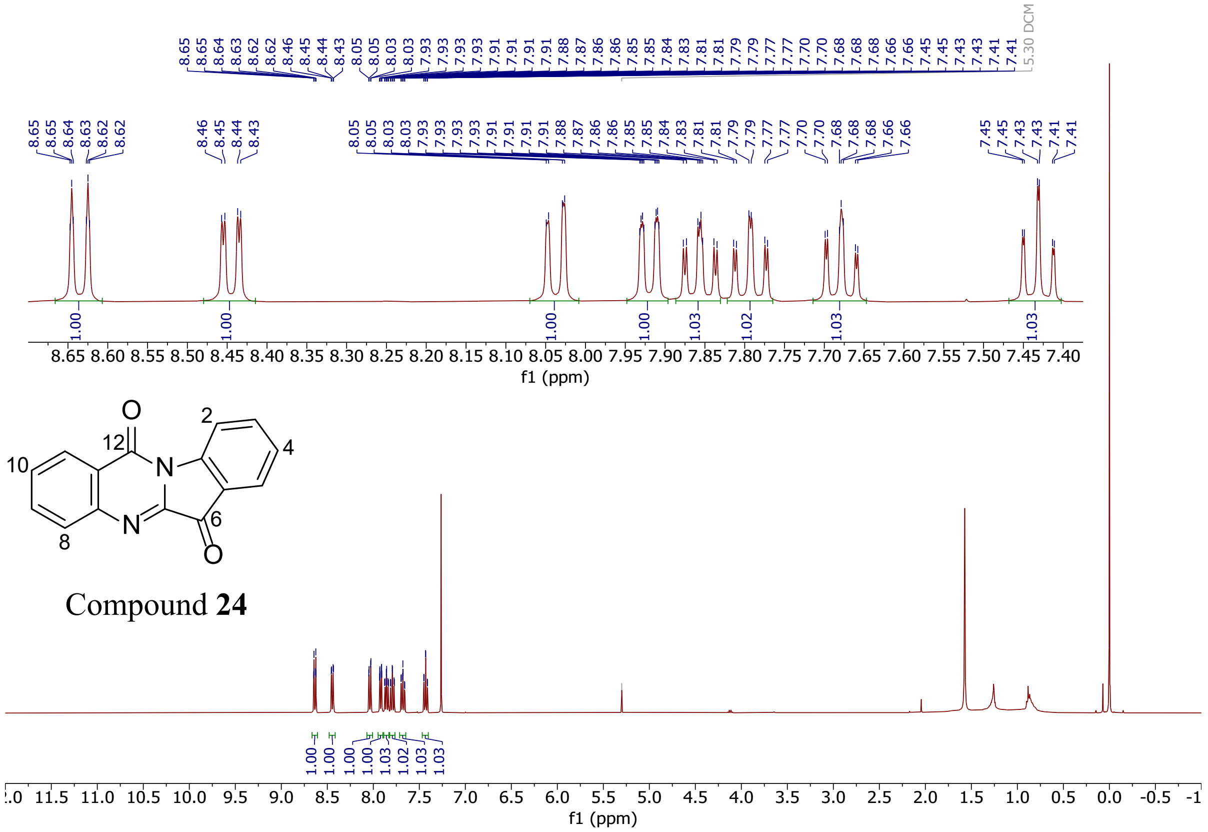

Figure S41. ^{1}H NMR spectrum of **24** (400 MHz, $CDCl_3$)

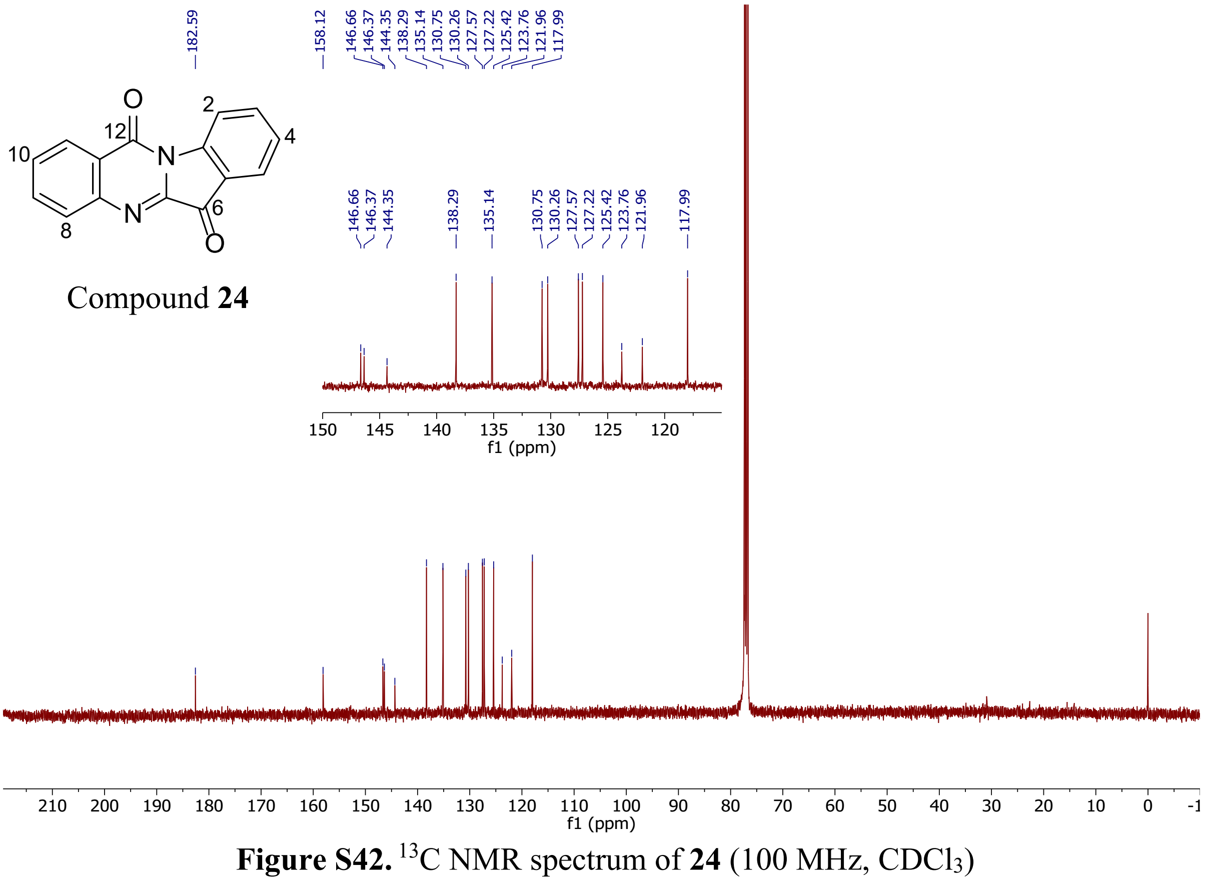

Figure S42. ^{13}C NMR spectrum of **24** (100 MHz, $CDCl_3$)

NMR Spectra for (*E*)-1-Methyl-[2,2'-biindolinylidene]-3,3'-dione (25)

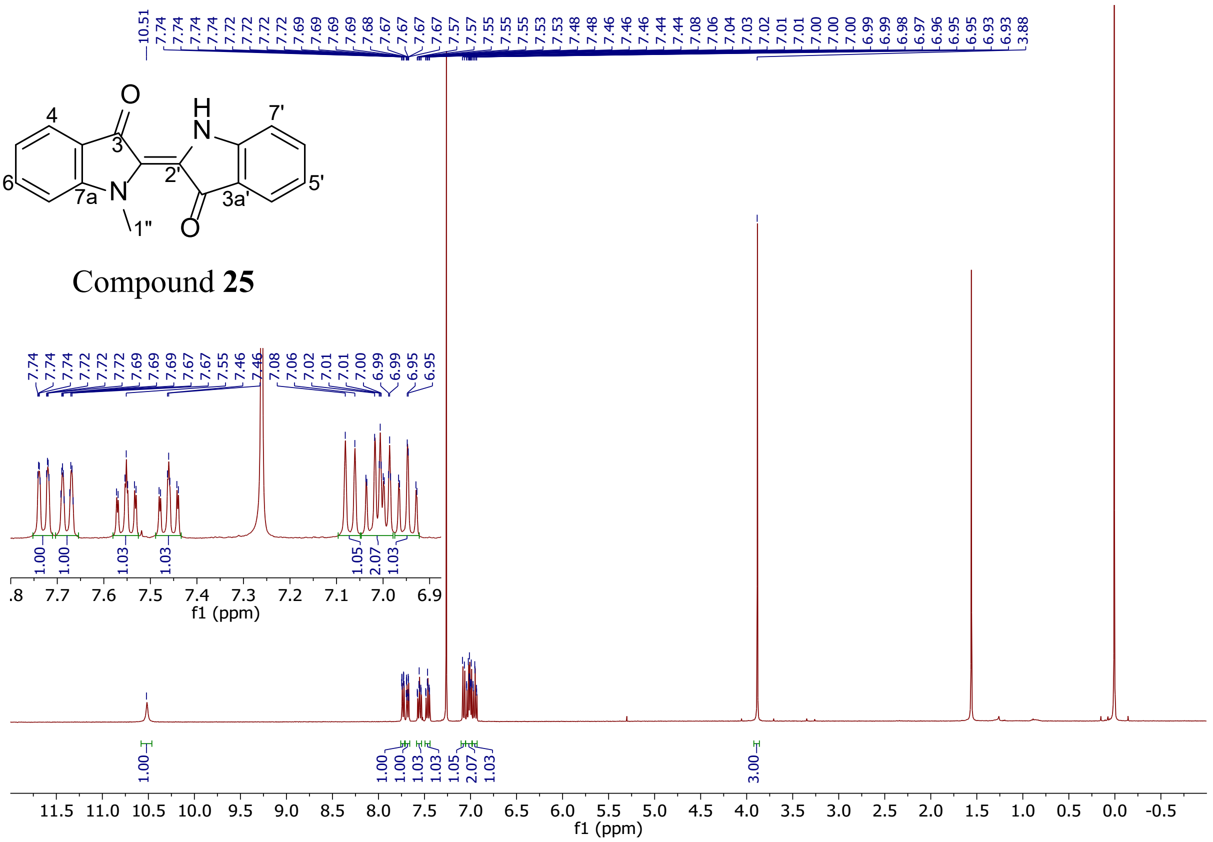

Figure S43. ^{1}H NMR spectrum of **25** (400 MHz, $CDCl_3$)

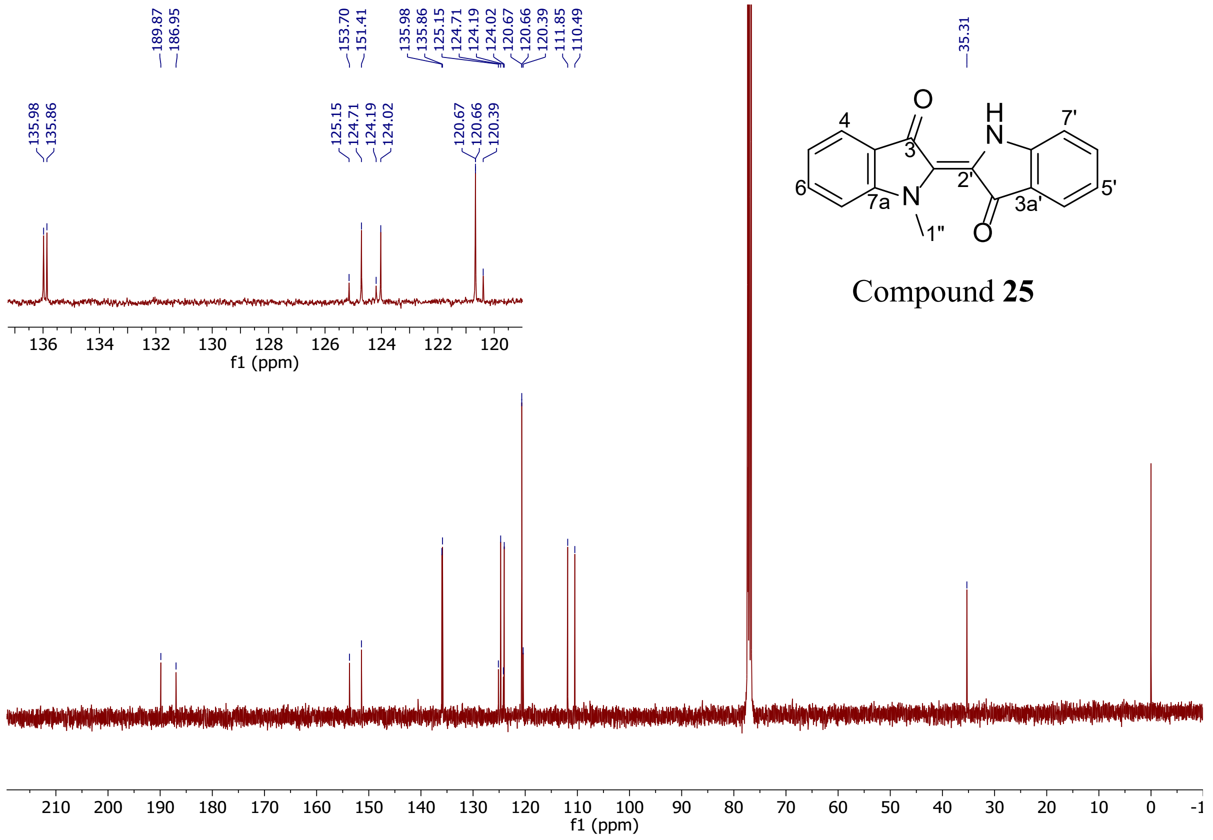

Figure S44. ^{13}C NMR spectrum of **25** (100 MHz, $CDCl_3$)

NMR Spectra for 7a,13-Dimethylbenzo[*b*]indolo[1,2-*h*][1,7]naphthyridine-8,14(7a*H*,13*H*)-dione (26)

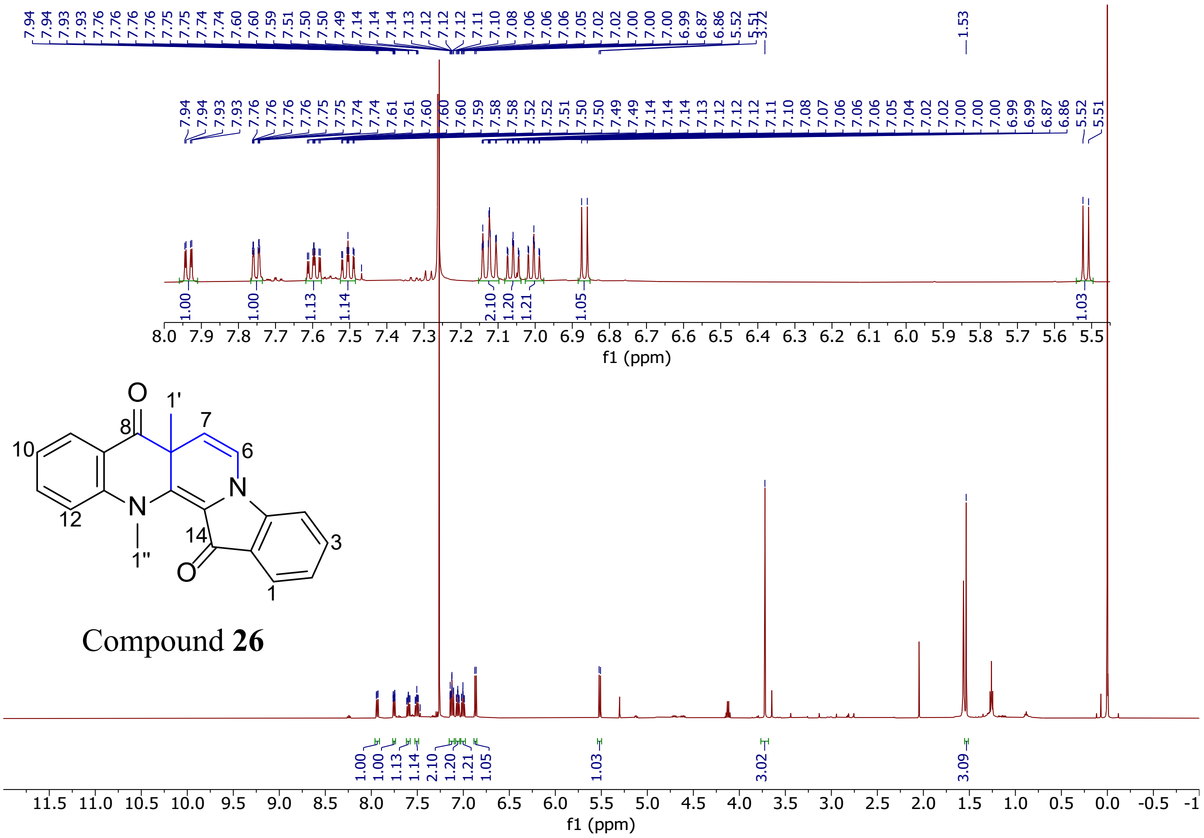

Figure S45. ^{1}H NMR spectrum of **26** (500 MHz, $CDCl_3$) with EtOAc residue/impurities

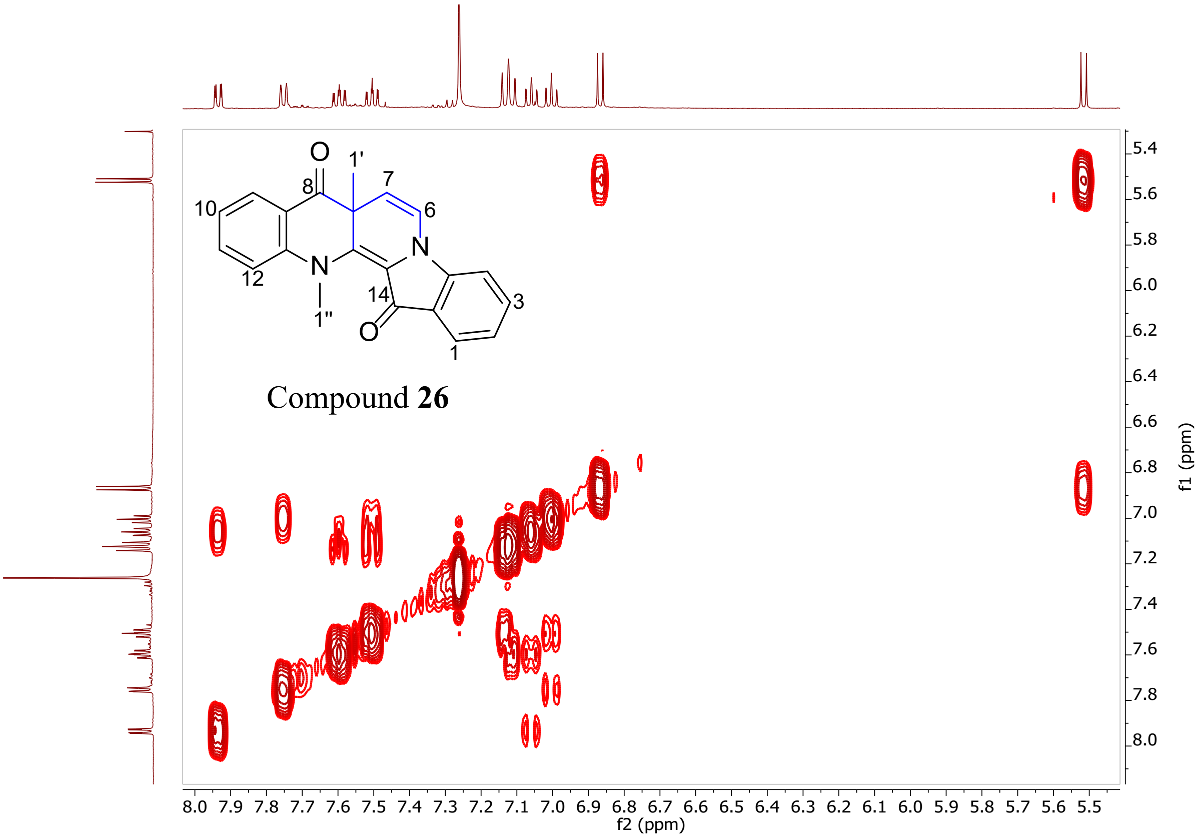

Figure S46. COSY spectrum of **26** ($CDCl_3$)

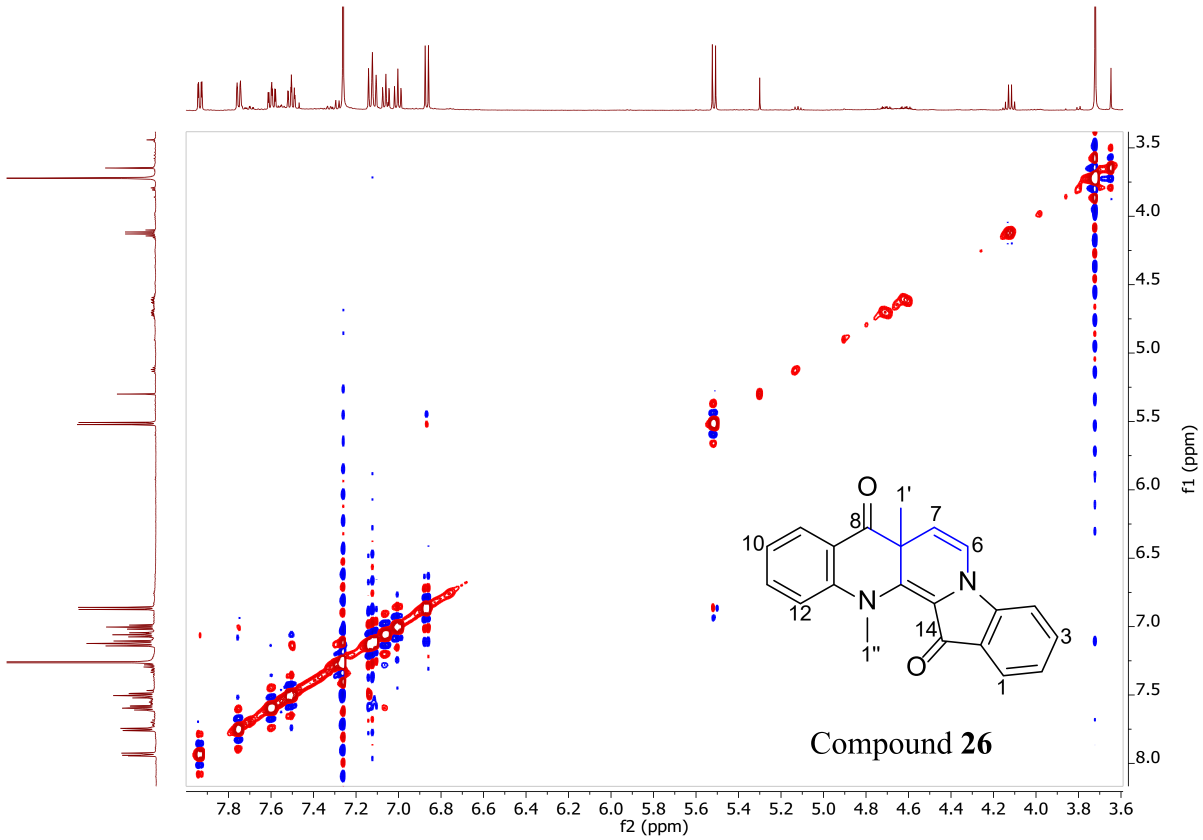

Figure S47. NOESY spectrum of **26** ($CDCl_3$)

X-Ray Crystallography Data for 1-(Buta-2,3-dien-1-yl)indoline-2,3-dione (23)

Summary of Data - Deposition Number 2453855

Compound Name:
Data Block Name: data_exp_400_du
Unit Cell Parameters: a 6.7094(2) b 5.2453(2) c 14.6744(6) P21

Table S1. Crystal data and structure refinement for compound 23

Identification code	exp_400_DU
Empirical formula	$C_{12}H_9NO_2$
Formula weight	199.20
Temperature/K	295(1)
Crystal system	monoclinic
Space group	$P2_1$
a/Å	6.7094(2)
b/Å	5.2453(2)
c/Å	14.6744(6)
α/°	90
β/°	92.715(4)
γ/°	90
Volume/Å^3	515.85(3)
Z	2
ρ_{calc}g/cm^3	1.282
μ/mm^{-1}	0.088
F(000)	208.0
Crystal size/mm^3	0.51 × 0.26 × 0.07
Radiation	Mo Kα (λ = 0.71073)
2Θ range for data collection/°	6.08 to 57.4
Index ranges	-8 ≤ h ≤ 8, -7 ≤ k ≤ 7, -19 ≤ l ≤ 19
Reflections collected	10257
Independent reflections	2646 [R_{int} = 0.0254, R_{sigma} = 0.0296]
Data/restraints/parameters	2646/3/144
Goodness-of-fit on F^2	1.006
Final R indexes [I>=2σ (I)]	R_1 = 0.0396, wR_2 = 0.0888
Final R indexes [all data]	R_1 = 0.0795, wR_2 = 0.1029
Largest diff. peak/hole / e Å^{-3}	0.08/-0.11
Flack parameter	-0.5(7)

Table S2. Fractional Atomic Coordinates (×10^4) and Equivalent Isotropic Displacement Parameters (Å^2×10^3) for compound 23. U_{eq} is defined as 1/3 of the trace of the orthogonalised U_{IJ} tensor.

Atom	*x*	*y*	*z*	U(eq)
C2	1553(4)	2511(5)	2406.2(17)	81.4(8)
C3	542(4)	3846(5)	3205.3(17)	75.6(7)
C3A	1921(3)	5813(4)	3526.5(14)	64.3(6)
C4	1837(4)	7572(5)	4221.2(16)	75.5(6)
C5	3408(4)	9237(5)	4368.7(18)	83.7(7)
C6	5023(4)	9124(5)	3817.1(17)	74.9(7)
C7	5149(3)	7345(4)	3118.6(16)	66.7(6)
C7A	3573(3)	5688(4)	2980.5(13)	59.5(5)
C8	4826(4)	3002(6)	1674.4(17)	86.9(8)
C9	5178(5)	5004(6)	984.0(18)	95.9(9)
C10	6935(5)	5826(6)	816.2(17)	92.0(8)
C11	8695(7)	6609(9)	674(3)	124.1(13)
N1	3329(3)	3742(4)	2322.0(13)	70.5(5)
O2	923(3)	727(5)	1956.7(14)	115.9(7)
O3	-1082(2)	3223(4)	3471.4(14)	105.7(7)

Table S3. Anisotropic Displacement Parameters (Å^2×10^3) for compound 23. The Anisotropic displacement factor exponent takes the form: $-2\pi^2[h^2a^{*2}U_{11}+2hka^*b^*U_{12}+\ldots]$.

Atom	U_{11}	U_{22}	U_{33}	U_{23}	U_{13}	U_{12}
C2	88.8(17)	76.9(18)	76.8(16)	10.1(15)	-14.3(13)	-23.5(14)
C3	67.2(14)	82.2(18)	77.0(15)	20.8(14)	-1.3(11)	-7.8(13)
C3A	63.1(12)	65.8(13)	64.2(13)	13.4(12)	3.6(10)	-3.7(12)
C4	83.8(15)	71.8(16)	72.1(14)	8.4(14)	15.9(11)	6.5(14)
C5	108(2)	71.5(16)	72.3(16)	-3.7(13)	7.0(14)	1.6(15)
C6	82.0(16)	67.1(16)	74.6(15)	4.1(13)	-6.7(12)	-12.9(12)
C7	63.3(12)	70.4(14)	66.5(13)	7.8(12)	2.6(10)	-9.5(12)
C7A	66.3(12)	55.1(11)	56.9(11)	7.4(11)	-0.2(9)	-1.4(11)
C8	110.0(19)	77.1(16)	74.6(15)	-8.7(13)	13.6(14)	-6.9(14)
C9	118(3)	108(2)	62.6(15)	5.2(16)	5.3(15)	-5.9(18)
C10	126(2)	89.2(18)	61.5(15)	-9.6(14)	16.9(15)	-16(2)
C11	139(3)	130(3)	105(3)	-16(2)	28(2)	-42(2)
N1	76.2(12)	71.7(13)	63.9(11)	-1.6(10)	5.6(8)	-10.5(11)
O2	133.1(16)	108.3(15)	104.1(14)	-10.2(14)	-17.6(12)	-46.7(15)
O3	69.5(11)	126.5(18)	121.4(15)	24.3(13)	8.4(10)	-22.3(11)

Table S4. Bond Lengths for compound 23.

Atom	Atom	Length/Å	Atom	Atom	Length/Å
C2	C3	1.549(4)	C5	C6	1.384(3)
C2	N1	1.367(3)	C6	C7	1.392(3)
C2	O2	1.209(3)	C7	C7A	1.377(3)
C3	C3A	1.450(3)	C7A	N1	1.409(3)
C3	O3	1.219(3)	C8	C9	1.486(4)
C3A	C4	1.378(3)	C8	N1	1.467(3)
C3A	C7A	1.399(3)	C9	C10	1.290(4)
C4	C5	1.378(4)	C10	C11	1.276(5)

Table S5. Bond Angles for compound 23.

Atom	Atom	Atom	Angle/°	Atom	Atom	Atom	Angle/°
N1	C2	C3	105.6(2)	C5	C6	C7	122.4(2)
O2	C2	C3	127.5(2)	C7A	C7	C6	117.3(2)
O2	C2	N1	126.9(3)	C3A	C7A	N1	110.94(17)
C3A	C3	C2	105.81(19)	C7	C7A	C3A	120.6(2)
O3	C3	C2	123.4(2)	C7	C7A	N1	128.42(19)
O3	C3	C3A	130.7(3)	N1	C8	C9	113.0(2)
C4	C3A	C3	132.0(2)	C10	C9	C8	122.8(3)
C4	C3A	C7A	121.1(2)	C11	C10	C9	178.2(3)
C7A	C3A	C3	106.9(2)	C2	N1	C7A	110.71(18)
C5	C4	C3A	118.9(2)	C2	N1	C8	124.1(2)
C4	C5	C6	119.7(2)	C7A	N1	C8	125.10(18)

Table S6. Torsion Angles for compound 23.

A	B	C	D	Angle/°	A	B	C	D	Angle/°
C2	C3	C3A	C4	-179.3(2)	C6	C7	C7A	N1	179.5(2)
C2	C3	C3A	C7A	0.2(2)	C7	C7A	N1	C2	-180.0(2)
C3	C2	N1	C7A	0.6(2)	C7	C7A	N1	C8	4.1(3)
C3	C2	N1	C8	176.6(2)	C7A	C3A	C4	C5	0.5(3)
C3	C3A	C4	C5	180.0(2)	C9	C8	N1	C2	116.9(3)
C3	C3A	C7A	C7	179.68(19)	C9	C8	N1	C7A	-67.7(3)
C3	C3A	C7A	N1	0.1(2)	N1	C2	C3	C3A	-0.5(2)
C3A	C4	C5	C6	0.4(3)	N1	C2	C3	O3	-179.8(2)
C3A	C7A	N1	C2	-0.5(2)	N1	C8	C9	C10	128.7(3)
C3A	C7A	N1	C8	-176.4(2)	O2	C2	C3	C3A	178.5(3)
C4	C3A	C7A	C7	-0.7(3)	O2	C2	C3	O3	-0.8(4)
C4	C3A	C7A	N1	179.68(19)	O2	C2	N1	C7A	-178.5(2)
C4	C5	C6	C7	-1.2(4)	O2	C2	N1	C8	-2.5(4)
C5	C6	C7	C7A	0.9(3)	O3	C3	C3A	C4	0.0(4)
C6	C7	C7A	C3A	0.0(3)	O3	C3	C3A	C7A	179.5(2)

Table S7. Hydrogen Atom Coordinates (Å×10^4) and Isotropic Displacement Parameters (Å^2×10^3) for compound 23.

Atom	*x*	*y*	*z*	U(eq)
H4	739.07	7634.26	4584.15	91
H5	3382.61	10430.67	4836.65	100
H6	6061.83	10279.57	3917.01	90
H7	6254.04	7278.74	2759.64	80
H8A	4388.86	1453.89	1363.55	104
H8B	6073.55	2632.85	2009.26	104
H9	4083.43	5695.57	658.86	115
H11A	9470(50)	5830(90)	180(30)	167(15)
H11B	9180(60)	8190(60)	950(30)	181(18)

Mass Spectra for New Compounds (18, 19, 20, 21, 22, 23, and 26)

Elemental Composition Report **Page 1**

Single Mass Analysis
Tolerance = 10.0 PPM / DBE: min = -1.5, max = 50.0
Element prediction: Off
Number of isotope peaks used for i-FIT = 3

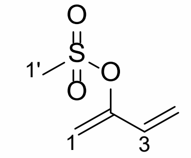

Compound **18**

Monoisotopic Mass, Odd and Even Electron Ions
90 formula(e) evaluated with 3 results within limits (up to 20 closest results for each mass)
Elements Used:
C: 2-27 H: 0-77 N: 0-11 O: 0-15 Na: 0-1 S: 1-1
DUCR 1.59
PK DUCRahayu DUCR 1-59 37 (0.901) Cm (36:40)

1: TOF MS ES+
1.69e+002

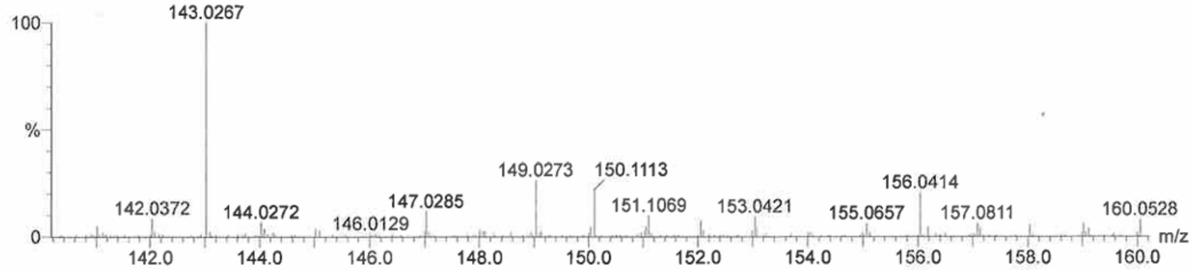

Minimum:				-1.5			
Maximum:		5.0	10.0	50.0			
Mass	Calc. Mass	mDa	PPM	DBE	i-FIT	i-FIT (Norm)	Formula
149.0273	149.0272	0.1	0.7	1.5	61.3	0.9	C5 H9 O3 S
	149.0275	-0.2	-1.3	3.0	61.2	0.8	C6 H8 N Na S
	149.0259	1.4	9.4	2.0	62.5	2.1	C3 H7 N3 O2 S

Figure S48. Mass spectrum of buta-1,3-dien-2-yl methanesulfonate **18**

Elemental Composition Report

Page 1

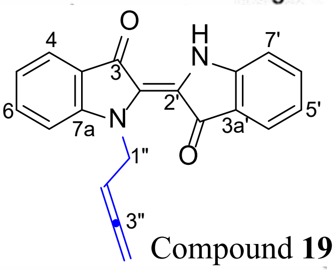

Compound **19**

Single Mass Analysis
Tolerance = 10.0 PPM / DBE: min = -1.5, max = 120.0
Element prediction: Off
Number of isotope peaks used for i-FIT = 3

Monoisotopic Mass, Even Electron Ions
549 formula(e) evaluated with 6 results within limits (all results (up to 1000) for each mass)
Elements Used:
C: 15-44 H: 0-88 N: 0-10 O: 0-12 Na: 0-1
DUCR P13-4
PK DUCRahayu DUCR P13-4 127 (3.029) Cm (124:130)

1: TOF MS ES+
3.00e+003

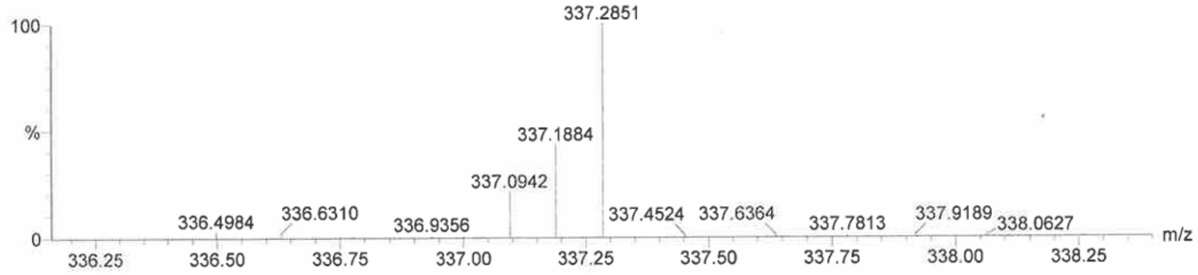

Minimum:				-1.5			
Maximum:		55.0	10.0	120.0			
Mass	Calc. Mass	mDa	PPM	DBE	i-FIT	i-FIT (Norm)	Formula
337.0942	337.0937	0.5	1.5	13.5	118.9	1.8	C17 H13 N4 O4
	337.0950	-0.8	-2.4	18.5	119.0	1.9	C18 H9 N8
	337.0953	-1.1	-3.3	14.5	119.2	2.1	C20 H14 N2 O2 Na
	337.0926	1.6	4.7	15.5	118.8	1.7	C16 H10 N8 Na
	337.0923	1.9	5.6	8.5	118.8	1.7	C16 H17 O8
	337.0913	2.9	8.6	10.5	118.7	1.6	C15 H14 N4 O4 Na

Figure S49. Mass spectrum of (*E*)-1-(buta-2,3-dien-1-yl)-[2,2'-biindolinylidene]-3,3'-dione **19**

Elemental Composition Report **Page 1**

Single Mass Analysis
Tolerance = 10.0 PPM / DBE: min = -1.5, max = 120.0
Element prediction: Off
Number of isotope peaks used for i-FIT = 3

Monoisotopic Mass, Even Electron Ions
698 formula(e) evaluated with 5 results within limits (all results (up to 1000) for each mass)
Elements Used:
C: 15-44 H: 0-88 N: 0-10 O: 0-12 Na: 0-1
DUCR P13-M6-4
PK DUCRahayu DUCR P13-M6-4 166 (3.949) Cm (165:166)

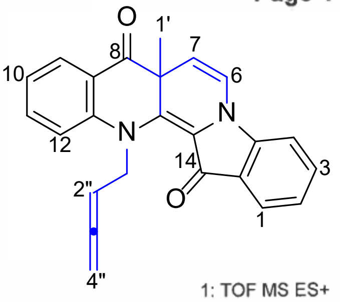

Compound **20**

1: TOF MS ES+
6.49e+002

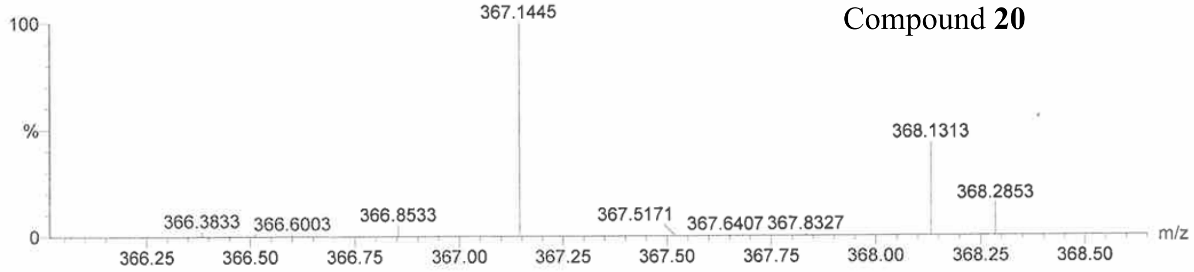

Mass	Calc. Mass	mDa	PPM	DBE	i-FIT	i-FIT (Norm)	Formula
Minimum:				-1.5			
Maximum:		55.0	10.0	120.0			
367.1445	367.1447	-0.2	-0.5	16.5	46.7	2.0	C24 H19 N2 O2
	367.1463	-1.8	-4.9	17.5	46.9	2.2	C27 H20 Na
	367.1422	2.3	6.3	13.5	46.2	1.5	C22 H20 N2 O2 Na
	367.1420	2.5	6.8	17.5	45.8	1.1	C20 H15 N8
	367.1481	-3.6	-9.8	4.5	46.4	1.7	C15 H24 N2 O7 Na

Figure S50. Mass spectrum of 13-(buta-2,3-dien-1-yl)-7a-methylbenzo[*b*]indolo[1,2-*h*][1,7]naphthyridine-8,14(7a*H*,13*H*)-dione **20**

Elemental Composition Report **Page 1**

Single Mass Analysis
Tolerance = 10.0 PPM / DBE: min = -1.5, max = 120.0
Element prediction: Off
Number of isotope peaks used for i-FIT = 3

Monoisotopic Mass, Even Electron Ions
453 formula(e) evaluated with 5 results within limits (all results (up to 1000) for each mass)
Elements Used:
C: 15-44 H: 0-88 N: 0-10 O: 0-12 Na: 0-1
DUCR P13-M8-17
PK DUCRahayu DUCR P13-M8-17 55 (1.329) Cm (53:55)

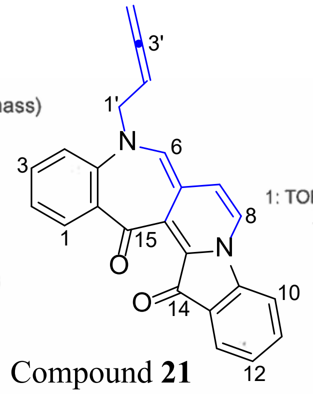

Compound **21**

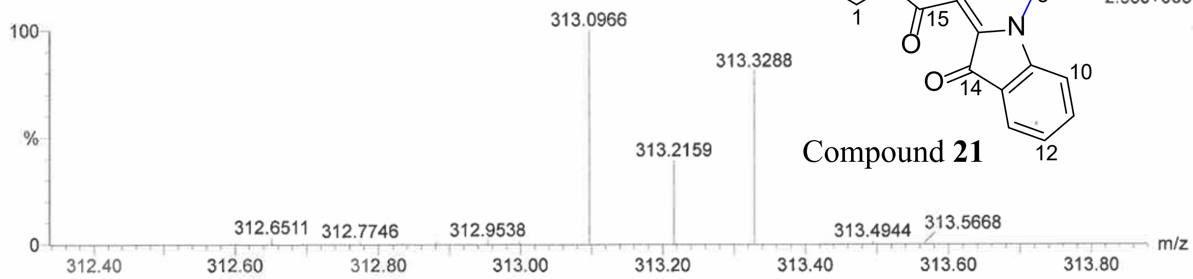

Minimum:				-1.5			
Maximum:		55.0	10.0	120.0			
Mass	Calc. Mass	mDa	PPM	DBE	i-FIT	i-FIT (Norm)	Formula
313.0966	313.0977	-1.1	-3.5	15.5	75.1	1.2	C20 H13 N2 O2
	313.0953	1.3	4.2	12.5	75.2	1.2	C18 H14 N2 O2 Na
	313.0950	1.6	5.1	16.5	75.4	1.4	C16 H9 N8
	313.0993	-2.7	-8.6	16.5	76.5	2.5	C23 H14 Na
	313.0937	2.9	9.3	11.5	76.3	2.4	C15 H13 N4 O4

Figure S51. Mass spectrum of 9*H*-benzo[6',7']azepino[2',3':3,4]pyrido[1,2-*a*]indole-9,15(14*H*)-dione **21**

Elemental Composition Report

Single Mass Analysis

Tolerance = 5.0 PPM / DBE: min = -1.5, max = 120.0
Element prediction: Off
Number of isotope peaks used for i-FIT = 3

Monoisotopic Mass, Even Electron Ions
843 formula(e) evaluated with 3 results within limits (up to 20 closest results for each mass)
Elements Used:
C: 18-39 H: 0-70 N: 0-15 O: 0-15 Na: 0-1
DUCR P73-12-3
PK DUCRahayu DUCR P73-12-3 122 (2.904) Cm (121:124)

1: TOF MS ES+
3.51e+002

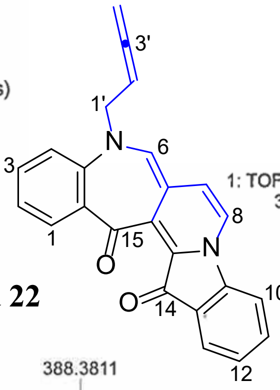

Compound **22**

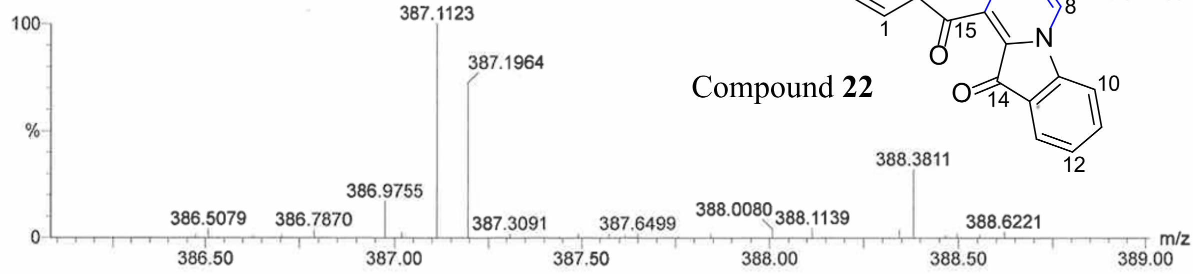

Minimum:				-1.5			
Maximum:		55.0	5.0	120.0			
Mass	Calc. Mass	mDa	PPM	DBE	i-FIT	i-FIT (Norm)	Formula
387.1123	387.1134	-1.1	-2.8	20.5	88.8	1.1	C26 H15 N2 O2
	387.1109	1.4	3.6	17.5	88.7	1.0	C24 H16 N2 O2 Na
	387.1107	1.6	4.1	21.5	88.8	1.2	C22 H11 N8

Figure S52. Mass spectrum of 5-(buta-2,3-dien-1-yl)-5*H*-benzo[6',7']azepino[4',3':3,4]pyrido[1,2-*a*]indole-14,15-dione **22**

Elemental Composition Report **Page 1**

Single Mass Analysis
Tolerance = 5.0 PPM / DBE: min = -1.5, max = 120.0
Element prediction: Off
Number of isotope peaks used for i-FIT = 3

Monoisotopic Mass, Even Electron Ions
283 formula(e) evaluated with 1 results within limits (up to 20 closest results for each mass)
Elements Used:
C: 11-39 H: 0-70 N: 0-15 O: 0-15 Na: 0-1
DUCR P41-3
PK DUCRahayu DUCR P41-3 62 (1.487) Cm (62:63)

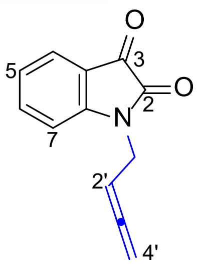

Compound **23**

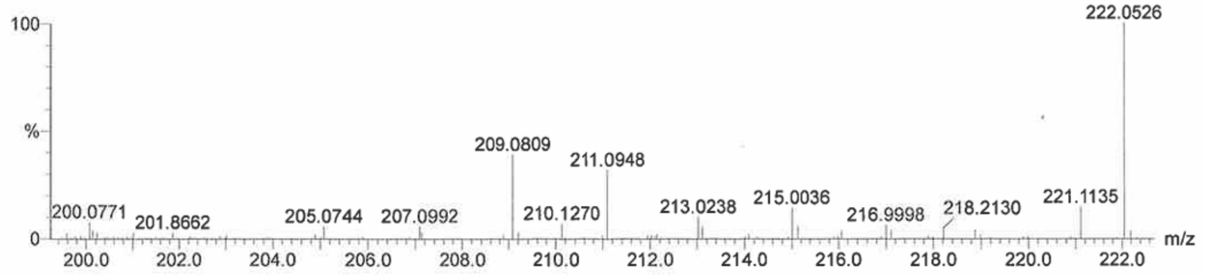

Minimum:				-1.5			
Maximum:		55.0	5.0	120.0			
Mass	Calc. Mass	mDa	PPM	DBE	i-FIT	i-FIT (Norm)	Formula
222.0526	222.0531	-0.5	-2.3	8.5	59.2	0.0	C12 H9 N O2 Na

Figure S53. Mass spectrum of 1-(buta-2,3-dien-1-yl)indoline-2,3-dione **23**

Elemental Composition Report **Page 1**

Single Mass Analysis
Tolerance = 5.0 PPM / DBE: min = -1.5, max = 120.0
Element prediction: Off
Number of isotope peaks used for i-FIT = 3

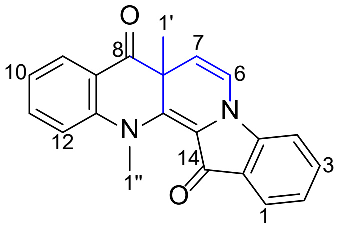

Compound **26**

Monoisotopic Mass, Even Electron Ions
125 formula(e) evaluated with 1 results within limits (all results (up to 1000) for each mass)
Elements Used:
C: 21-40 H: 15-80 N: 2-9 O: 2-9 Na: 0-1
DUCR-P-2-31(65-67)
PK_DRahayu_DUCR-P-2-31(65-67) 377 (8.925) Cm (368:381)

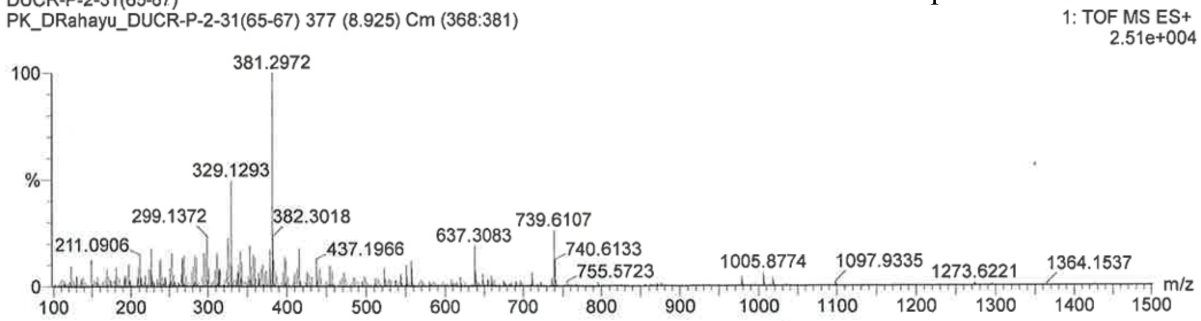

Minimum:				-1.5			
Maximum:		55.0	5.0	120.0			
Mass	Calc. Mass	mDa	PPM	DBE	i-FIT	i-FIT (Norm)	Formula
329.1293	329.1290	0.3	0.9	14.5	286.2	0.0	C21 H17 N2 O2

Figure S54. Mass spectrum of 7a,13-dimethylbenzo[*b*]indolo[1,2-*h*][1,7]naphthyridine-8,14(7a*H*,13*H*)-dione **26**
